# Mild Dehydrating Reagents in the Carbon Dioxide-Based Synthesis of Cyclic Carbamates from Amino Alcohols

**DOI:** 10.1021/acsomega.5c05230

**Published:** 2025-07-24

**Authors:** Jukka Puumi, Aleksi Sahari, Andrea Žáková, Jere K. Mannisto, Norbert M. Maier, Timo Repo

**Affiliations:** Department of Chemistry, 3835 University of Helsinki, A. I. Virtasen aukio 1, Helsinki 00014, Finland

## Abstract

Conventional CO_2_-based carboxylation of amino alcohols to cyclic carbamates is often hindered by poor chemo- and stereoselectivity, the need for high pressure or subzero temperatures, reliance on expensive or toxic reagents and catalysts, and the formation of hazardous waste. To address these limitations, we evaluated mild dehydrating reagents for their ability to promote the cyclization. Among the reagents tested, *n*-butyl chloride emerged as the optimal choice, offering a desirable combination of low cost, low toxicity, and high selectivity. In contrast to conventional highly reactive dehydrating reagents, *n*-BuCl provides high selectivity toward carbamate anion alkylation. Upon heating, the resulting linear carbamate intermediate cyclizes, liberating benign butanol and yielding almost exclusively stereoretentive five- and six-membered cyclic carbamates in 17–98% yields.

## Graphical abstract

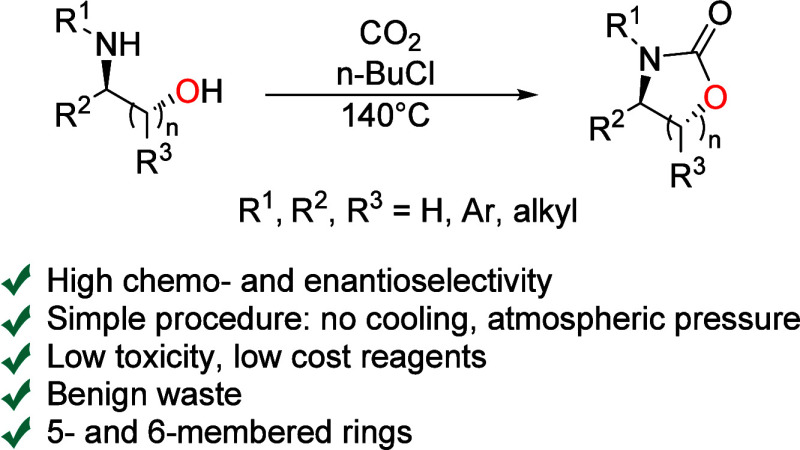

## Introduction

Cyclic carbamates are vital chemicals found in pharmaceuticals, synthetic intermediates, and chiral auxiliaries (Scheme 1A).^1^ The carbonyl group is conventionally sourced from phosgene-derived acylating reagents.^2^ More recently, carbon dioxide has emerged as an abundant and nontoxic alternative to these reagents. While CO_2_ provides the carbonyl carbon, the rest of the cyclic carbamate structure can be obtained from various sources such as amino alcohols,^3^ aziridines,^4^ alkenyl amines,^5^ and various dielectrophiles.^6^ Amino alcohols are especially attractive starting materials due to their stability and extensive synthetic availability. The known processes to generate cyclic carbamates from CO_2_ and amino alcohols can be broadly divided into catalytic (Scheme 1B) and stoichiometric approaches (Scheme 1C). Catalytic processes use a metal salt or organic catalyst and typically require high CO_2_ pressure and temperature.^3a,3^ They are also limited to very simple substitution patterns, especially those connected to the OH carbon. Stoichiometric processes utilize an electrophilic dehydrating reagent that facilitates the reaction. These include Mitsunobu reagents,^3k^ phosphorylating reagents,^3i,3j^ SOCl_2_,^3i,3l^ 4-toluenesulfonyl chloride (TsCl),^3m,3n^ and acetyl chloride (AcCl).^3i^ Due to their high reactivity, these reagents require low temperatures and extensive CO_2_ flushing to maintain high levels of chemo- and enantioselectivity. Despite these measures, direct N-alkylation or partial racemization at the chiral OH carbon is frequently observed.^3i,3m^ Racemization occurs due to poor control over the preferential intermolecular reaction of the dehydrating reagents at the available oxygen nucleophiles, i.e., the carbamate anion or alcohol (Scheme 1D). With the intention of developing simplified procedures for the CO_2_-based synthesis of cyclic carbamates from amino alcohols and simultaneously addressing the associated chemo- and stereoselectivity issues, we have systematically studied the suitability of a range of mild dehydrating reagents for this purpose. These investigations led to the identification of *n*-BuCl as an exceptionally mild and efficient reagent, providing the benefits of satisfactory yields, broad substrate applicability, and high level of chemo- and stereoselectivity due to its preference for selective carbamate anion attack (Scheme 1E).

**Scheme 1:**
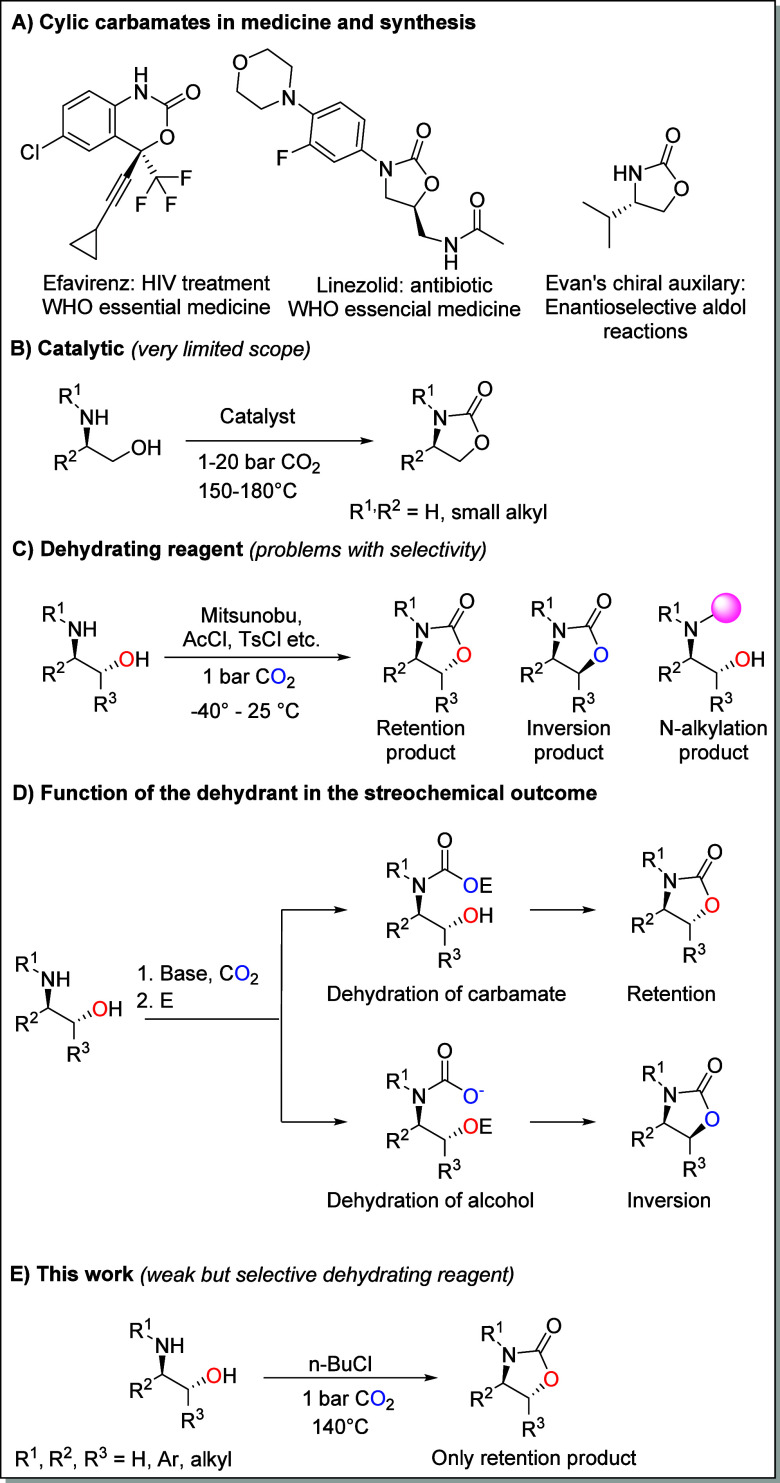
Cyclic Carbamates in Medicine and Synthesis and CO_2_-Based Strategies To Yield Them

## Results and Discussion

We initiated the study with an electrophile screen in DMSO using (+)-pseudoephedrine **1a**, *t*-BuOK and an electrophile in a 1:1:1 ratio (Table 1). The temperature was ramped up from room temperature by 10 °C/30 min, until 150 °C and kept there for 16 h. This was done to increase selectivity of reagents that have high reactivity but accordingly low selectivity.

**Table 1:**
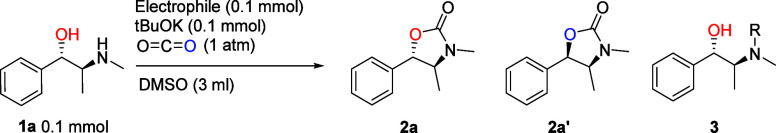
Screening of the Dehydrating Reagent

entry	electrophile	yield (%)^b^
1	*n*-BuCl	87
2	*n*-OctDecCl	79
3	*n*-BuBr	85
4	BnCl	30
5	*n*-BuI	10
6	*sec*-BuCl	37
7	*cyclo*-HexCl	11
8	*t*-BuCl	5
9	propylene oxide	57
10	(EtO)_3_Et	5
11	TMSCl	0
12	acrolein	6
13	2-benzylidene-malononitrile	8
14	nitrostyrene	11
15	methyl methacrylate	13
16	methyl cinnamate	12 (11)^c^
17	methyl benzoate	5 (23)^c^
18	benzyl benzoate	9 (25)^c^
19	diethyl succinate	9 (6)^c^
20	succinic anhydride	0
21	maleric anhydride	0
22[Table-fn t1fn4]	AcCl	0
23[Table-fn t1fn4]	TsCl	45
24[Table-fn t1fn4]	NsCl	31
25[Table-fn t1fn4]	TMSCl	0
26[Table-fn t1fn4]	I_2_	0

See the Experimental Section for a detailed description of the procedure. Conditions: 0.1 mmol of (+)-pseudoephedrine in 3 mL of DMSO. Then, CO_2_, base (0.1 mmol), and electrophile (0.1 mmol) were added. Temperature was raised from rt to 150 °C in 10 °C increments every 30 min and then left to 150 °C for 16 h.

a GC/MS yield with 0.1 mmol of mesitylene as internal standard.

b Inversion product **2a’** yield in brackets.

c In ACN, with DBU as the base, and started ramping from 0 to 120 °C.

Primary alkyl chlorides and bromides provided the product **2a** in high yields, but the more reactive benzyl chloride and butyl iodide provided primarily the direct N-alkylation product **3** (entries 1–5). Secondary and tertiary alkyl chlorides reacted much more slowly than primary ones, and the reactions did not reach completion (entries 6–8). Propylene oxide also worked quite well (entry 9). Less reactive orthoester (EtO)_3_Et provided very low amounts of product **2a** (entry 10). Trimethylsilyl chloride (TMSCl) provided no product (entry 11). Michael acceptors are potent and widely commercially available electrophiles, yet they only provided very low yields due to low conversion of **1a** (entries 12–16). Surprisingly, methyl cinnamate gave significant amounts of the inversion product **2a’** (entry 16). Similar behavior was observed with various other esters (entries 17–19). We propose that the inversion proceeds by an initial transesterification of the alcohol of **1a**, followed by an S_N_2-type ring closing (as shown in Scheme 1D). Interestingly, the retention product **2a** was also detected with all esters, which suggests that the ester-activated ring closing could partially proceed through an S_N_1-type mechanism. More reactive anhydrides failed to give neither **2a** nor **2a’**, yielding only N-alkylated product **3** (entries 20 and 21).

We also compared the reactivity of the abovementioned mild electrophiles to stronger electrophiles AcCl, TsCl, and 4-nitrobenzenesulfonyl chloride (NsCl), of which AcCl and TsCl have been previously used successfully for the synthesis of cyclic carbamates (entries 22–24).^3i,3m^ These reactions were done in ACN, with DBU as the base, starting from a temperature of 0 °C to avoid side reactions. Under these milder conditions, AcCl provided no product while TsCl and NsCl gave **2a** in low yields and produced large amounts of the N-alkylated products **3**. Interestingly, we detected no inversion product **2a’** with TsCl, although TsCl has been previously reported to give exclusively inversion products with a similar procedure.^3m^ As with standard conditions, TMSCl provided no **2a**. Finally, iodine, a good electrophile used in facilitating esterification, failed to mediate cyclization (entry 26).^7^

We further optimized the method with *n*-BuCl as its performance, low cost and toxicity, and benign waste product make it a highly desirable dehydrating reagent (Table 2). Slight increase in the base and *n*-BuCl equivalents gave a small increase in yield (entries 1 and 2 compared to entries 3 and 4). K_2_CO_3_, *t*-BuOK, and Cs_2_CO_3_ gave similar yields, while K_3_PO_4_, DBU, and *t*-BuONa were not as effective (entries 3–8). Screening of alternative solvents showed that only DMF provided a significant amount of product, while ACN and diglyme were very ineffective (entries 9–11). The complete optimization table is given in the Supporting Information, Table S1.

**Table 2:**
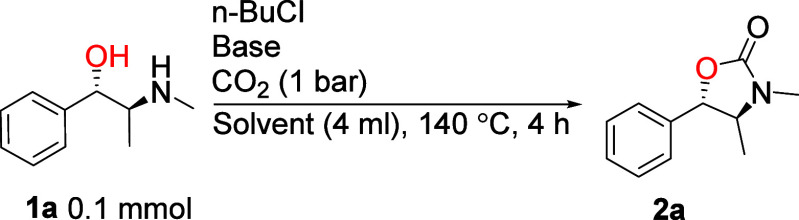
Optimization of the Cyclization Procedure

entry	base	solvent	ratios (base:bucl)	yield (%)[Table-fn t2fn2]
1	K_2_CO_3_	DMSO	1:1	74
2	*t*-BuOK	DMSO	1:1	87
3	K_2_CO_3_	DMSO	1.1:1.2	96
4	*t*-BuOK	DMSO	1.1:1.2	91
5	Cs_2_CO_3_	DMSO	1.1:1.2	87
6	K_3_PO_4_	DMSO	1.1:1.2	47
7	DBU	DMSO	1.1:1.2	18
8	*t*-BuONa	DMSO	1.1:1.2	15
9	*t*-BuOK	DMF	1.1:1.2	46
10	*t*-BuOK	ACN	1.1:1.2	<5
11	*t*-BuOK	diglyme	1.1:1.2	0

Full optimization and detailed experimental procedure are given in the Supporting Information, Section 3.1. Conditions: 0.1 mmol of (+)-pseudoephedrine in 4 mL of solvent. Then, added CO_2_, base (0.1–0.11 mmol), and BuCl (0.1–0.12 mmol). Stirred at 140 °C for 4 h.

a GC/MS yield with 0.1 mmol of mesitylene as the internal standard.

Based on the optimization and our initial trials with different substrates, we selected *t*-BuOK as the optimal base. As a stronger base, *t*-BuOK works better with less reactive amines than alkali carbonate salts and dissolves completely, which helps in achieving good reproducibility. For the scope studies, the concentration was increased 10-fold from the optimization concentration (0.025–0.25M), bringing the concentration to a reasonable level for synthesis and allowing the convenience of performing the scope reactions in small vials (General Procedure A).

Having established the optimal cyclization procedure, we next studied the scope of the reaction with different amino alcohols (Scheme 2). General Procedure A gave good to excellent yields with various N-substituted amino alcohols. As before, **2a** was isolated in near quantitative yield as a single diastereomer, indicating full retention of configuration on both chiral carbons. Scale up to gram scale did not affect the reaction in terms of yield and stereochemistry. The procedure tolerated primary (e.g., **2b**), secondary (e.g., **2a**), and tertiary carbons (**2d** and **2e**) next to the amine in the amino alcohol, although *tert*-butyl amino alcohol (**1e**) gave a poor yield, likely due to steric hindrance. The alcohol tolerated only primary (e.g., **2c**) and secondary carbons (e.g., **2f**). In the case of a tertiary alcohol, elimination took place (see compound **2aa**, Supporting Information, Section 7). Functional groups such as ether, trifluoromethyl, alkene, indole, and aryl bromide gave good yields (**2f**–**2j**) but typically required extended reaction times to reach full conversion (**2g**–**2j**). Esters and nitrile functionalities were hydrolyzed and partially butylated, yielding mixtures with only a trace amount of the desired cyclic carbamates (Supporting Information, Section 7).

**Scheme 2:**
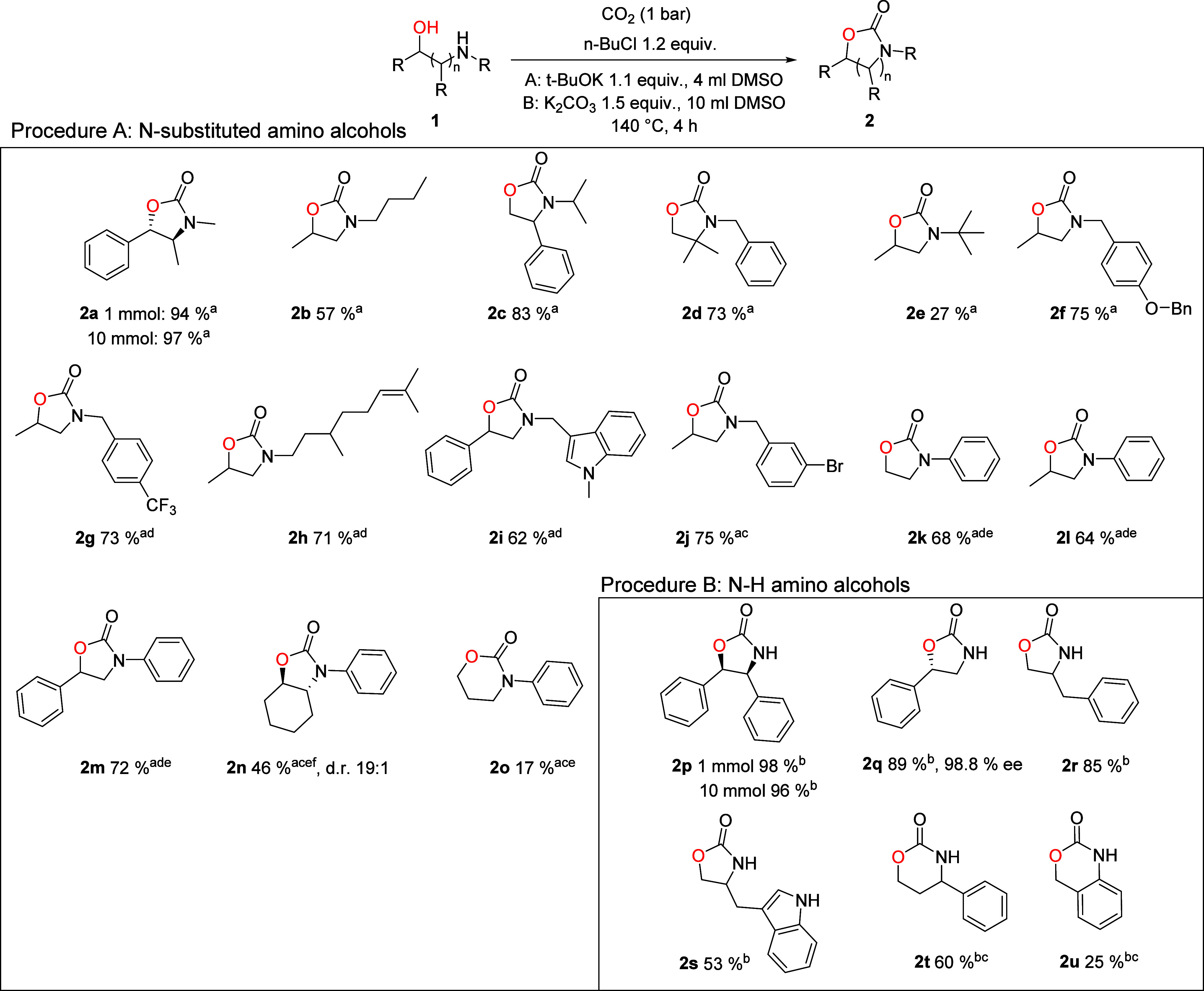
Substrate Scope of Amino Alcohols^a^,^b^,^c^,^d^,^e^,^f^,^g^

a See the Experimental Section for the detailed description of the conditions.
b Synthesized by General Procedure A.
c Synthesized by General Procedure B.
d Reaction time of 8 h.
e Reaction time of 24 h.
f Cs_2_CO_3_ instead of *t*-BuOK.
g 100 °C instead of 140 °C.

Drawing from our previous experience, for N-aryl amino alcohols, *t*-BuOK (General Procedure A) was replaced with the milder base Cs_2_CO_3_, providing higher yields.^6d^ This outcome is most likely due to the suppression of amine oxidation, which may take place with stronger bases due to partial deprotonation under air. With this modification, N-aryl amino alcohols were cyclized in good yields, but the reactions were slower compared to the alkyl amines (**2k**–**2o**). Noteworthily, a six-membered ring **2o** could also be constructed from the aromatic N-aryl amino alcohol **1o**, albeit in low yield. In the synthesis of **2n**, performing the reaction at 140 °C gave a 2:1 dr, favoring the retention product. Gratifyingly, lowering the temperature to 100 °C improved the dr to 19:1.

Amino alcohols bearing an unsubstituted amine (N–H amino alcohols) were also effectively coupled to a few key changes. General Procedure A displayed a low selectivity between N–H and N-Bu cyclic carbamate products. To suppress the formation of N-Bu cyclic carbamates, the reaction setup was changed to a Schlenk flask instead of a vial. This change reduced CO_2_ leaking, which we postulated to be the cause of the low selectivity with standard procedure A (see the discussion in the Supporting Information, Section 3.2). Consequently, 1.5 equiv of K_2_CO_3_ instead of 1.1 equiv of *t*-BuOK and 0.1 M solution instead of 0.25 M were used as these provided the best results. With this modification (General Procedure B), N–H amino alcohols were cyclized to oxazolidinones **2p**–**2s** in good to excellent yields. Again, full selectivity for the retention product was seen with **2p** and **2q**, and scale up to gram scale had no effect on the reaction in terms of yield or selectivity. As expected, General Procedure B also tolerated primary and secondary alcohols and an indole functionality. Apart from the five-membered rings, six-membered ring **2i**, derived from alkyl N–H amino alcohol **1i**, was produced in moderate yield, while a poor yield of **2u** was achieved with the aromatic N–H amino alcohol **1u**.

To investigate the mechanism of our synthetic procedure, we measured the reaction progress over time (Scheme 3). This analysis showed that (+)-pseudoephedrine **1a** reached completion in less than 1 h, forming cyclic carbamate **2a** in quantitative yield. During this time, we detected the formation and disappearance of butyl carbamate **4a**. In a separate reaction held at 80 °C for 12 h, butyl carbamate **4a** was formed as the major and **2a** as the minor product (Supporting Information, Section 6). This indicates that butyl carbamate intermediate **4a** is relatively stable and only cyclizes to **2a** at elevated temperatures. Noteworthily, no butyl ether side products were detected during the reaction progression determination or substrate scope study, indicating high selectivity of *n*-BuCl toward the carbamate anion over the alcohol group. During our substrate scope study, N-butylated products were observed in GC-MS with amino alcohol substrates such as **1b** and **1j** as very minor side products.

**Scheme 3:**
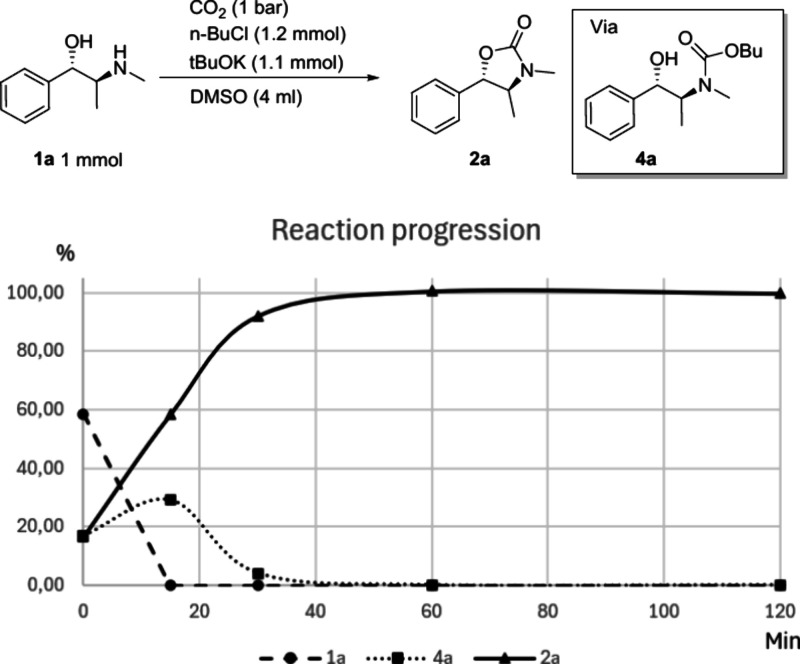
Reaction Progression Chart^a^

a Reaction was done according to General Procedure A. 0 min was when the reaction reached 140 °C, which was 7 min after starting the heating. See the Experimental Section for the detailed description of the procedure.

Based on the observations made during the reaction progression study and the fact that the reaction proceeds with retention, we propose the following reaction mechanism (Scheme 4). In the first step, carbon dioxide reacts with the amine of the amino alcohol **1** in the presence of a base to form a carbamate anion **5**. As the temperature rises, butyl chloride alkylates carbamate anion **5**, forming butyl carbamate intermediate **4**. The carbamate anion alkylation with alkyl halides is well established.^8^ The butyl carbamate intermediates are quite stable at moderate temperatures and can be easily isolated. However, when the temperature increases further, the OH group displaces the butyl group through a nucleophilic attack, expelling butanol and forming the more stable cyclic carbamate product **2** with retention of overall configuration.

**Scheme 4:**
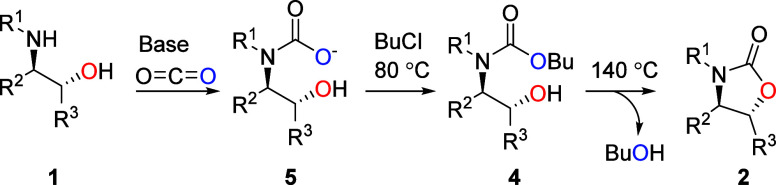
Proposed Mechanism

In conclusion, we have studied mild dehydrating reagents in carbon dioxide-based cyclization of amino alcohols and identified *n*-BuCl as a robust, selective, and inexpensive reagent. This primary alkyl chloride possesses a specific level of reactivity that enables high selectivity toward linear carbamate formation over possible N- or OH-alkylation byproducts. Upon heating, the linear carbamate is cyclized by expelling butanol, forming the desired cyclic carbamate with overall retention of the stereochemical configuration. Structurally diverse 1,2-amino alcohols are readily converted into oxazolidinones in good to excellent yields. 1,3-amino alcohols are also cyclized but in lower yields. Functional groups such as ethers, aryl halides, and alkenes are well tolerated, but hydrolysis-sensitive substrates such as esters and nitriles are not compatible with the presented protocol. As a major advantage over other reported procedures, our approach does not require subzero temperature, high CO_2_ pressure, or expensive and toxic reagents/catalysts and only produces benign *n*-BuOH as the organic side product.

## Experimental Section

### Procedure for Table 1, Entries 1–21

0.2 M **1a** ((+)-pseudoephedrine) and *t*-BuOK in dry DMSO were prepared and used within 1 week. To an 8 mL vial, 0.5 mL of the 0.2 M **1a** stock solution (0.1 mmol) and 3 mL of dry DMSO were added. The contents were sparged with CO_2_ (1 bar) for 10 s, after which 0.5 mL of the 0.2 M *t*-BuOK stock solution (0.1 mmol) was added. Subsequently, 0.1 mmol of electrophile was added in one portion to the vial. The vial was sealed with a rubber septum screw cap and further flushed with CO_2_ for 1 min. The mixture was stirred at room temperature for 30 min, and then the temperature was increased by 10 °C every 30 min until 150 °C was reached. At this temperature, the mixture was further stirred for 16 h. After the mixture was cooled to room temperature, 13.9 μL (0.1 mmol) of mesitylene was added, and an aliquot was analyzed with GC/MS to determine the yield.

### Procedure for Table 1, Entries 22–26

To an 8 mL vial, 16.5 mg (0.1 mmol) of **1a** ((+)-pseudoephedrine) and 3 mL of dry acetonitrile were added. 15.0 μL (0.1 mmol) of 1,8-diazabicyclo[5.4.0]­undec-7-ene (DBU) was added to the vial. The vial was sealed with a rubber septum screw cap, flushed with CO_2_ (1 bar) for 1 min, and cooled to 0 °C. 0.1 mmol of the electrophile was dissolved in 1 mL of dry acetonitrile and added to the vial dropwise. The mixture was stirred at 0 °C for 30 min, and then the temperature was increased by 10 °C every 30 min until 120 °C. At this temperature, the mixture was further stirred for 16 h. After cooling to room temperature, 13.9 μL (0.1 mmol) of mesitylene was added, and an aliquot was analyzed with GC/MS to determine the yield.

### General Procedure A

In an 8 mL vial, amino alcohol (1 mmol), *t*-BuOK (1.1 equiv, 1.1 mmol, 123 mg), and DMSO (4 mL) were added. The vial was sparged with CO_2_ (using a syringe connected to a Schlenk line, 1 bar) for approximately 10 s, closed with a screw cap, and allowed to mix for 1 min. The vial was sparged again for 10 s. *n*-BuCl (1.2 equiv, 1.2 mmol, 124.8 μL) was added. The vial was refilled with CO_2_ and closed again with a screw cap. The vial was subsequently heated from room temperature to 140 °C and maintained at that temperature for a specified reaction time. Afterward, the contents were transferred to a separatory funnel along with 100 mL of 0.2 M HCl and 50 mL of EtOAc and extracted once or twice, depending on how well the phases separated. The combined organic phase was washed with 100 mL of water and 50 mL of conc. NaCl solution and dried with Na_2_SO_4_, and the solvent was evaporated. If needed, the crude product was purified by filtering through a pad of silica or by using silica gel column chromatography.

The 10 mmol reaction of **2a** was done as above but in a 150 mL Schlenk flask and with a 10-fold amount of solvent and reagents. Isolation was done by adding the contents of the flask into 300 mL of 0.1 M HCl and extracting 3 times with 100 mL of EtOAc. The combined organic phases were washed with 70 mL of water and 70 mL of conc. NaCl and dried with Na_2_SO_4_. Evaporation of the solvent afforded 1.855 g (97%) of **2a**.

### General Procedure B

In a 25 mL Schlenk flask, amino alcohol (1 mmol), K_2_CO_3_ (1.5 equiv, 1.5 mmol, 207 mg), and DMSO (10 mL) were added. The flask was connected to the Schlenk line. The contents of the flask were sparged with CO_2_ (using a syringe connected to the Schlenk line, 1 bar) for about 10 s, closed with a stopper, and allowed to mix for 1 min. The flask was sparged again for 10 s. The valve of the flask was opened to fill the flask with CO_2_. *n*-BuCl (1.2 equiv., 1.2 mmol, 124.8 μL) was added under CO_2_ flow. The flask was then capped again with a glass stopper. The flask was heated from room temperature to 140 °C and maintained at that temperature for the specified reaction time. The flask was opened to the Schlenk line for the first 40 min of the reaction. After this period, the valve of the flask was closed. Once the reaction was complete, the mixture was transferred to a separatory funnel along with 70 mL of water and extracted with 4 × 30 mL of EtOAc. The combined organic phase was dried with Na_2_SO_4_, and the solvent was evaporated. If needed, the crude product was purified by filtering through a pad of silica or by using silica gel column chromatography.

The 10 mmol reaction of **2p** was done as above but in a 150 mL Schlenk flask and with a 10-fold amount of solvent and reagents. Isolation was done by adding the contents of the flask into 400 mL of water and extracting 4 times with 70 mL of EtOAc. The combined organic phases were dried with Na_2_SO_4_, and the solvent was evaporated to afford 2.285 g (96%) of **2p**.

### Reaction Progression Study

Five 8 mL vials were each loaded according to the General Procedure A. The vials were then heated to 140 °C for the specified reaction time. The timing was started when the target temperature of 140 °C was reached, which was 7 min after the heating had started (0 min data point). Vials were then removed from heating after 15, 30, 60, and 120 min from the starting point. After each vial had cooled to room temperature, 139 μL (1 mmol) of mesitylene was added, and an aliquot was analyzed with GC/MS to determine the yields of **1a**, **2a**, and **3a** in each vial.

## Supplementary Material



## Notes

Optimization, detailed synthetic and analysis procedures, and copies of NMR spectra (PDF)

## †

Present address: Charles University Prague, Chemistry Section, Prague 128 43, Czech Republic (A.Z.)
