# Supplementary material for: Mild Dehydrating Reagents in the Carbon Dioxide-Based Synthesis of Cyclic Carbamates from Amino Alcohols
Source: ACS Omega. 2025 Jul 24;10(30):33843–9. doi: 10.1021/acsomega.5c05230 (PMC12332661; doi:10.1021/acsomega.5c05230)
Supplement: Supplementary file 1 [file ao5c05230_si_001.pdf]

# Supporting information

# Mild dehydrating reagents in carbon dioxide-based synthesis of cyclic carbamates from amino alcohols

Jukka Puumi, Aleksi Sahari, Andrea Žáková, Jere K. Mannisto, Norbert M. Maier, Timo Repo*

Department of Chemistry

University of Helsinki

FI-00014 University of Helsinki, Finland

E-mail: timo.repo@helsinki.fi

* Corresponding author

# 1 CONTENTS

2 General information ... 2
3 Optimization ... 4
3.1 Optimization of N-substituted amino alcohols ... 4
3.2 Optimization of N-H amino alcohols ... 5
3.3 Behavior under excess of butyl chloride ... 6
4 Synthesis of cyclic carbamates ... 6
4.1 General procedures ... 6
4.2 Synthesis ... 8
5 Synthesis of amino alcohols ... 19
5.1 General procedures ... 19
5.2 Synthesis ... 20
6 Other synthesis ... 25
7 Failed synthesis ... 27
8 Chiral chromatography ... 31
9 NMR spectra ... 35
9.1 Carbamates ... 35
9.2 Amino alcohols ... 60
10 References ... 68

# 2 GENERAL INFORMATION

All solvents used in the optimization and synthesis were dried once over 3 Å molecular sieves. Some amino alcohol starting materials were synthetized from simple amino alcohols using methods described below. Other starting materials were received from chemical vendors and used without further purification.

NMR experiments were run with Bruker AS400 NMR spectrometer. $^{1}$H NMR spectra is reported relative to residual solvent peaks (7.26 ppm for $CHCl_3$ and 2.50 for DMSO).[1] $^{13}$C NMR spectra is reported relative to residual solvent peaks (77.16 ppm for $CHCl_3$ and 39.52 ppm for DMSO). GC yields were determined with Agilent technologies 7890B GC system coupled with Agilent 5977B Mass Selective Detector (EI). GC-MS experiments were run with

Agilent 6890N Network Gas Chromatograph coupled with Agilent 5973 Network Mass Selective Detector (EI). HRMS (ESI) was recorded with Agilent 6230 LC/TOF system.

# 3 OPTIMIZATION

## 3.1 OPTIMIZATION OF N-SUBSTITUTED AMINO ALCOHOLS

*Table S1. Optimization of N-substituted amino alcohols.*

| Entry | Base | Solvent | Ratios (Base : BuCl) | Yield (%)[a] |
|---|---|---|---|---|
| 1 | $K_2CO_3$ | DMSO | 1:1 | 74 |
| 2 | t-BuOK | DMSO | 1:1 | 87 |
| 3 | $K_3PO_4$ | DMSO | 1:1 | 54 |
| 4 | $Cs_2CO_3$ | DMSO | 1:1 | 64 |
| 5 | $K_2CO_3$ | DMSO | 1.1 : 1.2 | 96 |
| 6 | t-BuOK | DMSO | 1.1 : 1.2 | 91 |
| 7 | $K_3PO_4$ | DMSO | 1.1 : 1.2 | 47 |
| 8 | $Cs_2CO_3$ | DMSO | 1.1 : 1.2 | 87 |
| 9 | tBuONa | DMSO | 1.1 : 1.2 | 15 |
| 10 | $Na_2CO_3$ | DMSO | 1.1 : 1.2 | 0 |
| 11 | $Li_2CO_3$ | DMSO | 1.1 : 1.2 | 0 |
| 12 | DBU | DMSO | 1.1 : 1.2 | 18 |
| 13 | $K_2CO_3$ | DMF | 1.1 : 1.2 | 14 |
| 14 | t-BuOK | DMF | 1.1 : 1.2 | 46 |
| 15 | $Cs_2CO_3$ | DMF | 1.1 : 1.2 | 57 |
| 16 | $K_2CO_3$ | ACN | 1.1 : 1.2 | 0 |
| 17 | t-BuOK | ACN | 1.1 : 1.2 | < 5 |
| 18 | $Cs_2CO_3$ | ACN | 1.1 : 1.2 | 0 |
| 19 | $K_2CO_3$ | Diglyme | 1.1 : 1.2 | 10 |
| 20 | t-BuOK | Diglyme | 1.1 : 1.2 | 0 |
| 21 | $Cs_2CO_3$ | Diglyme | 1.1 : 1.2 | 12 |
| 22 | $Cs_2CO_3$ | Sulfolane | 1.1 : 1.2 | 0 |
| 23 | $Cs_2CO_3$ | EtOAc | 1.1 : 1.2 | 0 |

To an 8 ml vial were added 16.5 mg (0.1 mmol) of (+)-pseudoephedrine, 0.11 mmol (1.1 equiv.) of a base and 4 ml of solvent. The mixture was flushed with $CO_2$ for 10 seconds. 0.12 mmol of n-BuCl (1.2 equiv.) was added. The vial was sealed with a rubber septum screw cap and flushed with $CO_2$ for 1 minute. The mixture was heated at 140°C for 4 hours. After cooling to room temperature, 13.9 µl (0.1 mmol) of mesitylene was added and an aliquot was taken and analyzed with GC/MS to determine the yield.

## 3.2 Optimization of N-H amino alcohols

During initial testing we had varying result with N-H amino alcohols such as **1t** when doing the reaction in vials according to the general procedure A. N-H amino alcohols had tendencies to form mixtures of N-H and N-Bu cyclic carbamates in varying ratios even in seemingly identical conditions. We postulated that this was caused by leaking of the vials, causing decarboxylation and formation of the free amine form. This free amine form would then react with BuCl to form N-Bu amine which would then go on to form the cyclic carbamate with $CO_2$ and another equivalent of BuCl. This N alkylation of the free amine is only problematic with N-H amino alcohols likely because RHN-$COO^-$ is less stable than $R_2$N-$COO^-$ making it more prone to decarboxylation due to increased temperature and decreased $CO_2$ pressure.[2] $R_2$N-$COO^-$ anions are also expected to be more nucleophilic than RHN-$COO^-$ and therefore are alkylated at lower temperature, making it less likely that leaking takes place before the stable O-Bu carbamate intermediate is formed.

To have reproducible results, we found it is better to do N-H amino alcohol cyclization reactions in Schlenk flasks where a positive pressure can be kept (flask open to the Schlenk line) during the initial O-Bu carbamate intermediate formation step. The optimization of N-H amino alcohols in Schlenk flask is shown in Table S2.

*Table S2. Optimization of N-H amino alcohols*

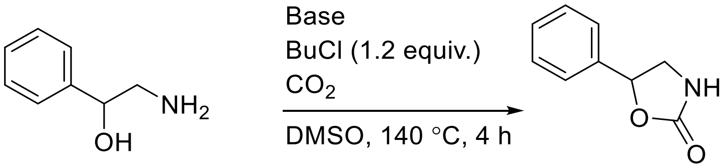


| Test | Base (equiv.) | NMR yield |
|---|---|---|
| 1 | t-BuOK (1.1) | 49 % |
| 2 | t-BuOK (1.5) | 65 % |
| 3 | $K_2CO_3$ (1.5) | 73 % |
| 4 | $K_2CO_3$ (2) | 72 % |

Into a 10 ml Schlenk flask were added 2-amino-1-phenylethan-1-ol (54.8 mg, 0.4 mmol), DMSO (10 ml) and base. $CO_2$ was then bubbled for 10 seconds, allowed to mix for 1 minute and bubbled again with $CO_2$. BuCl (50 µl, 0.48 mmol, 1.2 equiv.) was then added. The flask was filled with $CO_2$ and was left open to the Schlenk line. The flask was then heated from room temperature to 140 °C and left there for 4 hours. Afterwards, the contents were transferred to a separatory flask along with 50 ml of water and extracted with 20 ml of EtOAc. The combined organic phase was evaporated and further dried in an oil pump overnight. The residue was dissolved in $CDCl_3$ and 0.4 mmol of DMF was added as an internal standard. The solution was analyzed immediately with $^1$H NMR to determine the yield.

Since higher excess of base was required to get full conversion of N-H amino alcohols as compared to N-substituted amino alcohols and because $K_2CO_3$ gave corresponding yield to t-BuOK, we decided to use $K_2CO_3$ due to safety reasons. A strong base such as t-BuOK is more likely to cause thermal runaway problems with DMSO and the problem is aggravated when higher excess of base is present.

### 3.3 BEHAVIOR UNDER EXCESS OF BUTYL CHLORIDE

When done in 8 ml vials in highly concentrated solution with high excess of BuCl, N-Bu oxazolidinones are formed as major products form N-H amino alcohols. This behavior is consistent with the above discussion on the selectivity issues between N-H and N-Bu oxazolidinone products.

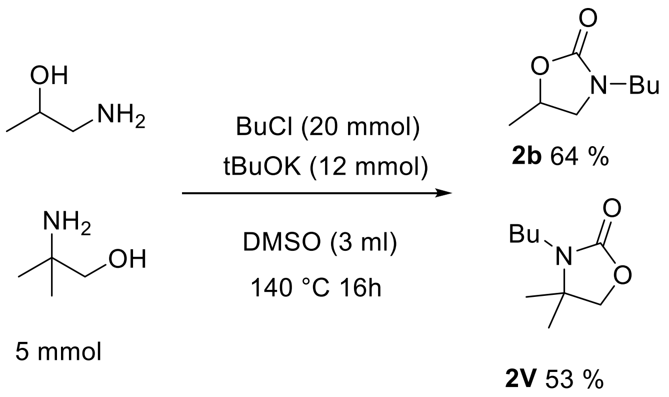


*Scheme S1. Behavior of the N-H amino alcohols under excess of butyl chloride in a concentrated solution.*

## 4 SYNTHESIS OF CYCLIC CARBAMATES

### 4.1 GENERAL PROCEDURES

**General procedure A**

In an 8 ml vial were added amino alcohol (1 mmol), t-BuOK (1.1 equiv, 1.1 mmol, 123 mg) and DMSO (4 ml). The vial was sparged with $CO_2$ (using a syringe connected to a Schlenk line, 1 bar) for approximately 10 seconds, closed with a screw cap and allowed to mix for one minute. The vial was sparged again for 10 seconds. n-BuCl (1.2 equiv., 1.2 mmol, 124.8 µl)

was added. The vial was refilled with $CO_2$ and closed again with a screw cap. The vial was subsequently heated from room temperature to 140 °C and maintained at that temperature for the specified reaction time. Afterwards, the contents were transferred to a separatory funnel along with 100 ml of 0.2 M HCl and 50 ml of EtOAc and extracted once or twice, depending on how well the phases separated. The combined organic phase was washed with 100 ml of water and 50 ml of conc. NaCl solution, dried with $Na_2SO_4$ and the solvent was evaporated. If needed, the crude product was purified by filtering through a pad of silica or by using silica gel column chromatography.

**General procedure B**

In a 25 ml Schlenk flask were added amino alcohol (1 mmol), $K_2CO_3$ (1.5 equiv., 1.5 mmol, 207 mg) and DMSO (10 ml). The flask was connected to the Schlenk line. The contents of the flask were sparged with $CO_2$ (using a syringe connected to the Schlenk line, 1 bar) for about 10 seconds, closed with a stopper and allowed to mix for one minute. The flask was sparged again for 10 seconds. The valve of the flask was opened to fill the flask with $CO_2$. n-BuCl (1.2 equiv., 1.2 mmol, 124.8 μl) was added under $CO_2$ flow. The flask was then capped again with the glass stopper. The flask was heated from room temperature to 140 °C and maintained at that temperature for the specified reaction time. The flask was opened to the Schlenk line for the first 40 minutes of the reaction. After this period, the valve of the flask was closed. Once the reaction was complete, the mixture was transferred to a separatory funnel along with 70 ml of water, and extracted with 4 x 30 ml of EtOAc. The combined organic phase was dried with $Na_2SO_4$ and the solvent was evaporated. If needed, the crude product was purified by filtering through a pad of silica or by using silica gel column chromatography.

**Procedure for filtering through silica**

A 60 ml filter crucible was filled about halfway with silica gel and placed on top of a straight vacuum take-off adapter joined with a receiving flask (Figure S1). The silica gel was wetted with DCM. The crude product, dissolved in small amount of DCM, was pipetted on top of the silica evenly across its area. Vacuum was drawn for a few second to load the sample on the silica and then released. About 40 ml of DCM was carefully poured on top of the silica, and vacuum was drawn until no more DCM eluted. The receiving flask was changed, and the procedure was repeated with EtOAc to elute the purified product.

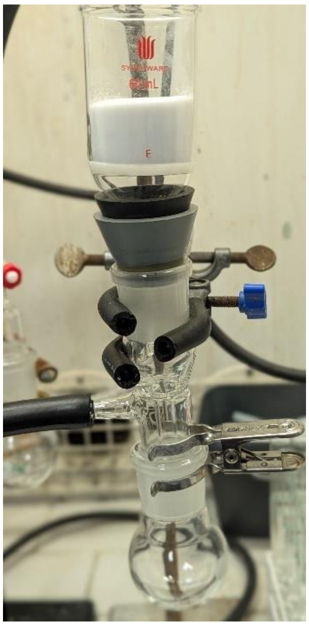

*Figure S1. Setup for purification by filtering through silica.*

## 4.2 SYNTHESIS

(4S,5S)-3,4-dimethyl-5-phenyloxazolidin-2-one (**2a**)

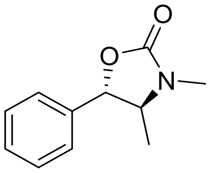

Synthetized according to the general procedure A. Reaction time 4 hours. Yield 179.7 mg (94 %). Product is a white solid.

Also prepared at a 10 mmol scale in a 150 ml Schlenk flask. Yield 1.855 g (97 %).

Recorded NMR spectra matches the reported spectra.[3]

$^{1}$H NMR (400 MHz, $CDCl_3$) δ 7.44 – 7.32 (m, 5H), 4.90 (d, $J$ = 7.8 Hz, 1H), 3.54 (dq, $J$ = 7.8, 6.2 Hz, 1H), 2.86 (s, 3H), 1.36 (d, $J$ = 6.1 Hz, 3H).

$^{13}$C NMR (101 MHz, $CDCl_3$) δ 157.9, 137.8, 129.1, 129.0, 126.0, 82.6, 61.4, 28.9, 17.5.

3-butyl-5-methyloxazolidin-2-one (**2b**)

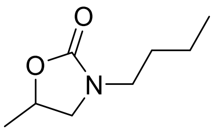

Synthetized according to the general procedure A. Reaction time 24 hours. Further purified by filtering through silica. Yield 90.1 mg (57 %). Product is a brown oil. Recorded NMR spectra matches the reported spectra.[4]

Prepared also from the corresponding N-H amino alcohol in a scale of 5 mmol using 20 mmol of n-BuCl, 12 mmol of t-BuOK and 3 ml of DMSO (Scheme S1). Reaction time 16 hours. Isolation was done according to general procedure A. Yield 503.0 mg (64 %).

$^{1}$H NMR (400 MHz, $CDCl_3$) δ 4.68 – 4.55 (m, 1H), 3.62 (t, *J* = 8.3 Hz, 1H), 3.35 – 3.16 (m, 2H), 3.10 (dd, *J* = 8.4, 6.9 Hz, 1H), 1.56 – 1.47 (m, 2H), 1.42 (d, *J* = 6.3 Hz, 3H), 1.39 – 1.29 (m, 2H), 0.94 (t, *J* = 7.3 Hz, 3H).

$^{13}$C NMR (101 MHz, $CDCl_3$) δ 158.2, 69.9, 51.5, 43.9, 29.6, 20.9, 20.0, 13.9.

3-isopropyl-4-phenyloxazolidin-2-one (**2c**)

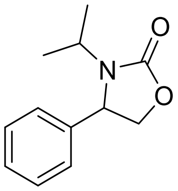

Synthetized according to the general procedure A. Reaction time 4 hours. Yield 170.1 mg (83 %). Product is a brown oil.

$^{1}$H NMR (400 MHz, $CDCl_3$) δ 7.42 – 7.28 (m, 5H), 4.78 (dd, *J* = 9.0, 6.5 Hz, 1H), 4.54 (td, *J* = 8.9, 1.1 Hz, 1H), 4.10 – 4.01 (m, 1H), 3.79 (heptd, *J* = 6.9, 1.4 Hz, 1H), 1.23 (dd, *J* = 6.9, 1.2 Hz, 3H), 0.91 (dd, *J* = 6.9, 1.1 Hz, 3H).

$^{13}$C NMR (101 MHz, $CDCl_3$) δ 157.9, 140.1, 129.1, 128.9, 127.1, 70.3, 59.0, 46.5, 20.6, 19.7.

HRMS-ESI (pos): $[M + H]^+$ calculated for $C_{12}H_{16}NO_2$ 206.1176, found 206.1168.

3-benzyl-4,4-dimethyloxazolidin-2-one (**2d**)

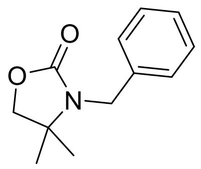


Synthetized according to the general procedure A.  Reaction time 4 hours. Yield 150.0 mg (73 %). Product is a brown oil.

$^{1}$H NMR (400 MHz, $CDCl_3$) δ 7.36 – 7.22 (m, 5H), 4.38 (s, 2H), 3.98 (s, 2H), 1.17 (s, 6H).

$^{13}$C NMR (101 MHz, $CDCl_3$) δ 158.2, 138.4, 128.6, 127.8, 127.6, 75.2, 58.7, 44.1, 25.4.

HRMS-ESI (pos): $[M + Na]^+$ calculated for $C_{12}H_{15}NO_2Na$ 228.0995, found 228.0999.

3-(tert-butyl)-5-methyloxazolidin-2-one (**2e**)

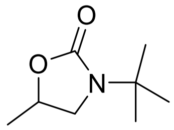


Synthetized according to the general procedure A. Reaction time 24 hours. Further purified by filtering through silica. Yield 42.0 mg (27 %). Product is a yellow-brown oil.

$^{1}$H NMR (400 MHz, $CDCl_3$) δ 4.50 (dh, $J$ = 12.7, 6.0 Hz, 1H), 3.66 (t, $J$ = 8.1 Hz, 1H), 3.14 (t, $J$ = 7.9 Hz, 1H), 1.38 (s, 1H), 1.37 (s, 13H).

$^{13}$C NMR (101 MHz, $CDCl_3$) δ 157.1, 68.9, 53.3, 50.3, 27.5, 20.4.

HRMS-ESI (pos): $[M + Na]^+$ calculated for $C_8H_{15}NO_2Na$ 180.0995, found 180.0987.

3-(4-(benzyloxy)benzyl)-5-methyloxazolidin-2-one (**2f**)

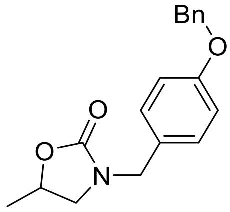


Synthetized according to the general procedure A.  Reaction time 4 hours. Yield 224.6 mg (75 %). Product is a white solid.

$^{1}H$ NMR (400 MHz, $CDCl_3$) δ 7.47 – 7.29 (m, 5H), 7.24 – 7.16 (m, 2H), 7.01 – 6.91 (m, 2H), 5.06 (s, 2H), 4.60 (dp, *J* = 8.2, 6.3 Hz, 1H), 4.42 – 4.28 (m, 2H), 3.47 (t, *J* = 8.4 Hz, 1H), 2.95 (dd, *J* = 8.5, 6.9 Hz, 1H), 1.38 (d, *J* = 6.2 Hz, 3H).

$^{13}C$ NMR (101 MHz, $CDCl_3$) δ 158.6, 158.1, 136.8, 129.5, 128.6, 128.2, 128.1, 127.5, 115.1, 70.1, 70.1, 50.6, 47.7, 20.7.

HRMS-ESI (pos): $[M + H]^+$ calculated for $C_{18}H_{20}NO_3$ 298.1438, found 298.1427.

5-methyl-3-(4-(trifluoromethyl)benzyl)oxazolidin-2-one (**2g**)

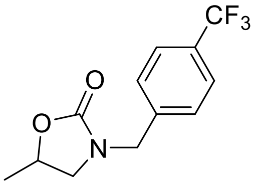


Synthetized according to the general procedure A. Reaction time 24 hours. Purified by silica column chromatography with EtOAc in hexane as eluent. Yield 189.1 mg (73 %). Product is a white solid.

$^{1}H$ NMR (400 MHz, $CDCl_3$) δ 7.65 – 7.58 (m, 2H), 7.44 – 7.37 (m, 2H), 4.71 – 4.58 (m, 1H), 4.57 – 4.40 (m, 2H), 3.51 (t, *J* = 8.3 Hz, 1H), 2.99 (dd, *J* = 8.5, 6.9 Hz, 1H), 1.41 (d, *J* = 6.2 Hz, 3H).

$^{13}C$ NMR (101 MHz, $CDCl_3$) δ 158.3, 140.1, 130.3 (q, *J* = 32.5 Hz), 128.4, 126.0 (q, *J* = 3.8 Hz), 124.1 (q, *J* = 272.2 Hz), 70.3, 51.0, 48.0, 20.8.

HRMS-ESI (pos): $[M + Na]^+$ calculated for $C_{12}H_{12}F_3NO_2Na$ 282.0712, found 282.0716.

3-(3,7-dimethyloct-6-en-1-yl)-5-methyloxazolidin-2-one (**2h**)

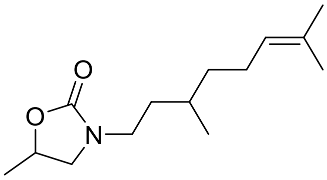


The starting material was prepared from (+/-)-citronellal and racemic 1-aminopropan-2-ol so this product a mixture of diastereomers which is easiest seen from the doublet signals in $^{13}C$ NMR. The diastereomers were not easily separated on column chromatography.

Synthetized according to the general procedure A. Reaction time 24 hours. Further purified by filtering through silica. Yield 169.9 mg (71 %). Product is a clear oil.

$^{1}$H NMR (400 MHz, $CDCl_3$) δ 5.07 (tdq, *J* = 7.3, 2.9, 1.4 Hz, 1H), 4.67 – 4.54 (m, 1H), 3.61 (td, *J* = 8.3, 5.0 Hz, 1H), 3.37 – 3.12 (m, 2H), 3.08 (ddd, *J* = 8.4, 6.9, 5.9 Hz, 1H), 2.07 – 1.87 (m, 2H), 1.69 – 1.65 (m, 3H), 1.59 (d, *J* = 1.3 Hz, 3H), 1.58 (s, 1H), 1.58 – 1.43 (m, 1H), 1.41 (dd, *J* = 6.3, 1.4 Hz, 3H), 1.39 – 1.08 (m, 4H), 0.96 – 0.87 (m, 3H).

$^{13}$C NMR (101 MHz, $CDCl_3$) δ 158.1, 131.6 (d, *J* = 1.5 Hz), 124.6, 69.9 (d, *J* = 4.3 Hz), 51.4 (d, *J* = 4.5 Hz), 42.4 (d, *J* = 3.4 Hz), 37.1, 34.4, 30.1 (d, *J* = 2.2 Hz), 25.8, 25.5, 20.9 (d, *J* = 3.9 Hz), 19.4, 17.8.

HRMS-ESI (pos): $[M + Na]^+$ calculated for $C_{14}H_{25}NO_2Na$ 262.1778, found 262.1782.

3-((1-methyl-1H-indol-3-yl)methyl)-5-phenyloxazolidin-2-one (**2i**)

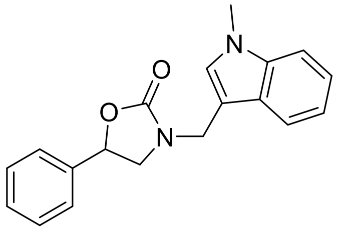


Synthetized according to the general procedure A. Reaction time 24 hours. Purified by silica column chromatography with EtOAc in hexane as eluent. Yield 190.1 mg (62 %). Product is a brown viscous oil.

$^{1}$H NMR (400 MHz, $CDCl_3$) δ 7.35 (dt, *J* = 7.9, 1.0 Hz, 1H), 7.26 (s, 0H), 6.97 – 6.84 (m, 7H), 6.79 (ddd, *J* = 8.0, 6.8, 1.2 Hz, 1H), 6.67 (s, 1H), 4.97 (dd, *J* = 8.8, 7.6 Hz, 1H), 4.36 (dd, *J* = 14.8, 0.7 Hz, 1H), 4.21 (dd, *J* = 14.8, 0.7 Hz, 1H), 3.37 (s, 3H), 3.36 (t, *J* = 8.8 Hz, 1H), 2.91 (dd, *J* = 8.8, 7.6 Hz, 1H).

$^{13}$C NMR (101 MHz, $CDCl_3$) δ 157.8, 138.8, 137.2, 128.8, 128.7, 128.6, 127.2, 125.6, 122.2, 119.7, 119.1, 109.5, 108.8, 74.5, 51.4, 39.4, 32.8.

HRMS-ESI (pos): $[M + Na]^+$ calculated for $C_{19}H_{18}N_2O_2Na$ 329.1260, found 329.1272.

3-(3-bromobenzyl)-5-methyloxazolidin-2-one (**2j**)

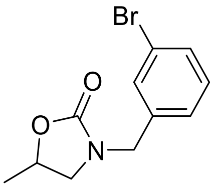


Synthetized according to the general procedure A. Reaction time 8 hours. Purified by silica column chromatography with EtOAc in hexane as eluent. Yield 212.8 mg (79 %). Product is a white solid.

$^{1}$H NMR (400 MHz, $CDCl_3$) δ 7.48 – 7.33 (m, 2H), 7.25 – 7.09 (m, 2H), 4.63 (dp, *J* = 8.2, 6.4 Hz, 1H), 4.46 – 4.24 (m, 2H), 3.50 (t, *J* = 8.4 Hz, 1H), 2.98 (dd, *J* = 8.5, 6.9 Hz, 1H), 1.39 (d, *J* = 6.2 Hz, 3H).

$^{13}$C NMR (101 MHz, $CDCl_3$) δ 158.2, 138.4, 131.2, 131.1, 130.6, 126.8, 123.0, 70.3, 50.9, 47.8, 20.8.

HRMS-ESI (pos): $[M + Na]^+$ calculated for $C_{11}H_{12}BrNO_2Na$ 291.9944, found 291.9942.

3-phenyloxazolidin-2-one (**2k**)

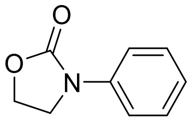


Synthetized according to the general procedure A using $Cs_2CO_3$ instead of t-BuOK. Reaction time 24 hours. Yield 110.1 mg (68 %). Product is a white solid. Recorded NMR spectra matches the reported spectra.[5]

$^{1}$H NMR (400 MHz, $CDCl_3$) δ 7.50 (dt, *J* = 8.7, 1.3 Hz, 2H), 7.34 (tt, *J* = 7.2, 1.0 Hz, 2H), 7.11 (td, *J* = 7.4, 1.2 Hz, 1H), 4.39 (tt, *J* = 8.0, 1.6 Hz, 2H), 4.01 – 3.91 (m, 2H).

$^{13}$C NMR (101 MHz, $CDCl_3$) δ 155.3, 138.2, 129.0, 123.9, 118.1, 61.3, 45.1.

5-methyl-3-phenyloxazolidin-2-one (**2l**)

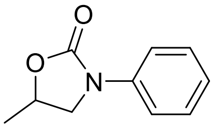

Synthetized according to the general procedure A using $Cs_2CO_3$ instead of t-BuOK. Reaction time 24 hours. Yield 114.1 mg (64 %). Product is a white solid. Recorded NMR spectra matches the reported spectra.[5]

$^1$H NMR (400 MHz, $CDCl_3$) δ 7.57 – 7.49 (m, 2H), 7.43 – 7.32 (m, 2H), 7.13 (tq, *J* = 7.3, 0.9 Hz, 1H), 4.83 – 4.70 (m, 1H), 4.09 (td, *J* = 8.5, 1.1 Hz, 1H), 3.60 (ddt, *J* = 8.7, 7.0, 0.8 Hz, 1H), 1.52 (dd, *J* = 6.2, 1.0 Hz, 3H).

$^{13}$C NMR (101 MHz, $CDCl_3$) δ 154.9, 138.4, 129.0, 123.9, 118.2, 69.6, 51.8, 20.7.

3,5-diphenyloxazolidin-2-one (**2m**)

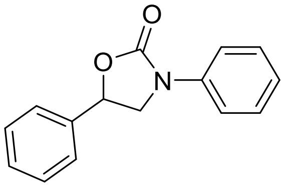

Synthetized according to the general procedure A with $Cs_2CO_3$ instead of t-BuOK. Reaction time 24 hours. Yield 172.3 mg (72 %). Product is a light yellow solid. Recorded NMR spectra matches the reported spectra.[6]

$^1$H NMR (400 MHz, $CDCl_3$) δ 7.60 – 7.52 (m, 2H), 7.52 – 7.32 (m, 7H), 7.22 – 7.11 (m, 1H), 5.62 (dd, *J* = 8.7, 7.4 Hz, 1H), 4.37 (t, *J* = 8.8 Hz, 1H), 3.95 (dd, *J* = 9.0, 7.5 Hz, 1H).

$^{13}$C NMR (101 MHz, $CDCl_3$) δ 154.8, 138.2, 138.2, 129.2, 129.1, 125.8, 124.3, 118.4, 74.1, 52.8.

(trans)-3-phenylhexahydrobenzo[d]oxazol-2(3H)-one **(2n)**

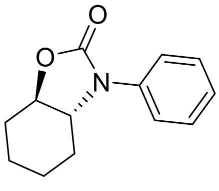

Synthetized according to the general procedure A using $Cs_2CO_3$ used instead of t-BuOK and temperature of 100 °C instead of 140 C°. Reaction time 24 hours. Yield 101.0 mg (46 %). Product is a clear oil. With this method about 95:5 trans:cis was recovered.

$^1$H NMR (400 MHz, $CDCl_3$) δ 7.44 – 7.38 (m, 2H), 7.33 – 7.20 (m, 3H), 4.03 (ddd, *J* = 11.9, 10.8, 3.6 Hz, 1H), 3.68 (td, *J* = 10.9, 3.3 Hz, 1H), 2.36 – 2.18 (m, 2H), 2.09 – 1.95 (m, 1H), 1.95 – 1.84 (m, 1H), 1.77 (qd, *J* = 11.9, 4.1 Hz, 1H), 1.58 – 1.39 (m, 3H).

$^{13}$C NMR (101 MHz, $CDCl_3$) δ 157.7, 137.5, 129.1, 125.7, 123.0, 81.6, 64.1, 28.7, 28.4, 24.0, 23.7.

HRMS-ESI (pos): $[M + Na]^+$ calculated for $C_{13}H_{15}NO_2Na$ 240.0995, found 240.0997.

With general procedure using $Cs_2CO_3$ as a base and 24-hour reaction time the trans and cis products (retention and inversion respectively) were seen in GC-MS in ratio of about 2:1. This mixture was isolated and analyzed with NMR to confirm presence of both diastereomers and to help with assignment.

3-phenyl-1,3-oxazinan-2-one (**2o**)

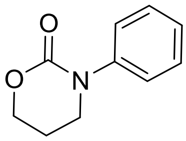


Synthetized according to the general procedure A with $Cs_2CO_3$ instead of t-BuOK. Reaction time 8 hours. Purified by flash column chromatography with EtOAc in hexane gradient. Yield 29.8 mg (17 %). Product is a white solid. Recorded NMR spectra matches the reported spectra.[7]

$^1$H NMR (400 MHz, $CDCl_3$) δ 7.42 – 7.20 (m, 5H), 4.43 – 4.34 (m, 2H), 3.69 (t, *J* = 6.1 Hz, 2H), 2.24 – 2.11 (m, 2H).

$^{13}$C NMR (101 MHz, $CDCl_3$) δ 152.8, 143.0, 129.2, 126.8, 125.9, 67.0, 48.8, 22.5.

(4S,5R)-4,5-diphenyloxazolidin-2-one (**2p**)

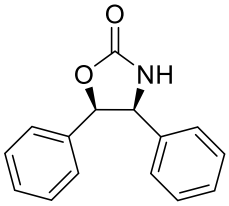

Synthetized according to the general procedure B. Reaction time 4 hours. Purified by filtering through silica. Yield 233.4 mg (98 %). Product is a white solid.

Also prepared at a 10 mmol scale in a 150 ml Schlenk flask. Purified as above. Yield 2.285 g (96 %).

Recorded NMR spectra matches the reported spectra[8]

$^1$H NMR (400 MHz, DMSO) δ 8.34 (s, 1H), 7.17 – 7.02 (m, 6H), 7.02 – 6.86 (m, 4H), 5.99 (dd, *J* = 8.4, 2.3 Hz, 1H), 5.28 (dd, *J* = 8.3, 2.3 Hz, 1H).

$^{13}$C NMR (101 MHz, DMSO) δ 158.8, 137.4, 135.6, 127.8, 127.6, 127.5, 127.4, 126.9, 126.1, 80.6, 59.9.

(S)-5-phenyloxazolidin-2-one (**2q)** and (R)-5-phenyloxazolidin-2-one (**2q')**

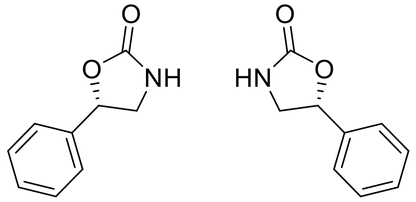


Synthetized according to the general procedure B. Reaction time 4 hours. Purified by flash column chromatography with EtOAc in hexane gradient. Yield 145.8 mg (89 %). Product is a white solid. Recorded NMR spectra matches the reported spectra.[9] Measured ee was 98.8 % for the product **2q** while starting material **1q** had ee > 99.9 %. The R isomer **2q'** was also prepared the same way**.** Yield 135.9 mg (83 %). Measured ee for **2q'** was 98.7 %, while starting material **1q'** had ee > 99.9 %.

$^1$H NMR (400 MHz, $CDCl_3$) δ 7.43 – 7.32 (m, 6H), 6.54 (s, 1H), 5.61 (t, *J* = 8.4 Hz, 1H), 3.98 (t, *J* = 8.9 Hz, 1H), 3.54 (t, *J* = 8.4 Hz, 1H).

$^{13}$C NMR (101 MHz, $CDCl_3$) δ 160.4, 138.5, 128.9, 128.9, 125.8, 78.0, 48.4.

4-benzyloxazolidin-2-one (**2r**)

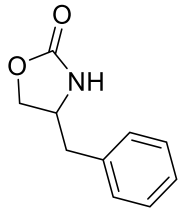

Synthetized according to the general procedure B. Reaction time 4 hours. Purified by flash column chromatography with EtOAc in hexane gradient. Yield 150.6 mg (85 %). Product is a white solid. Recorded NMR spectra matches the reported spectra.[10]

$^{1}$H NMR (400 MHz, $CDCl_3$) δ 7.41 – 7.17 (m, 5H), 6.30 (s, 1H), 4.41 (t, *J* = 7.8 Hz, 1H), 4.26 – 4.03 (m, 2H), 2.98 – 2.80 (m, 2H).

13C NMR (101 MHz, CDCl3) δ 159.8, 136.0, 129.1, 129.0, 127.2, 69.6, 53.8, 41.3.

4-((1H-indol-3-yl)methyl)oxazolidin-2-one (**2s**)

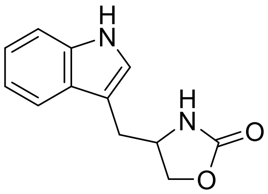

Synthetized according to the general procedure B. Reaction time 4 hours. Purified by flash column chromatography with EtOAc in hexane gradient. Yield 114.5 mg (53 %). Product is a light brown solid.

$^{1}$H NMR (400 MHz, $CDCl_3$) δ 8.33 (s, 1H), 7.55 (d, *J* = 7.9 Hz, 1H), 7.38 (dt, *J* = 8.5, 1.1 Hz, 1H), 7.22 (ddd, *J* = 8.2, 7.0, 1.2 Hz, 1H), 7.14 (ddd, *J* = 8.0, 7.0, 1.1 Hz, 1H), 7.03 (d, *J* = 2.5 Hz, 1H), 5.74 (s, 1H), 4.50 – 4.40 (m, 1H), 4.22 – 4.08 (m, 2H), 3.00 (d, *J* = 6.1 Hz, 2H).

$^{13}$C NMR (101 MHz, $CDCl_3$) δ 159.8, 136.5, 127.1, 122.9, 122.5, 119.9, 118.4, 111.7, 110.2, 70.1, 53.0, 31.4.

HRMS-ESI (pos): $[M + Na]^+$ calculated for $C_{12}H_{12}N_2O_2Na$ 239.0791, found 239.0780.

4-phenyl-1,3-oxazinan-2-one (**2t**)

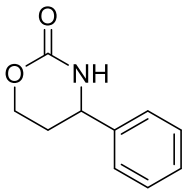

Synthetized according to the general procedure B. Reaction time 8 hours. Purified by flash column chromatography with EtOAc in hexane gradient. Yield 112.2 mg (60 %). Product is a white solid.

Recorded NMR spectra matches the reported spectra.[11]

$^1$H NMR (400 MHz, $CDCl_3$) δ 7.54 – 7.26 (m, 5H), 6.13 (s, 1H), 4.71 – 4.63 (m, 1H), 4.36 – 4.22 (m, 2H), 2.26 (dq, *J* = 14.2, 4.9 Hz, 1H), 2.04 – 1.92 (m, 1H).

$^{13}$C NMR (101 MHz, $CDCl_3$) δ 154.3, 141.2, 129.0, 128.3, 126.1, 64.9, 54.8, 30.4.

1,4-dihydro-2H-benzo[d][1,3]oxazin-2-one (**2u**)

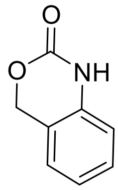


Synthetized according to the general procedure B. Reaction time 8 hours. Purified by flash column chromatography with EtOAc in hexane gradient. Yield 36.9 mg (25 %). Product is a white solid. Recorded NMR spectra matches the reported spectra.[12]

$^1$H NMR (400 MHz, $CDCl_3$) δ 8.72 (s, 1H), 7.32 – 7.22 (m, 1H), 7.14 – 7.01 (m, 2H), 6.87 (dd, *J* = 7.9, 1.0 Hz, 1H), 5.33 (s, 2H).

$^{13}$C NMR (101 MHz, $CDCl_3$) δ 153.8, 135.7, 129.4, 124.3, 123.5, 114.3, 68.8.

3-butyl-4,4-dimethyloxazolidin-2-one (**2v**)

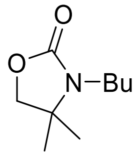


Prepared from the corresponding N-H amino alcohol in a scale of 5 mmol using 20 mmol of n-BuCl, 12 mmol of t-BuOK and 3 ml of DMSO (Scheme S1). Reaction time 16 hours. Isolation was done as with other oxazolidinones. Yield 453.8 mg (53 %). Product is a dark brown oil.

$^1$H NMR (400 MHz, $CDCl_3$) δ 3.91 (s, 2H), 3.08 – 3.00 (m, 2H), 1.59 – 1.46 (m, 2H), 1.35 – 1.25 (m, 2H), 1.24 (s, 6H), 0.88 (t, *J* = 7.3 Hz, 3H).

$^{13}$C NMR (101 MHz, $CDCl_3$) δ 157.8, 74.8, 58.4, 40.2, 31.8, 25.2, 20.2, 13.8.

HRMS-ESI (pos): $[M + Na]^+$ calculated for $C_9H_{17}NO_2Na$ 194.1151, found 194.1149.

# 5 SYNTHESIS OF AMINO ALCOHOLS

## 5.1 GENERAL PROCEDURES

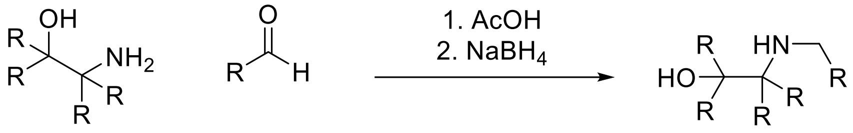


*Scheme S2. General procedure for preparation of substituted amino alcohols.*

The non-commercial amino alcohols were mostly prepared by reductive amination using one of the two procedures below.

**General procedure C**

10 mmol aldehyde, 1.05 equiv aminoalcohol, 10 mol % acetic acid and 60 ml dry MeOH were added to a 100 ml flask. About 1.5 g of 3 Å molecular sieves were added and the flask was swirled with slow speed overnight. The completion of the imine formation was checked with TLC. Then $NaBH_4$ (1.1 equiv) was added slowly under stirring. After bubbling had seized, the reaction was mixed for further 2 hours. The mixture was filtered to remove the sieves, the filtrate was transferred to a separatory funnel along with 200 ml of water and was extracted with 3x 50 ml of DCM. The organic phase was washed with conc. NaCl, dried with $Na_2SO_4$, filtered and evaporated. The crude product was purified as separately specified.

**General procedure D**

10 mmol aldehyde (unless otherwise noted), 1.05 equiv aminoalcohol, 10 mol % acetic acid and 20 ml toluene were added to a 50 ml flask. The imine was allowed to form by removing water with a Dean-Stark apparatus, until no aldehyde was detected on TLC. The solution was allowed to cool down to room temperature and diluted to half concentration with anhydrous

methanol. Then $NaBH_4$ (1.1 equiv) was added slowly under stirring. After bubbling had seized, the reaction was mixed for further 2 hours. The mixture was transferred to a separatory funnel along with 200 ml of water and was extracted with 3x 50 ml of DCM. The organic phase was washed with conc. NaCl, dried with $Na_2SO_4$, filtered and evaporated. The crude product was purified as separately specified.

## 5.2 SYNTHESIS

1-((4-(benzyloxy)benzyl)amino)propan-2-ol (**1f**)

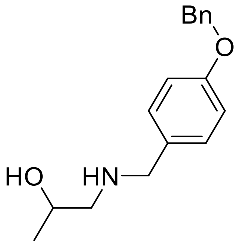


Product was prepared by general procedure D. Crude product was purified by recrystallization from EtOH:hex mixture using elevated temperature. Yield 2.00 g (74 %). Product is a white solid.

$^1$H NMR (400 MHz, $CDCl_3$) δ 7.47 – 7.28 (m, 5H), 7.28 – 7.17 (m, 2H), 7.00 – 6.90 (m, 2H), 5.06 (s, 2H), 3.83 – 3.67 (m, 3H), 2.73 (dd, *J* = 12.0, 3.1 Hz, 1H), 2.42 (dd, *J* = 12.0, 9.4 Hz, 1H), 1.14 (d, *J* = 6.2 Hz, 3H).

$^{13}$C NMR (101 MHz, $CDCl_3$) δ 158.1, 137.2, 132.7, 129.4, 128.7, 128.1, 127.6, 115.0, 70.2, 65.8, 56.3, 53.2, 20.5.

HRMS-ESI (pos): $[M + K]^+$ calculated for $C_{17}H_{21}NO_2K$ 310.1204, found 310.1193.

1-((4-(trifluoromethyl)benzyl)amino)propan-2-ol (**1g**)

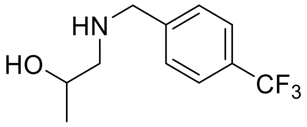


Product was prepared by general procedure D. Scale was 15 mmol. Crude product was purified by recrystallization from EtOH:water mixture using elevated temperature. Yield 731 mg (21 %). Product is a white solid.

$^{1}$H NMR (400 MHz, DMSO) δ 7.69 – 7.62 (m, 2H), 7.59 – 7.51 (m, 2H), 4.46 (d, *J* = 4.4 Hz, 1H), 3.79 (s, 2H), 3.69 (qd, *J* = 6.1, 3.9 Hz, 1H), 2.39 (d, *J* = 6.0 Hz, 2H), 2.16 (s, 1H), 1.03 (d, *J* = 6.2 Hz, 3H).

$^{13}$C NMR (101 MHz, DMSO) δ 146.2, 128.5, 127.2 (q, *J* = 31.6 Hz), 124.9 (q, *J* = 3.8 Hz), 124.4 (d, *J* = 271.7 Hz), 65.3, 56.6, 52.4, 21.5.

HRMS-ESI (pos): $[M + Na]^+$ calculated for $C_{11}H_{14}F_3NONa$ 256.0920, found 256.0928.

1-((3,7-dimethyloct-6-en-1-yl)amino)propan-2-ol (**1h**)

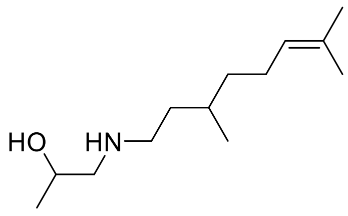


Product was prepared by general procedure D. Crude product was purified by vacuum distillation. Yield 898 mg (42 %). Product is a colorless oil. The diastereomers were not separated by distillation so this is a diastereomeric mixture.

$^{1}$H NMR (400 MHz, $CDCl_3$) δ 5.09 (tp, *J* = 7.1, 1.4 Hz, 1H), 3.74 (dqd, *J* = 9.3, 6.2, 3.1 Hz, 1H), 2.76 – 2.50 (m, 3H), 2.38 (ddd, *J* = 12.1, 9.5, 2.8 Hz, 1H), 2.07 – 1.87 (m, 2H), 1.68 (s, 2H), 1.60 (s, 3H), 1.50 (ttd, *J* = 12.6, 5.9, 2.8 Hz, 2H), 1.30 (dddd, *J* = 13.6, 9.4, 7.6, 4.1 Hz, 2H), 1.22 – 1.08 (m, 4H), 0.88 (d, *J* = 6.4 Hz, 3H).

$^{13}$C NMR (101 MHz, $CDCl_3$) δ 131.4, 124.9, 65.6 (d, *J* = 5.4 Hz), 57.1 (d, *J* = 4.1 Hz), 47.7 (d, *J* = 6.1 Hz), 37.6 (d, *J* = 2.0 Hz), 37.3, 30.6, 25.9, 25.6, 20.6, 19.8, 17.8.

HRMS-ESI (pos): $[M + Na]^+$ calculated for $C_{13}H_{27}NONa$ 236.1985, found 236.1979.

2-(((1-methyl-1H-indol-3-yl)methyl)amino)-1-phenylethan-1-ol (**1i**)

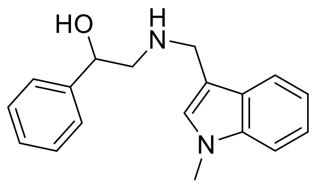

Product was prepared by general procedure C in 10 mmol scale. Crude product was recrystallized from EtOH:water using elevated temperature. Yield 2.327 g (83 %). Product is a brown solid.

$^1$H NMR (400 MHz, $CDCl_3$) δ 7.58 (dt, *J* = 7.9, 1.0 Hz, 1H), 7.37 (dt, *J* = 8.2, 0.9 Hz, 1H), 7.35 – 7.26 (m, 4H), 7.24 – 7.19 (m, 1H), 7.18 (s, 1H), 7.13 (ddd, *J* = 8.2, 7.0, 1.2 Hz, 1H), 7.00 (ddd, *J* = 7.9, 7.0, 1.0 Hz, 1H), 5.25 (s, 1H), 4.67 (dd, *J* = 7.6, 4.9 Hz, 1H), 3.94 – 3.78 (m, 2H), 3.73 (s, 3H), 2.75 – 2.61 (m, 2H).

$^{13}$C NMR (101 MHz, $CDCl_3$) δ 144.7, 136.7, 127.9, 127.8, 127.3, 126.7, 125.9, 121.0, 118.9, 118.4, 113.0, 109.5, 71.4, 57.2, 43.9, 32.2.

HRMS-ESI (pos): $[M + Na]^+$ calculated for $C_{18}H_{20}N_2ONa$ 303.1468, found 303.1462.

1-((3-bromobenzyl)amino)propan-2-ol (**1j**)

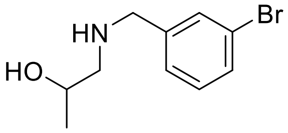


Product was prepared by general procedure D. Scale was 15 mmol. Crude product was purified by vacuum distillation. Yield 3.02 g (82 %). Product is a yellow tint oil.

$^1$H NMR (400 MHz, $CDCl_3$) δ 7.47 (t, *J* = 1.9 Hz, 1H), 7.39 (dt, *J* = 7.6, 1.7 Hz, 1H), 7.26 – 7.15 (m, 2H), 3.89 – 3.71 (m, 3H), 2.72 (dd, *J* = 12.0, 3.1 Hz, 1H), 2.43 (dd, *J* = 12.0, 9.3 Hz, 1H), 1.15 (d, *J* = 6.2 Hz, 3H).

$^{13}$C NMR (101 MHz, $CDCl_3$) δ 142.7, 131.2, 130.3, 130.2, 126.8, 122.7, 65.9, 56.4, 53.2, 20.6.

HRMS-ESI (pos): $[M + Na]^+$ calculated for $C_{10}H_{14}BrNONa$ 266.0151, found 266.0151.

(S)-2-amino-3-(1H-indol-3-yl)propan-1-ol (L-tryptophanol) (**1s**)

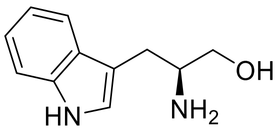


Into a 250 ml round bottomed Schlenk flask was added L-tryptophan (3.06 g, 15 mmol) and 150 ml of THF. The flask was flushed with argon at low flow rate. $LiAlH_4$ (2.28 g, 60 mmol,

4 equiv.) was added in small portions. The mixture was mixed at room temperature for 10 minutes. A condenser equipped with a balloon was placed on top of the flask and the balloon was allowed to fill. The valve of the flask was then closed. The contents were refluxed for 12 hours. The mixture was allowed to cool to room temperature and then carefully quenched with 20 ml water and 10 ml of 5 M NaOH. The suspension was filtered. THF was evaporated and the remaining filtrate was extracted with 2 x 50 ml of EtOAc. The combined organic phase was washed with 30 ml of conc. NaCl solution and dried with $Na_2SO_4$. After removing the solvent, the crude orange pasty product was vigorously dried by heating under reduced pressure (Some solvent residues are still seen in NMR.). This afforded the product as a hygroscopic orange solid (2.673 g, 94 %). This product was used for carbamate formation without further purification. Recorded NMR spectra matches the reported spectra.[13]

$^1$H NMR (400 MHz, DMSO) δ 10.86 (s, 1H), 7.56 (dd, *J* = 7.9, 2.4 Hz, 1H), 7.36 (dd, *J* = 8.1, 2.5 Hz, 1H), 7.15 (s, 1H), 7.07 (ddd, *J* = 10.2, 6.4, 2.1 Hz, 1H), 6.98 (td, *J* = 7.3, 2.2 Hz, 1H), 3.38 (ddd, *J* = 10.6, 4.8, 2.5 Hz, 1H), 3.25 (ddd, *J* = 9.7, 6.7, 2.5 Hz, 1H), 3.00 (td, *J* = 7.0, 3.7 Hz, 1H), 2.81 (ddd, *J* = 14.2, 5.9, 2.5 Hz, 1H), 2.59 (ddd, *J* = 14.3, 7.3, 2.5 Hz, 1H).

$^{13}$C NMR (101 MHz, DMSO) δ 136.3, 127.7, 123.3, 120.8, 118.5, 118.1, 111.8, 111.3, 66.2, 53.6, 29.8.

4-(((2-hydroxypropyl)amino)methyl)benzonitrile (**1w**)

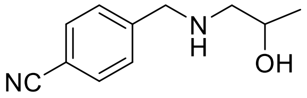


Product was prepared by general procedure D. Crude product was purified by recrystallization from EtOH:water mixture using elevated temperature. Yield 799 mg (42 %). Product is a white solid

$^1$H NMR (400 MHz, $CDCl_3$) δ 7.66 – 7.59 (m, 2H), 7.49 – 7.41 (m, 2H), 3.88 (d, *J* = 6.7 Hz, 2H), 3.81 (ddd, *J* = 12.5, 6.2, 3.1 Hz, 1H), 3.72 (q, *J* = 7.0 Hz, 1H), 2.72 (dd, *J* = 12.0, 3.0 Hz, 1H), 2.45 (dd, *J* = 12.0, 9.2 Hz, 1H), 1.24 (t, *J* = 7.0 Hz, 1H), 1.16 (d, *J* = 6.2 Hz, 3H).

$^{13}$C NMR (101 MHz, $CDCl_3$) δ 145.87, 132.44, 128.73, 119.02, 111.08, 66.09, 56.52, 53.36, 20.65.

HRMS-ESI (pos): $[M + H]^+$ calculated for $C_{11}H_{15}N_2O$ 191.1179, found 191.1185.

methyl 4-(((2-hydroxypropyl)amino)methyl)benzoate (**1x**)

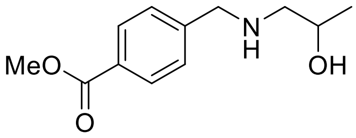


Product was prepared by general procedure D. Crude product was purified by recrystallization from EtOH:heptane mixture using elevated temperature. Yield 1.08 g (42 %). Product is a white solid.

$^{1}$H NMR (400 MHz, $CDCl_3$) δ 8.04 – 7.97 (m, 2H), 7.42 – 7.36 (m, 2H), 3.91 (s, 3H), 3.87 (d, *J* = 6.8 Hz, 2H), 3.84 – 3.75 (m, 1H), 2.73 (dd, *J* = 12.0, 3.1 Hz, 1H), 2.44 (dd, *J* = 12.0, 9.3 Hz, 1H), 1.15 (d, *J* = 6.2 Hz, 3H).

$^{13}$C NMR (101 MHz, $CDCl_3$) δ 167.1, 145.6, 130.0, 129.1, 128.0, 65.9, 56.5, 53.5, 52.2, 20.6.

HRMS-ESI (pos): $[M + Na]^+$ calculated for $C_{12}H_{17}NO_3$ 246.1101, found 246.1097.

1-((pyridin-2-ylmethyl)amino)propan-2-ol **(1y**)

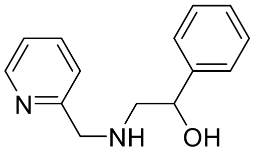


Product was prepared by general procedure D. Crude product was purified by vacuum distillation. Yield 571 mg (25 %). Product is a yellow-brown oil.

$^{1}$H NMR (400 MHz, DMSO) δ 8.48 (ddd, *J* = 4.9, 1.8, 0.9 Hz, 1H), 7.72 (td, *J* = 7.6, 1.9 Hz, 1H), 7.41 – 7.17 (m, 7H), 5.34 (s, 1H), 4.67 (t, *J* = 6.2 Hz, 1H), 3.89 – 3.76 (m, 2H), 2.67 (d, *J* = 6.2 Hz, 2H).

$^{13}$C NMR (101 MHz, DMSO) δ 160.2, 148.8, 144.6, 136.4, 127.9, 126.8, 125.9, 121.8, 121.8, 71.7, 57.3, 54.4.

HRMS-ESI (pos): $[M + H]^+$ calculated for $C_{14}H_{17}N_2O$ 229.1335, found 229.1346.

# 6 OTHER SYNTHESIS

butyl ((1S,2S)-1-hydroxy-1-phenylpropan-2-yl)(methyl)carbamate (**4a**)

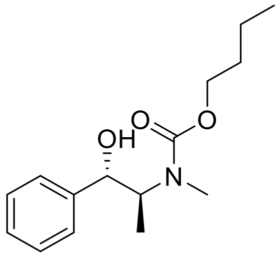


Product was prepared according to modified general procedure A. Instead of heating for 4 hours at 140 °C, the synthesis was done over 12 hours at 80 °C. Isolation was done according to the general procedure. The crude product was purified by silica column chromatography. Yield 140.2 mg (53 %). Product is a clear oil.

$^{1}$H NMR (400 MHz, $CDCl_3$) δ 7.36 – 7.32 (m, 4H), 7.30 – 7.27 (m, 1H), 4.56 (d, *J* = 8.4 Hz, 1H), 4.32 – 4.16 (m, 1H), 4.09 (td, *J* = 6.6, 3.1 Hz, 2H), 2.78 (s, 3H), 1.68 – 1.56 (m, 2H), 1.39 (h, *J* = 7.4 Hz, 2H), 1.03 (d, *J* = 7.1 Hz, 3H), 0.94 (t, *J* = 7.4 Hz, 3H).

$^{13}$C NMR (101 MHz, $CDCl_3$) δ (158.0, 157.3 rotamers), (142.2, 141.8 rotamers), 128.3, 127.7, 126.7, (76.1, 75.8 rotamers), 65.4, (58.3,57.1 rotamers), 31.0, 30.1, 19.1, 14.5, 13.7.

HRMS-ESI (pos): $[M + Na]^+$ calculated for $C_{15}H_{23}NO_3$ 288.1570, found 288.1568.

(4S,5S)-3,4-dimethyl-5-phenyloxazolidin-2-one (**2a**) and (4R,5S)-3,4-dimethyl-5-phenyloxazolidin-2-one (**2a'**) independently independent synthesis.

To make the calibration curves and to confidently show that our procedure proceeds with enantio-retention, **2a (**(4S,5S)-3,4-dimethyl-5-phenyloxazolidin-2-one) and **2a'** ((4R,5S)-3,4-dimethyl-5-phenyloxazolidin-2-one) were prepared from **1a** ((+)-pseudoephedrine) via independent methods.

(4S,5S)-3,4-dimethyl-5-phenyloxazolidin-2-one with CDI

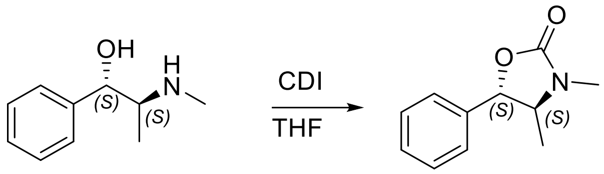


*Scheme S3. Independent preparation of **2a**.*

Into a 50 ml flask was placed dry THF (20 ml), (1S,2S)-2-2methylamino-1-phenylpropan-1-ol (330 mg, 2 mmol), 1.1'-Carbonyldiimidazole (332 mg, 1.05 equiv) and DBU (298.6 µl, 2 equiv). The solution was then refluxed until no staring material was seen on TLC. The cooled solution was placed in a separatory funnel along with 50 ml of water. The contents were then extracted with 2 x 20 ml of EtOAc, dried with $Na_2SO_4$ and solvent was evaporated to afford 324 mg (85 %) of an oily product.

The NMR matches the spectra of the product prepared by the general procedure A.

$^1$H NMR (400 MHz, $CDCl_3$) δ 7.45 – 7.30 (m, 5H), 4.90 (d, *J* = 7.7 Hz, 1H), 3.54 (dq, *J* = 7.8, 6.1 Hz, 1H), 2.86 (s, 3H), 1.36 (d, *J* = 6.2 Hz, 3H).

Preparation of (4R,5S)-3,4-dimethyl-5-phenyloxazolidin-2-one according to a modified literature procedure.[14]

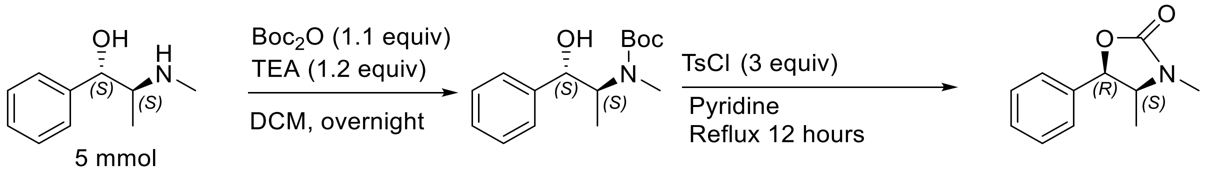


*Scheme S4. Preparation of **2a'**.*

Into a 50 ml flask were added DCM (30 ml), (+)-pseudoephedrine (5 mmol), TEA (1.2 equiv, 6 mmol) and $Boc_2O$ (1.1 equiv, 5.5 mmol). The solution was stirred for 12 hours. The Boc protected amino alcohol was isolated by transferring the content of the flask to a separatory funnel along with 100 ml of water and 30 ml of DCM, and extracting once. The organic layer was washed with conc. NaCl solution and then dried with $Na_2SO_4$. The crude product was used in the next step without further purification.

Into a 50 ml flask were added pyridine (13 ml), the crude Boc protected amino alcohol and TsCl (3 equiv, 15 mmol). The solution was refluxed overnight (12 hours). The oxazolidinone was isolated by pouring the mixture into 100 ml of water and then extracting with 50 ml of

EtOAc. The organic layer was washed with 50 ml of water and 50 ml of conc. NaCl solution, dried with $Na_2SO_4$ and evaporated. The crude product was purified with column chromatography using 15 % to 50 % gradient of EtOAc in hex. After this, the product still contained small amount of the corresponding S,S-oxazolidinone so it was recrystallized from DCM:hex to afford transparent crystals of the single diastereomer in overall yield of 325.3 mg (34 %). Recorded NMR spectra matches the reported spectra.[15]

$^1$H NMR (400 MHz, $CDCl_3$) δ 7.43 – 7.29 (m, 3H), 7.30 – 7.23 (m, 2H), 5.58 (d, *J* = 8.2 Hz, 1H), 4.02 (dq, *J* = 8.2, 6.6 Hz, 1H), 2.88 (s, 3H), 0.78 (d, *J* = 6.6 Hz, 3H).

$^{13}$C NMR (101 MHz, $CDCl_3$) δ 158.1, 135.2, 128.52, 128.49, 126.2, 78.4, 57.1, 29.0, 14.3.

# 7 FAILED SYNTHESIS

Despite our best efforts some amino alcohols were either too unreactive to cyclize or gave very poor selectivity towards the desired cyclic carbamate.

4-((2-oxo-5-phenyloxazolidin-3-yl)methyl)benzonitrile (**2w**)

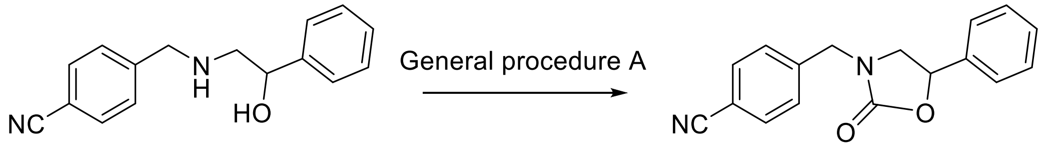


**1w** **2w,** Not detected

*Scheme S 5. Attempted preparation of* ***2w****.*

With general procedure A. Reaction time 4 hours. Forms a complex mixture. No MS peak corresponding to the product **2w** was seen in GC-MS.

methyl 4-((2-oxo-5-phenyloxazolidin-3-yl)methyl)benzoate (**2x**)

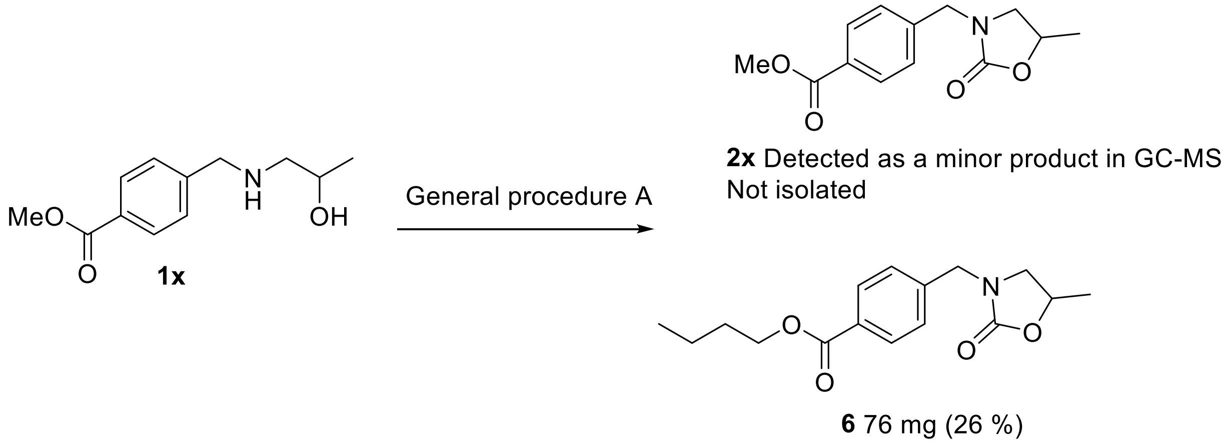


*Scheme S6. Attempted preparation of* ***2x****.*

With general procedure A. Reaction time 24 hours. Starting from the methyl ester amino alcohol **1x**, the methyl ester oxazolidine **2x** was seen in GC-MS as only a very minor product and could not be isolated. Instead, the corresponding butyl ester **6** was isolated in 26 % yield.

butyl 4-((5-methyl-2-oxooxazolidin-3-yl)methyl)benzoate (**6**)

$^{1}$H NMR (400 MHz, $CDCl_3$) δ 8.08 – 7.98 (m, 2H), 7.34 (dt, $J$ = 7.9, 1.2 Hz, 2H), 4.70 – 4.57 (m, 1H), 4.53 – 4.40 (m, 2H), 4.32 (t, $J$ = 6.6 Hz, 2H), 3.56 – 3.46 (m, 1H), 3.03 – 2.93 (m, 1H), 1.74 (ddt, $J$ = 8.8, 7.8, 6.5 Hz, 2H), 1.54 – 1.45 (m, 1H), 1.48 – 1.39 (m, 1H), 1.39 (d, $J$ = 6.2 Hz, 3H), 0.97 (t, $J$ = 7.4 Hz, 3H)

$^{13}$C NMR (101 MHz, $CDCl_3$) δ 166.3, 158.2, 140.9, 130.3, 130.1, 127.9, 70.2, 65.0, 50.8, 48.0, 30.8, 20.7, 19.3, 13.8.

HRMS-ESI (pos): $[M + H]^+$ calculated for $C_{16}H_{22}NO_4$ 292.1543, found 292.1531

(S)-tetrahydro-1H,3H-pyrrolo[1,2-c]oxazol-3-one (**2x**)

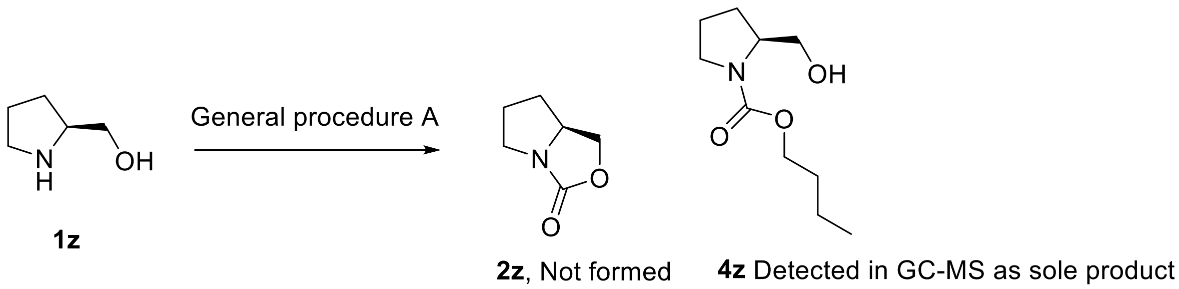


*Scheme S7. Attempted preparation of* ***2z****.*

With general procedure A. Reaction time 24 hours. No oxazolidinone product was isolated but only roughly quantitative amount of the corresponding butyl carbamate intermediate (**3z**). The reaction was repeated at 180 °C temperature and reaction time of 24 hours. Again, only butyl carbamate **4z** was detected.

5-phenyl-3-(pyridin-2-ylmethyl)oxazolidin-2-one (**2y**)

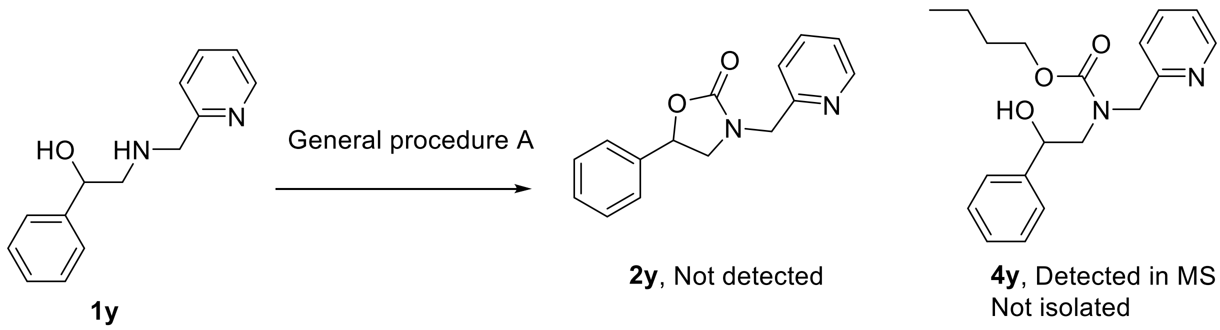


*Scheme S8. Attempted preparation of* ***2y****.*

With general procedure A. Reaction time 8 hours. Only the compound with a mass corresponding to the butyl carbamate species **4y** was detected in HRMS-ESI of the crude mixture. This was not isolated. Performing the reaction at 180 °C for 24 hours also did not yield the desired oxazolidinone.

3-benzyl-5,5-dimethyloxazolidin-2-one (**2aa**)

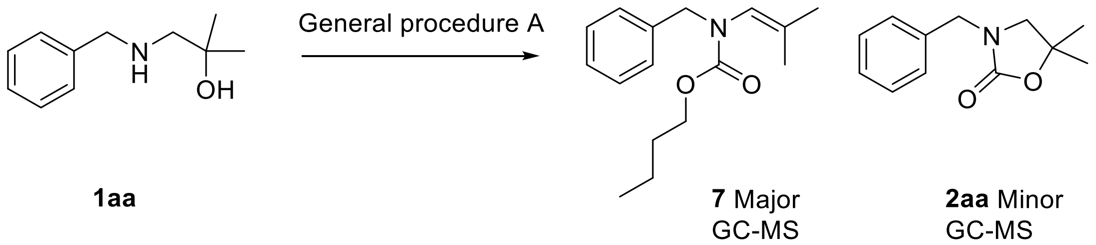


*Scheme S9. Attempted preparation of* ***2aa****.*

With general procedure A. Reaction time 4 hours. The mass peak corresponding to the elimination products **7** was detected along with much smaller peak corresponding to the desired product **2aa**. **2aa** was not isolated due to the poor selectivity.

1,3-oxazinan-2-one (**2bb**)

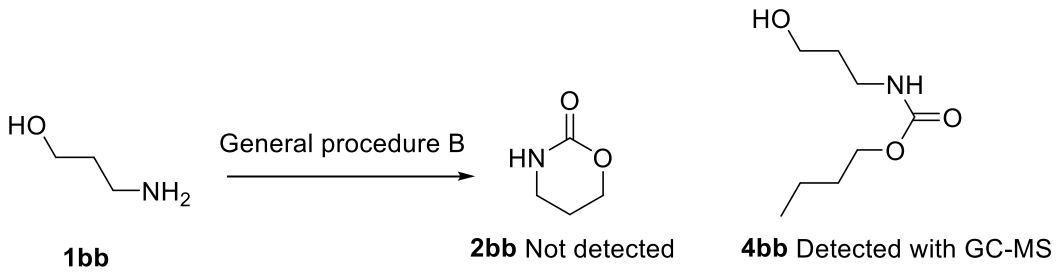


*Scheme S10. Attempted preparation of* ***2bb****.*

With general procedure B. Reaction time 24 hours Only compound with GC-MS peak corresponding to the butyl carbamate species **4bb** was detected. No cyclization product was observed. Continuing the reaction for further 24 hours did not reduce the amount of the butyl carbamate.

# 8 CHIRAL CHROMATOGRAPHY

To determine the ee of **2q** ((S)-5-phenyloxazolidin-2-one) and **2q’** ((R)-5-phenyloxazolidin-2-one) as well as the commercial amino alcohols **1q** and **1q’** chiral chromatography analysis was performed. Table S3 lists the details of the chromatography system. Table S4 list the conditions used for separation of **2q** and **2q’**. Figure S2 shows the chromatograms of **2q’** (R) and **2q** (S) giving ee values of 98.7 % and 98.8 % respectively.

*Table S3. Instrument used for the chiral chromatography experiments.*

| **Instrument Modules of the Agilent 1260 Infinity Series HPLC System** | |
|---|---|
| **Degasser** | G1322A, Serial No: JPAAL86774 |
| **Binary Pump** | G1312B, Serial No: DEACB10897 |
| **Auto Sampler** | G1329B, Serial No: DEAAC43050 |
| **Column Department** | G1316A |
| **Diode Array Detector** | G1315B, Serial No: DE11113796 |
| **Control Software** | ChemStation for LC systems, Rev.B.01.03-SR2[204] |

*Table S4. Conditions for the separation of **2q** and **2q'**.*

| | |
|---|---|
| **HPLC Method** | |
| **HPLC Instrument** | Agilent 1260 Infinity Series |
| **Column** | Chiralpak IC (5 mm) 100 × 4.6 mm i.d. |
| **Mobile Phase** | Mobile Phase A H2O:ACN:FA 1000:50:0.5 (v/v)<br>Mobile Phase B H2O:ACN:FA 50:1000:0.5 (v/v)<br>Isocratic: MP A : MP B = 80 : 20 (v/v) |
| **Column Temperature (C)** | 25 C |
| **Flow Rate (mL/min)** | 1.0 |
| **Sample Conc (mg/mL)** | 1.0 (Acetonitrile) |
| **Injected Volume (uL)** | 1.0 |
| **UV Detection** | 210 nm, Ref = 480 nm |

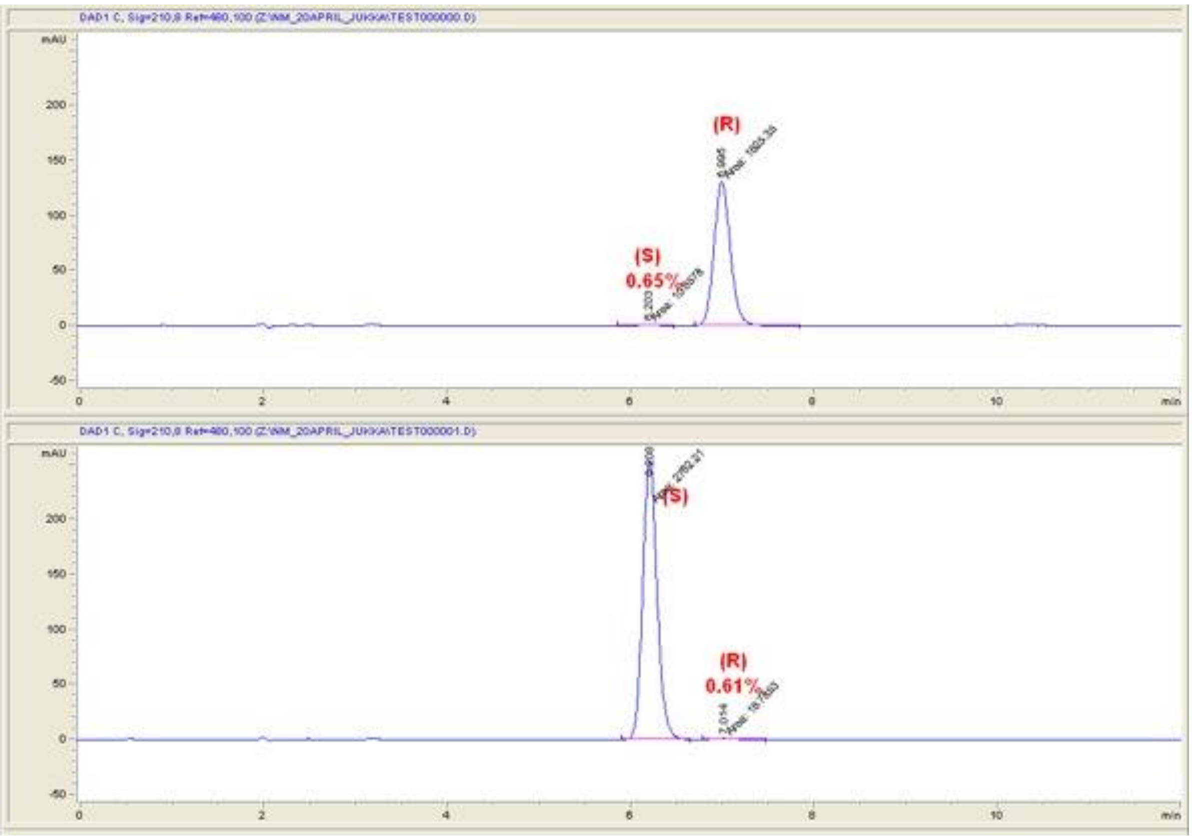


*Figure S2. Chromatograms of **2q'** (R) and **2q** (S) showing the relative amount of both enantiomers.*

Next the starting compounds **1q** and **1q'** were analyzed. To separate these enantiomers, derivatization to the N,O-diacetyl compounds was necessary. The derivatization was done according to the literature.[16] The derivatized compounds were further purified by RP-HPLC before analyzing with chiral chromatography. Table S5 lists the conditions used for separation of the derivatized **1q** and **1q'**. Figure S3 shows the chromatograms of the derivatized amino alcohols **1q'** (R) and **1q** (S). The ee values are > 99.9 % for both.

*Table S5. Conditions for separation of O,N-diacetylated amino alcohols **1q** and **1q'**.*

| **HPLC Method** | |
|---|---|
| **HPLC Instrument** | Agilent 1260 Infinity Series |
| **Column** | Reprosil Chiral-NR (8 mm) 150 × 4.6 mm i.d. |
| **Mobile Phase** | Mobile Phase A H2O:ACN:FA 1000:50:0.5 (v/v)<br>Mobile Phase B H2O:ACN:FA 50:1000:0.5 (v/v)<br>Isocratic: MP A : MP B = 80:20 |
| **Column Temperature (C)** | 25 C |
| **Flow Rate (mL/min)** | 1.0 |
| **Sample Conc (mg/mL)** | 1.0 (Acetonitrile) |
| **Injected Volume (uL)** | 1.0 |
| **UV Detection** | 210 nm, Ref = 480 nm |

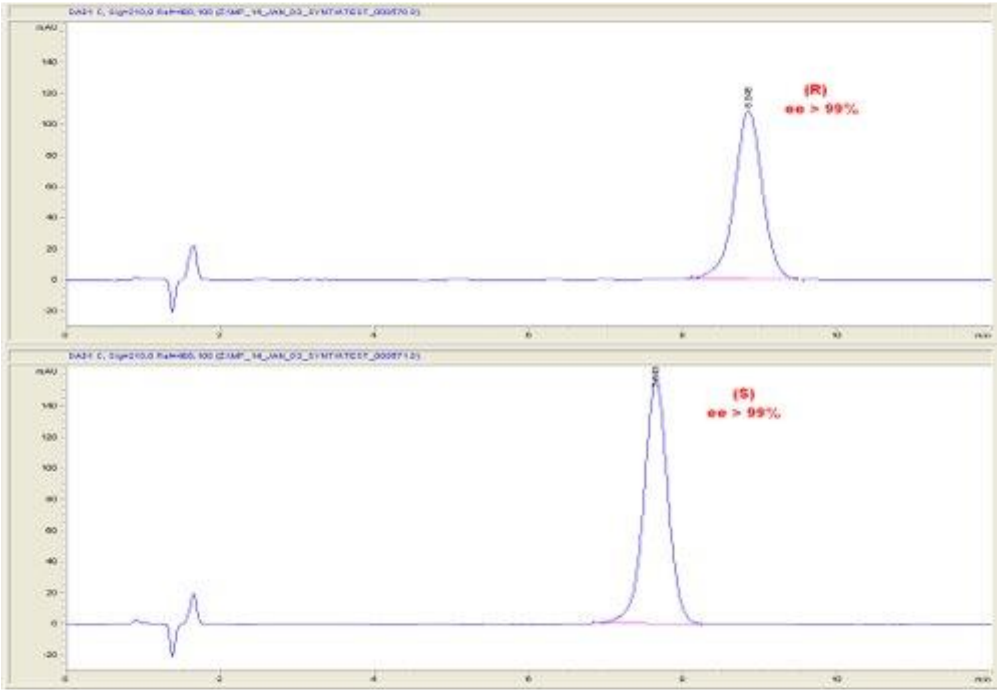


*Figure S3. Chromatograms of O,N-diacetylated **1q'** (R) and **1q** (S).*

# 9 NMR SPECTRA

## 9.1 CARBAMATES

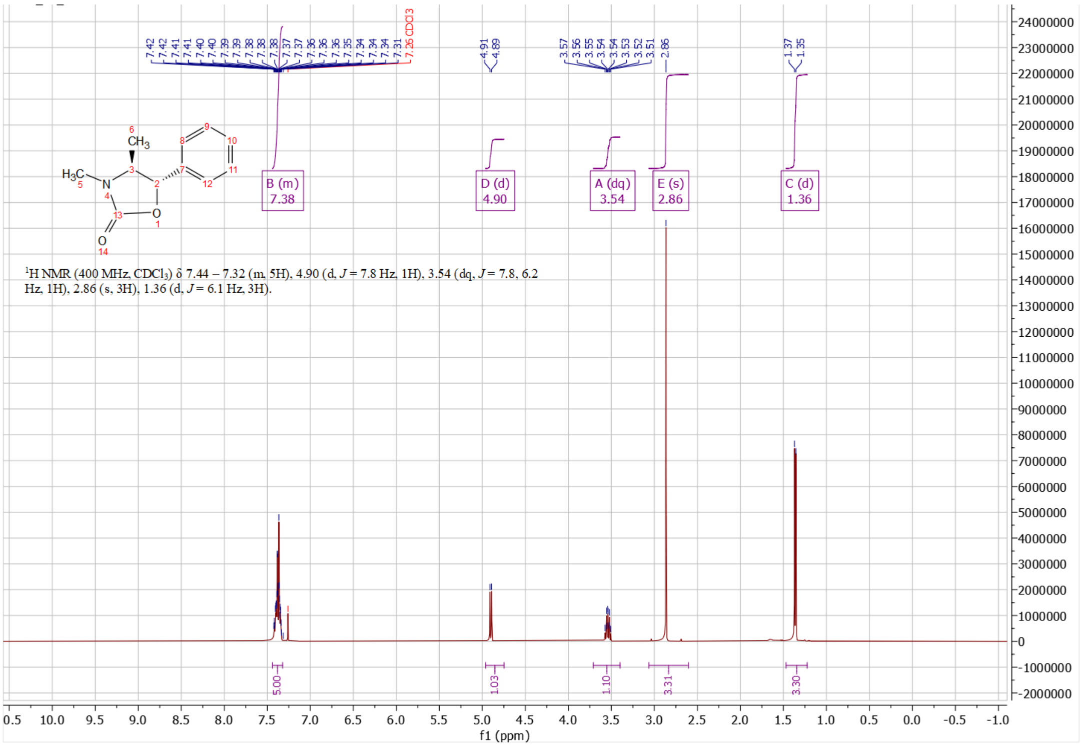


*Figure S4. $^1$H NMR spectra of **2a**.*

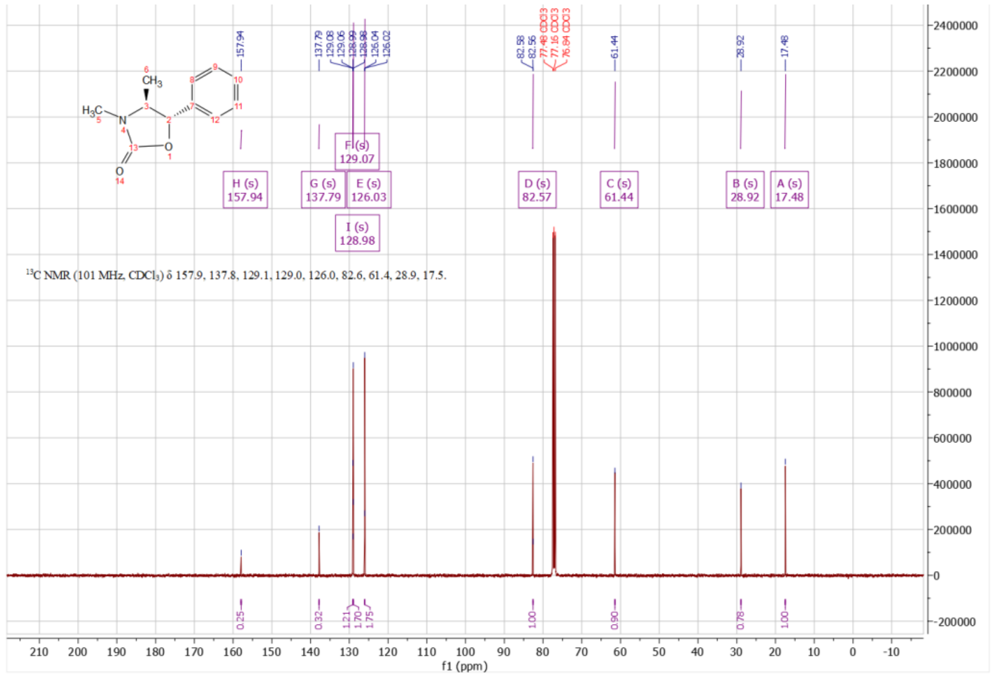


*Figure S5. $^{13}$C NMR spectra of **2a**.*

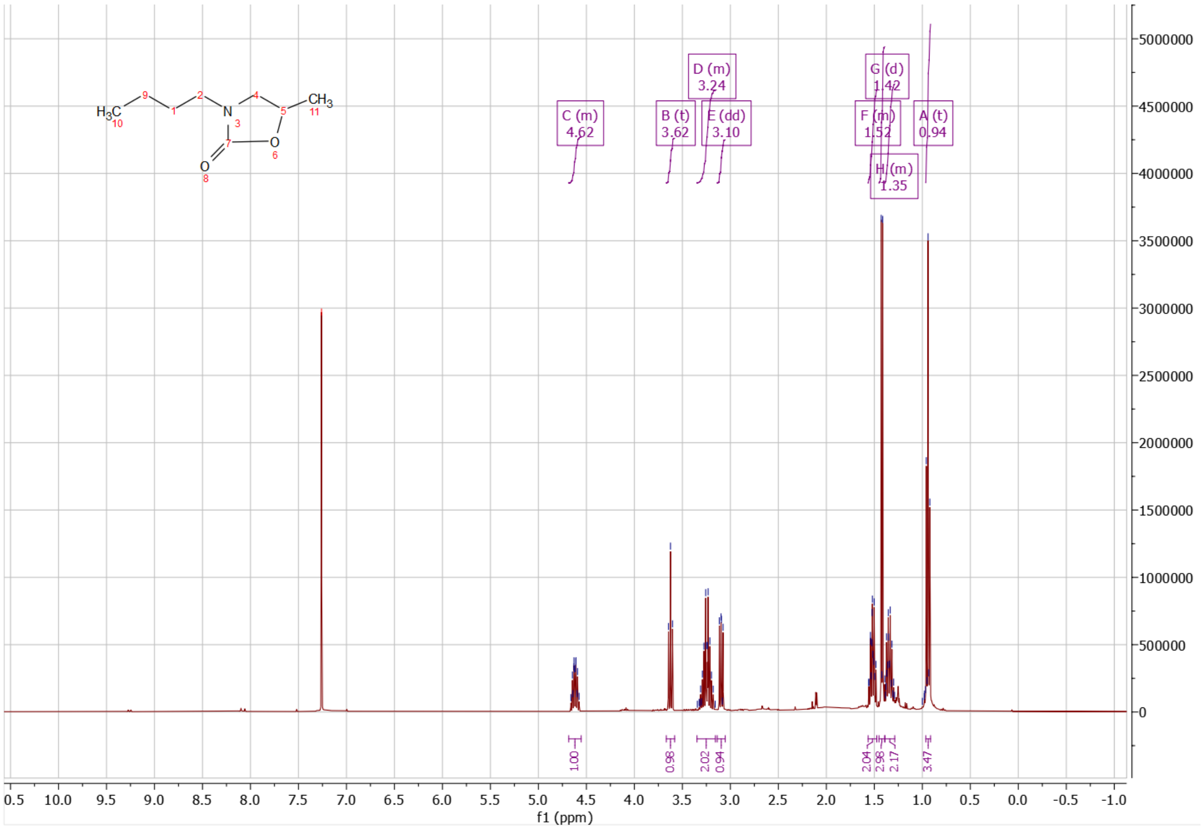

*Figure S6. $^{1}$H NMR spoectra of **2b**.*

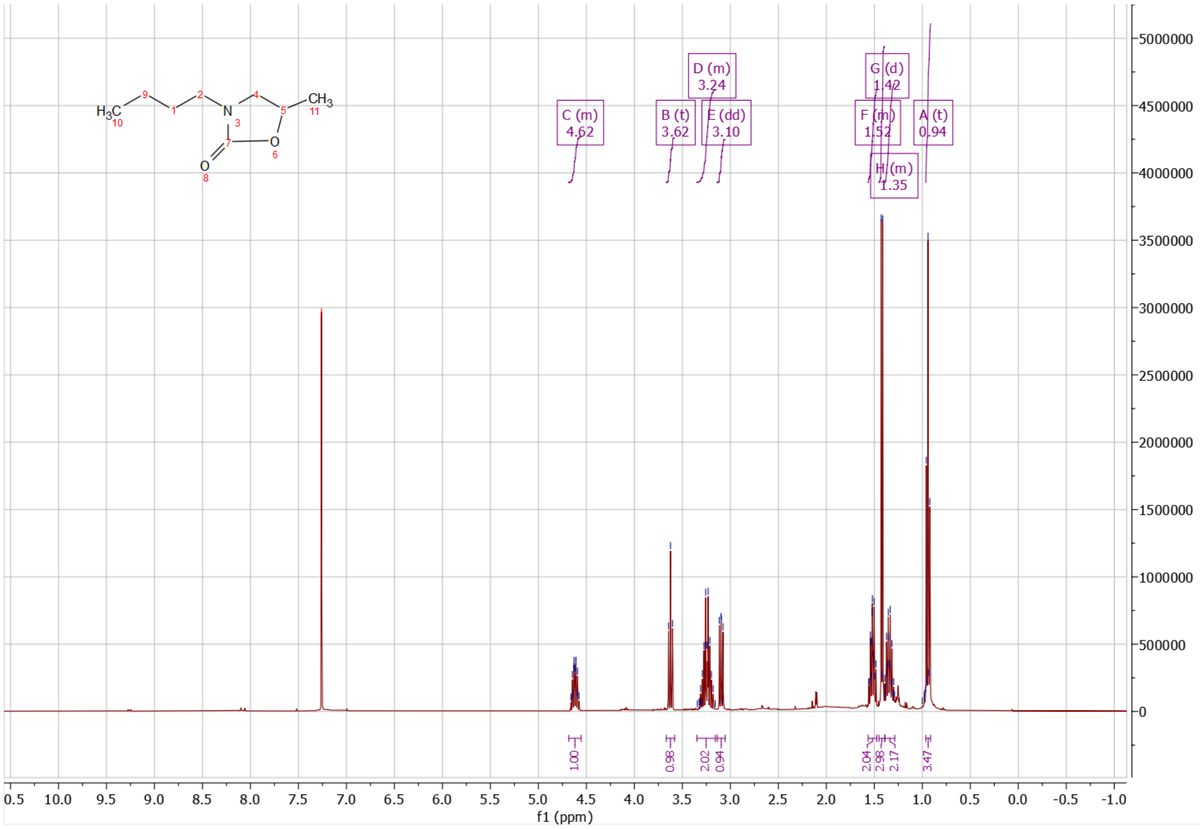

*Figure S7. $^{13}$C NMR spectra of **2b**.*

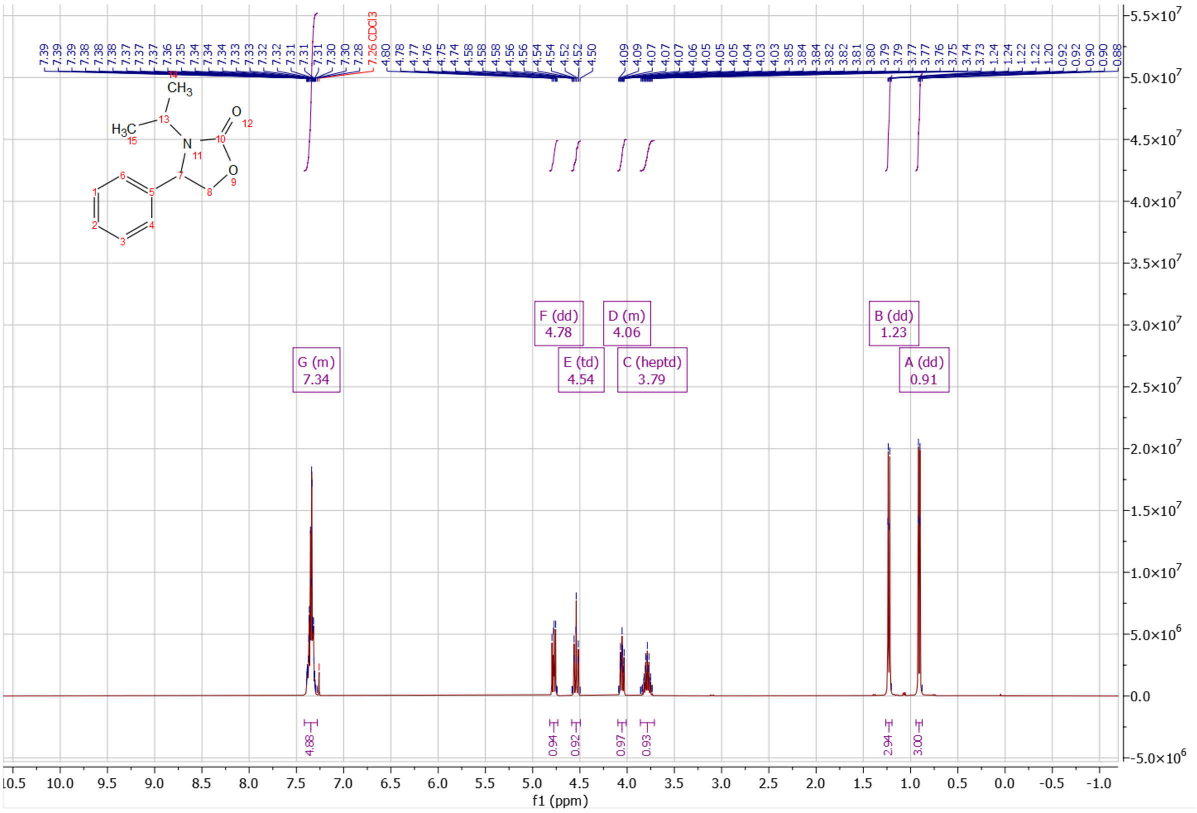

*Figure S8. ¹H NMR spectra of **2c**.*

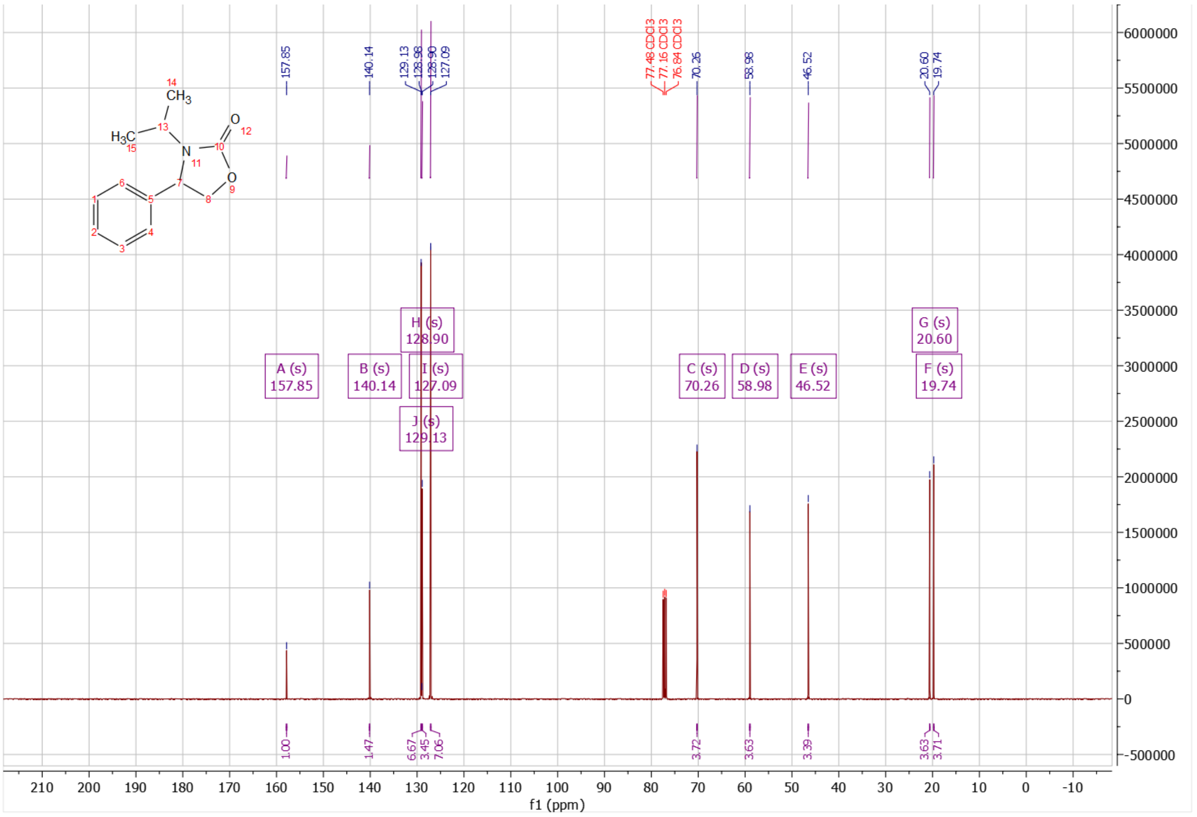

*Figure S9. ¹³C NMR spectra of **2c**.*

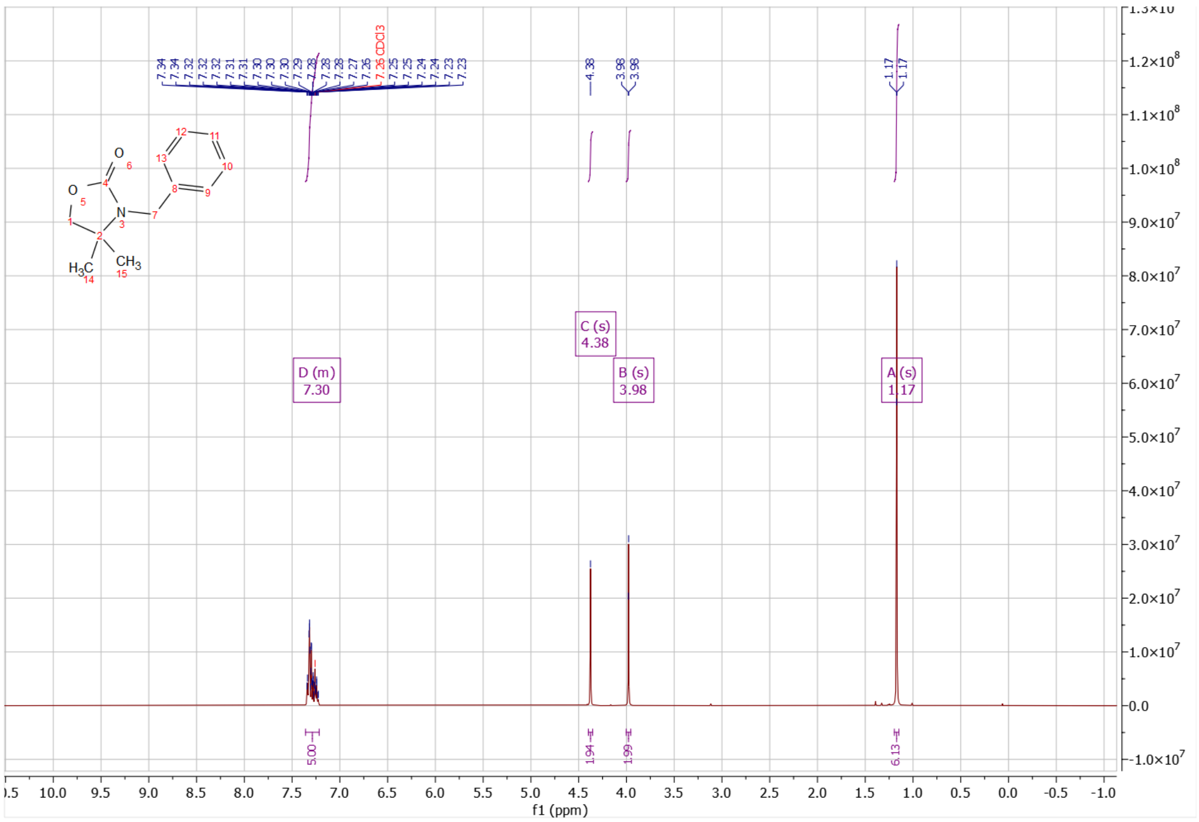

*Figure S10. $^{1}$H NMR spectra of **2d**.*

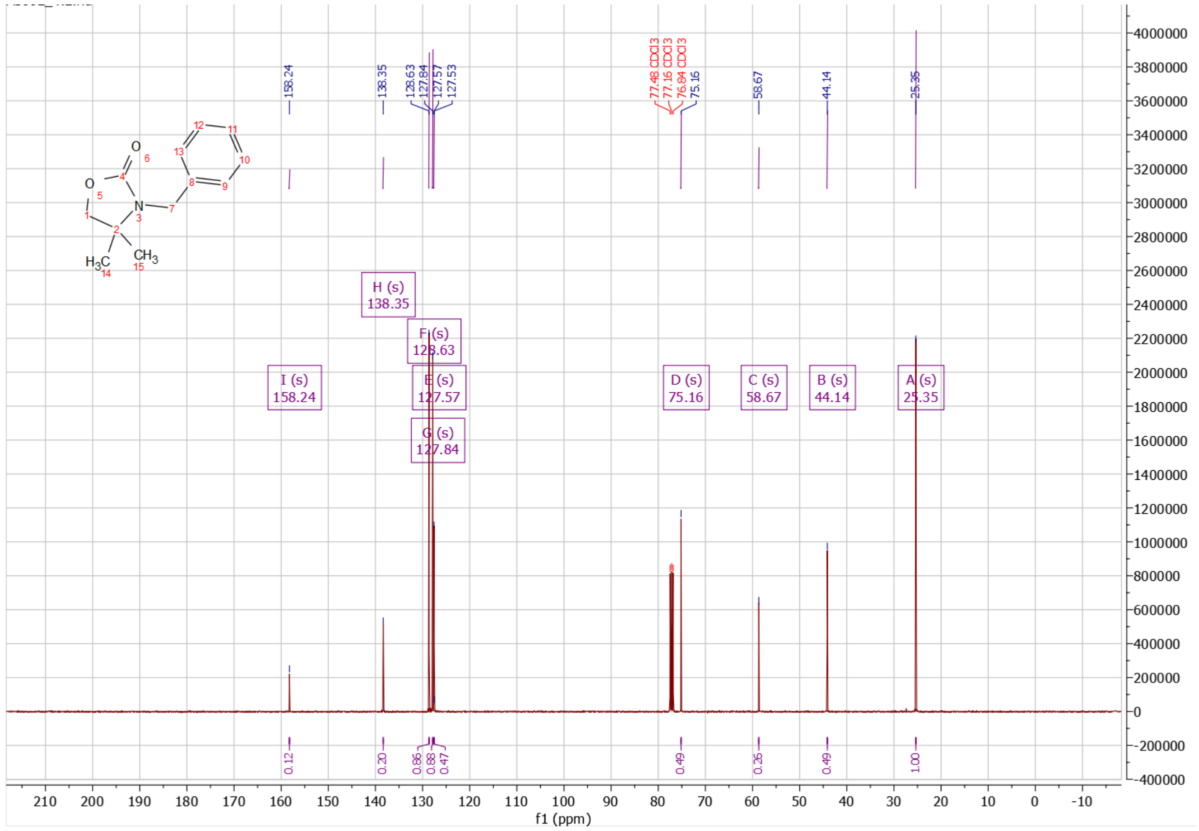

*Figure S11. $^{13}$C NMR spectra of **2d**.*

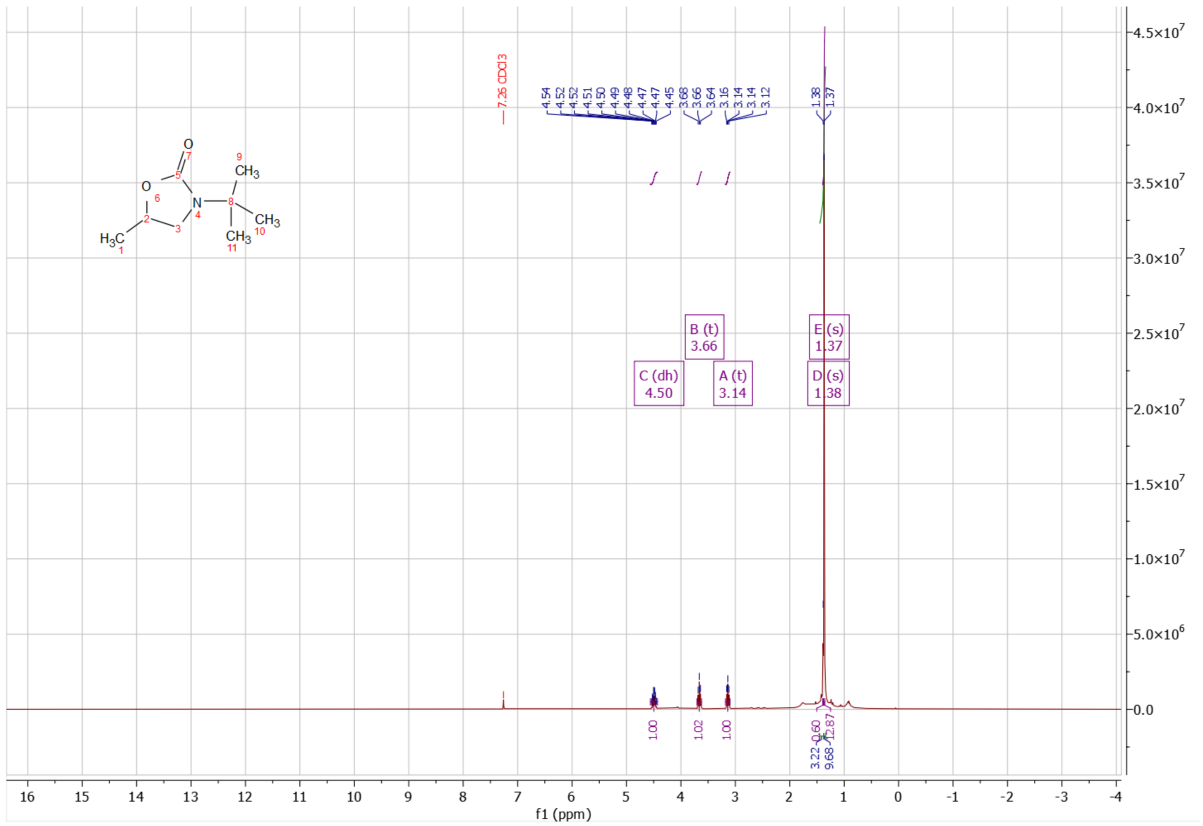


Figure S12. $^{1}H$ NMR spectra of **2e**.

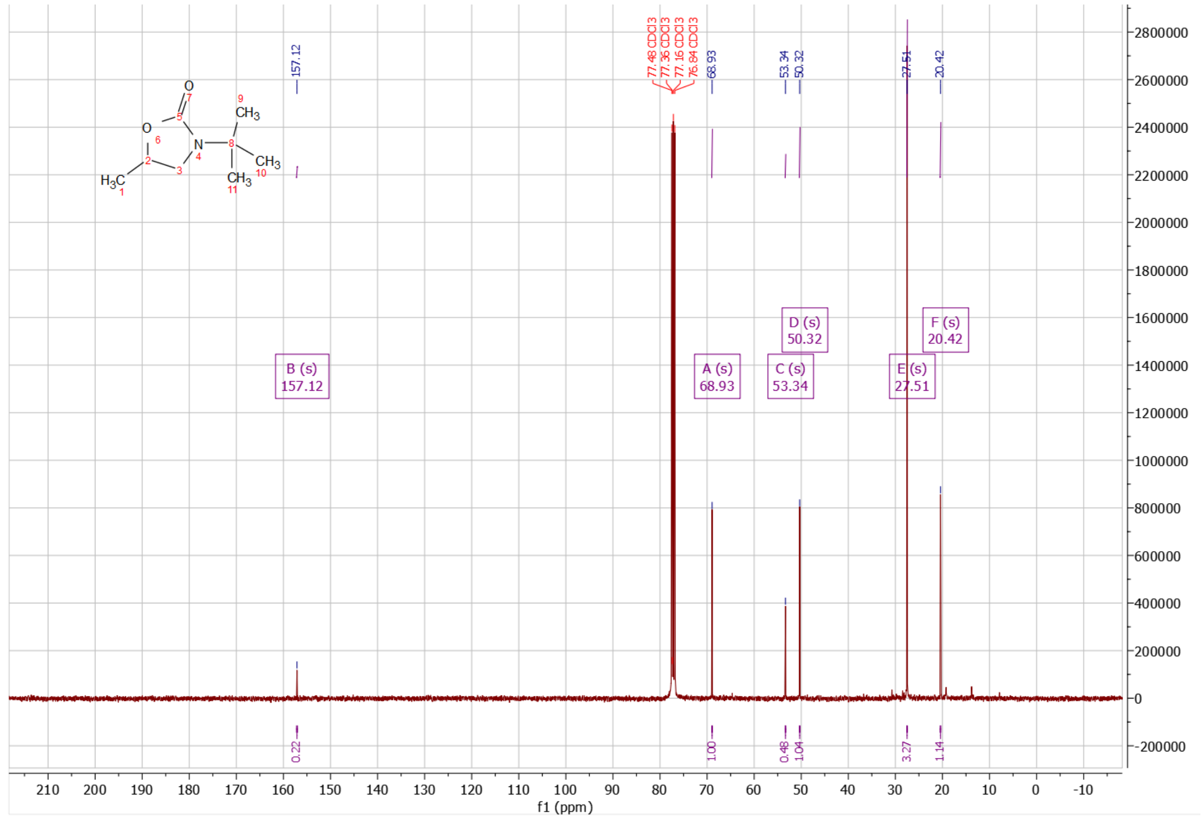


Figure S13. $^{13}C$ NMR spectra of **2e**.

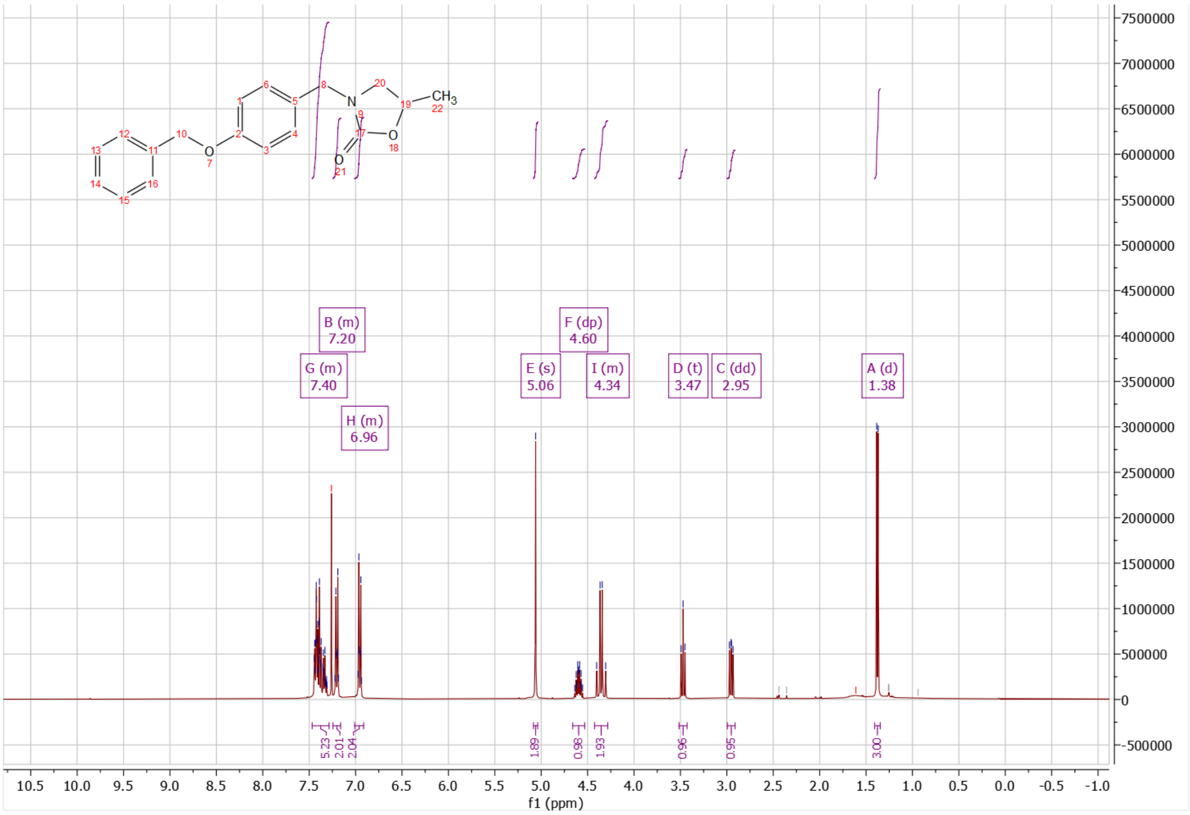


*Figure S14. $^{1}$H NMR spectra of **2f**.*

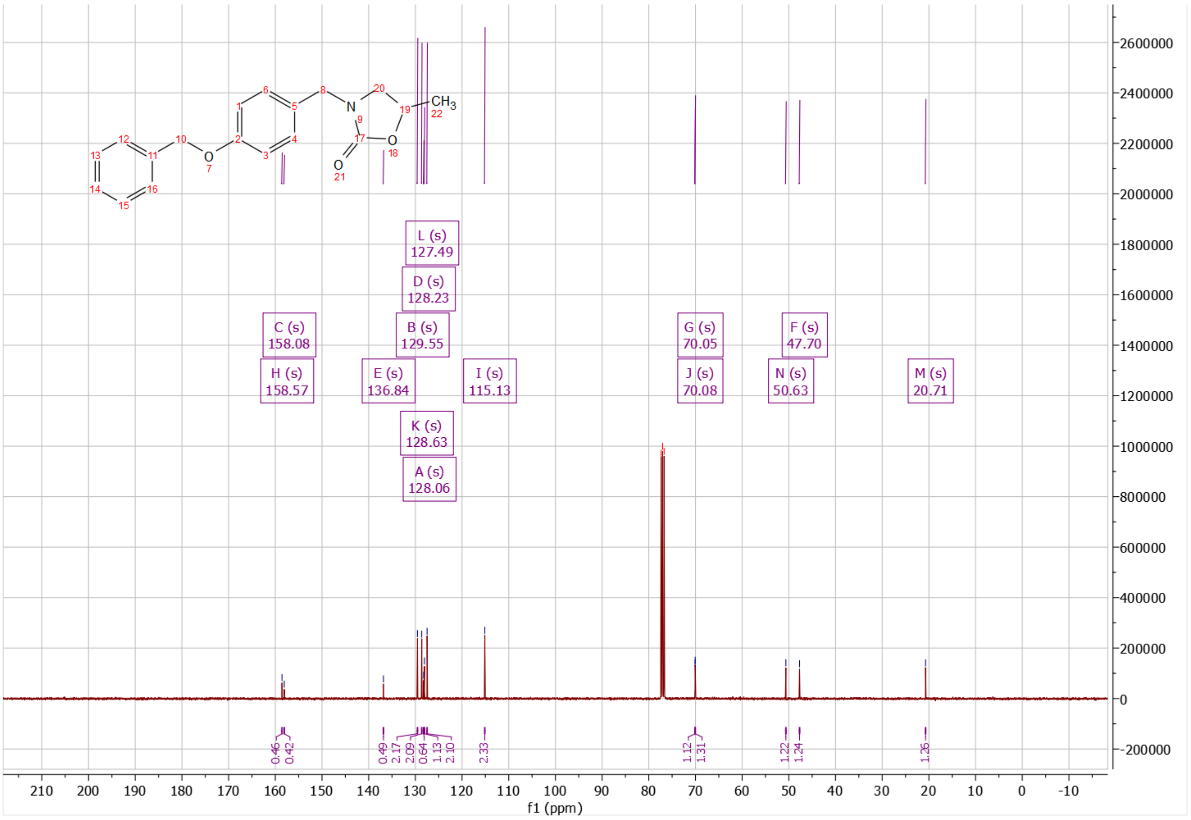


*Figure S15. $^{13}$C NMR spectra of **2f**.*

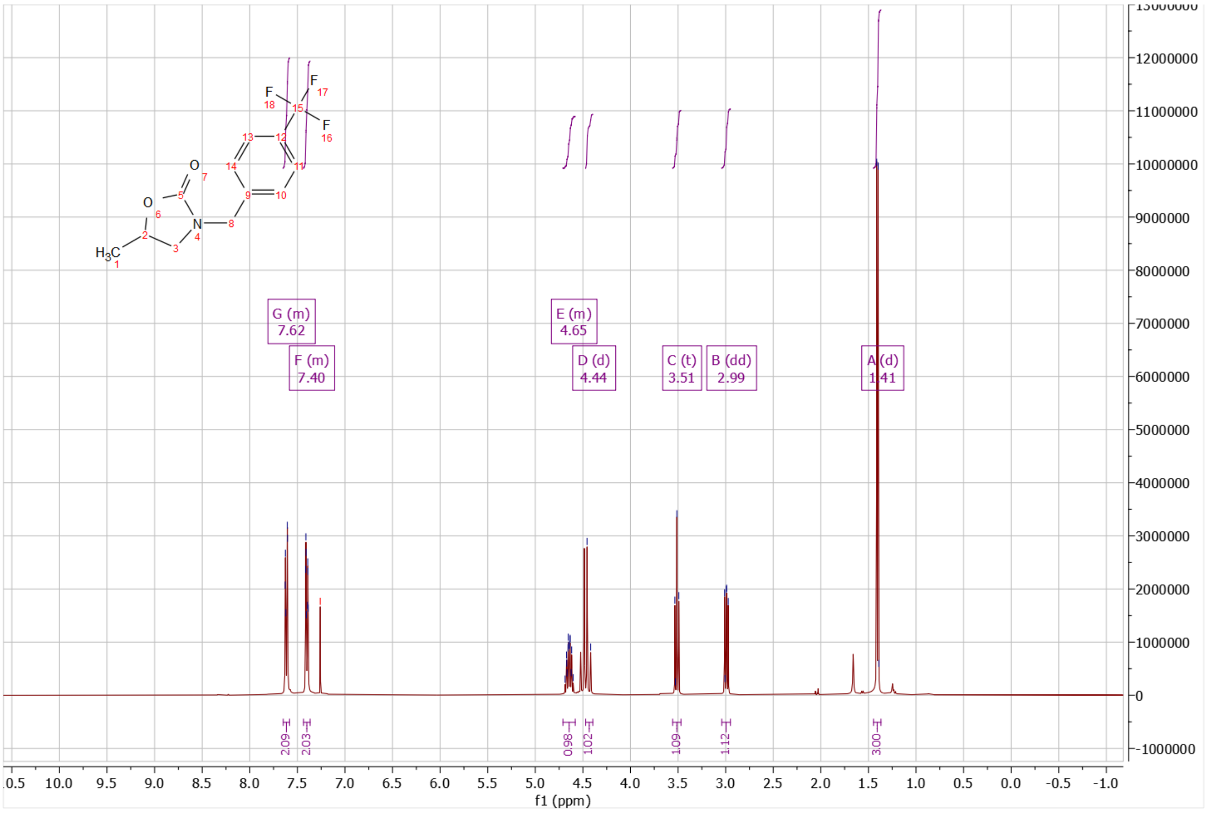

*Figure S16. $^{1}$H NMR spectra of **2g**.*

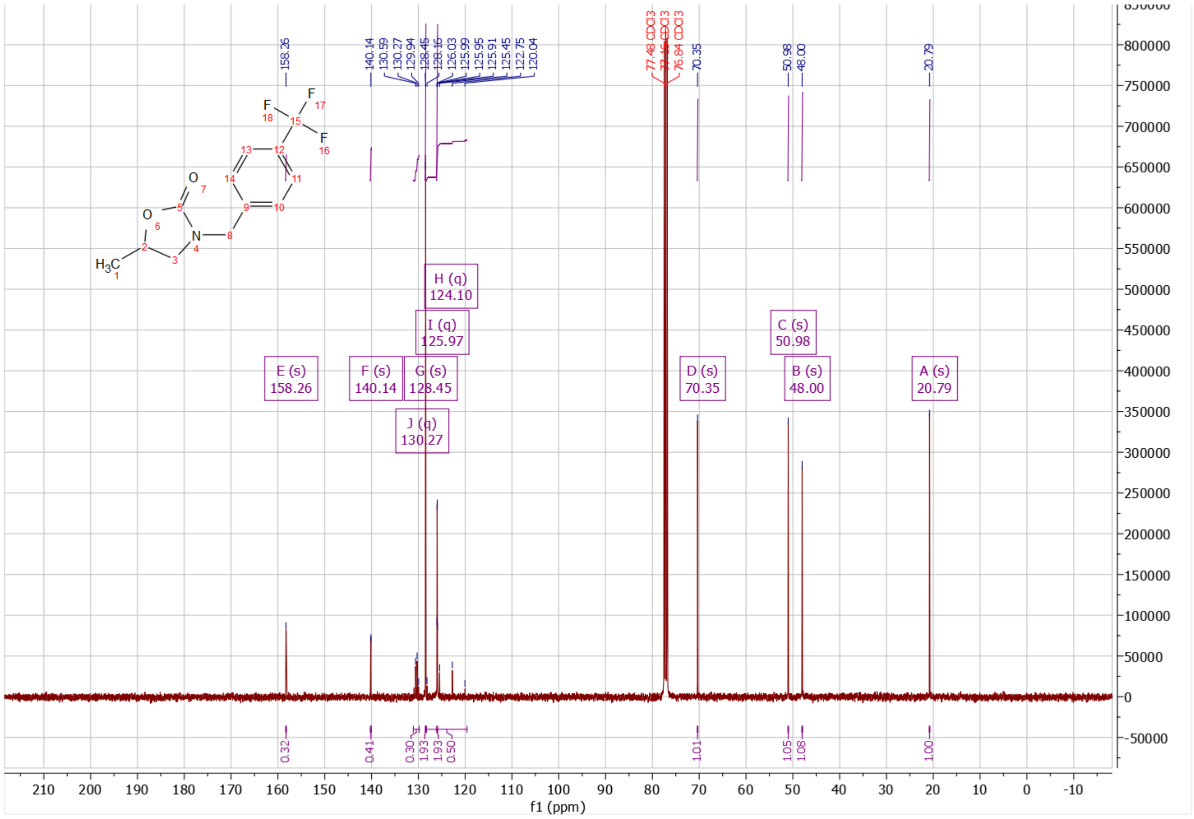

*Figure S17. $^{13}$C NMR spectra of **2g**.*

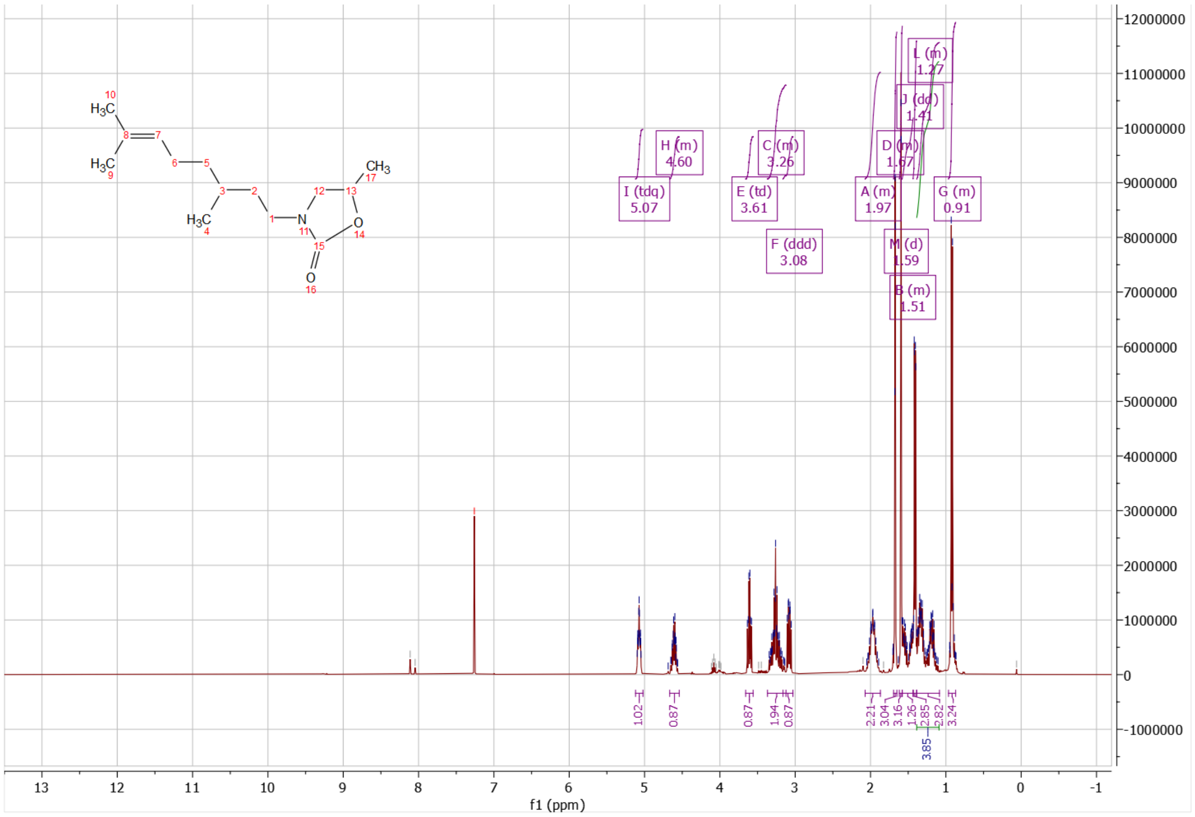

*Figure S18. $^{1}$H NMR spectra of **2h**.*

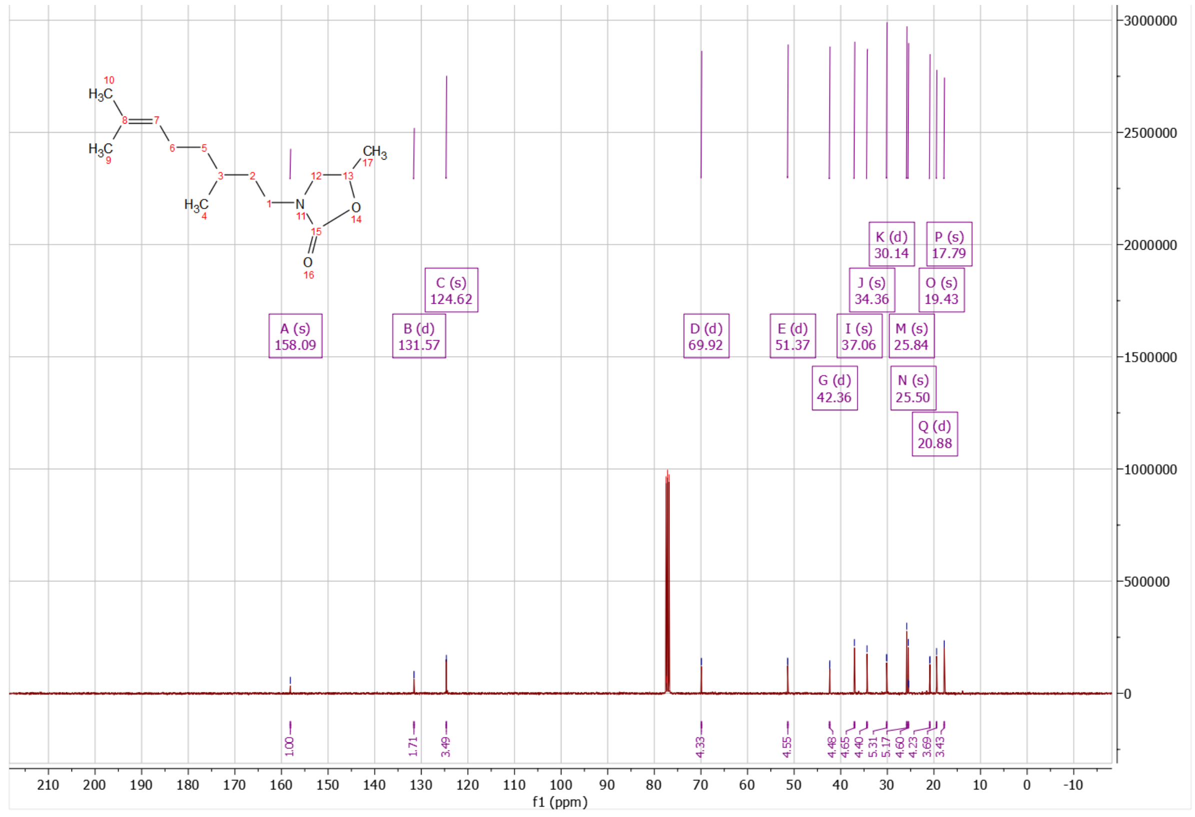

*Figure S19. $^{13}$C NMR spectra of **2h**.*

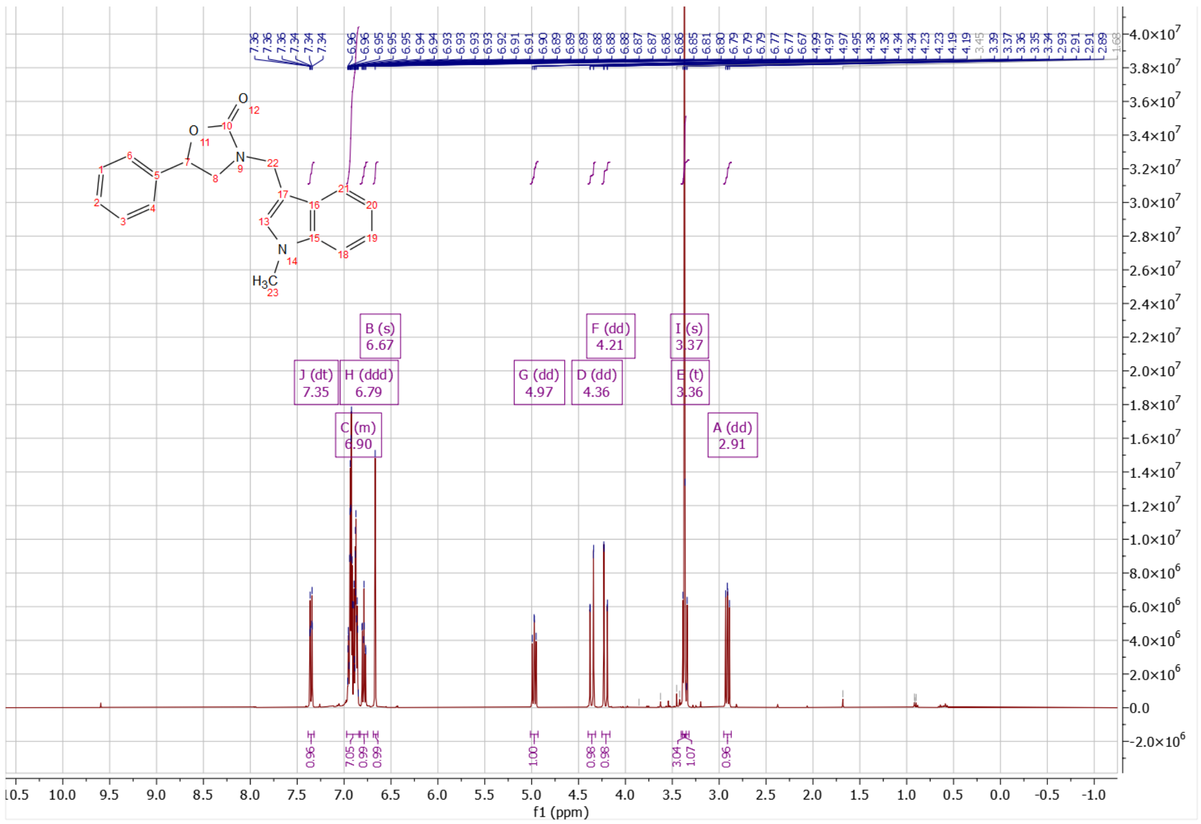

*Figure S20. 1h NMR spectra of **2i**.*

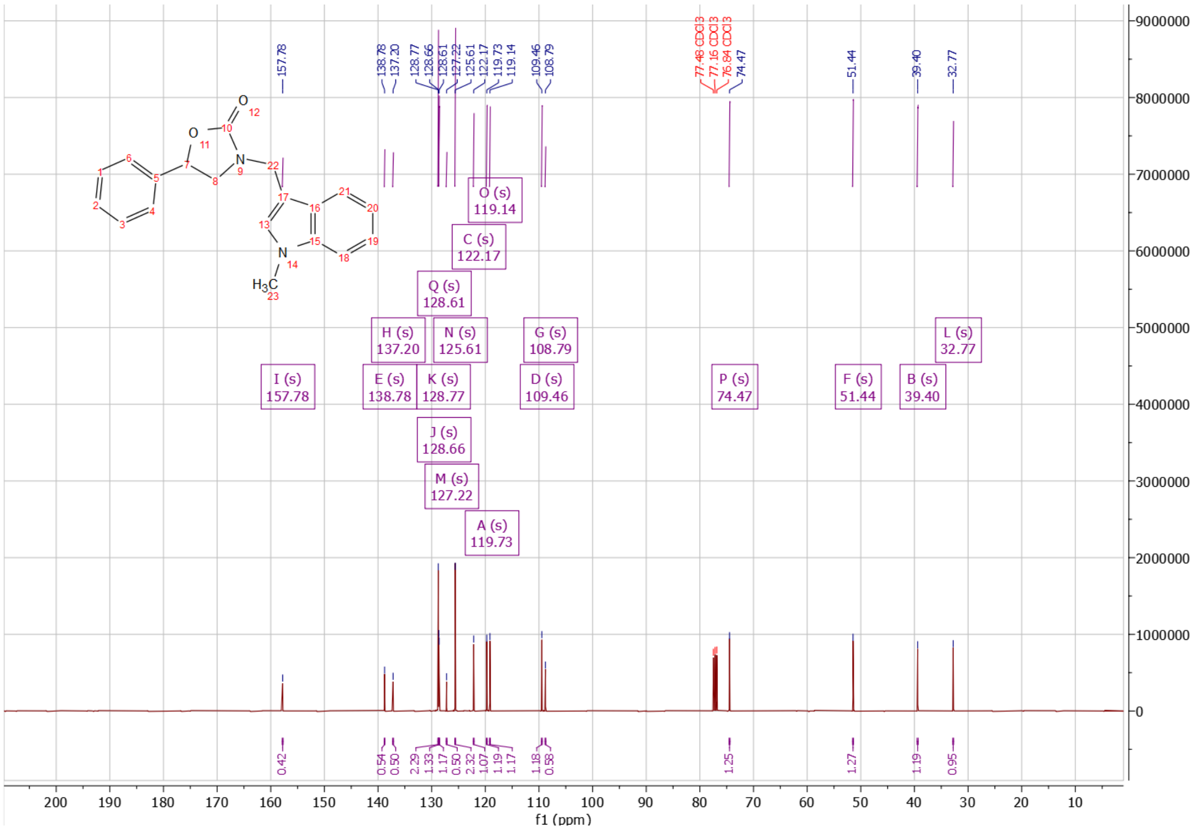

*Figure S21. $^{13}$C NMR spctra of **2i**.*

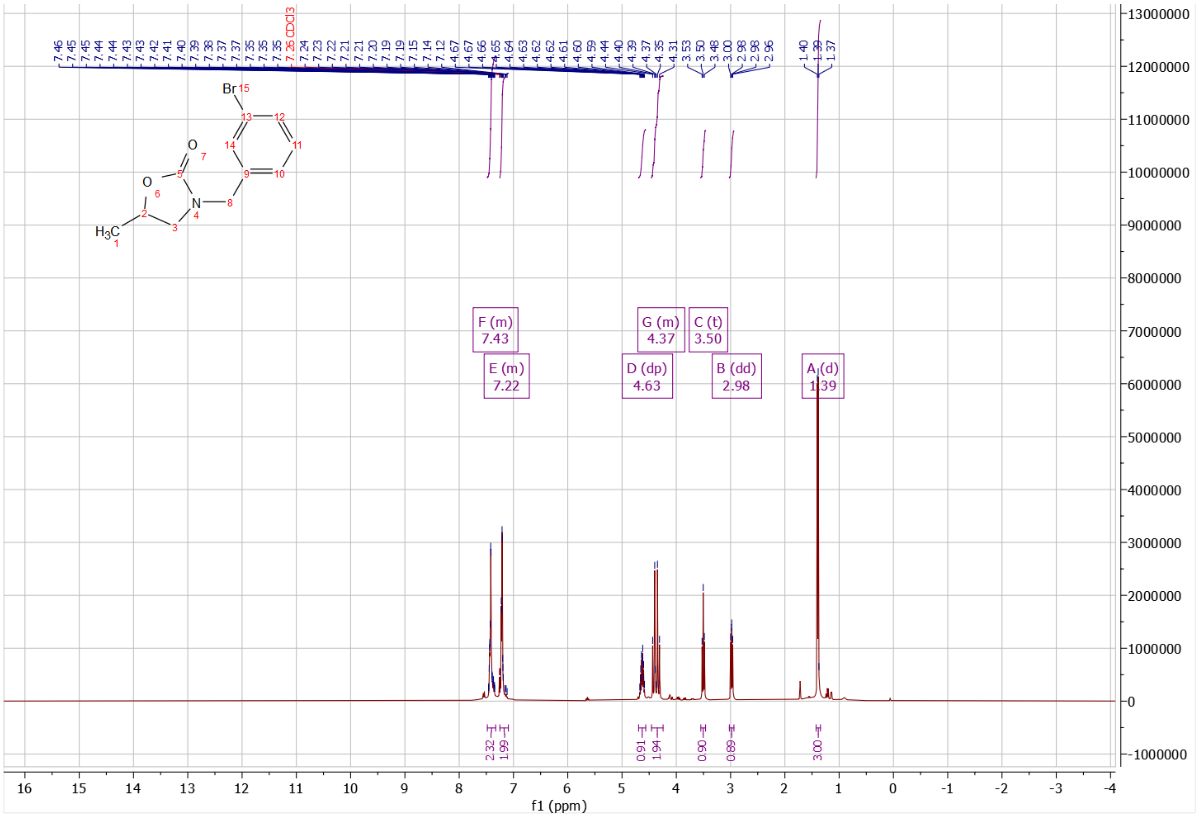

*Figure S22.* $^{1}H$ *NMR spetra of* ***2j****.*

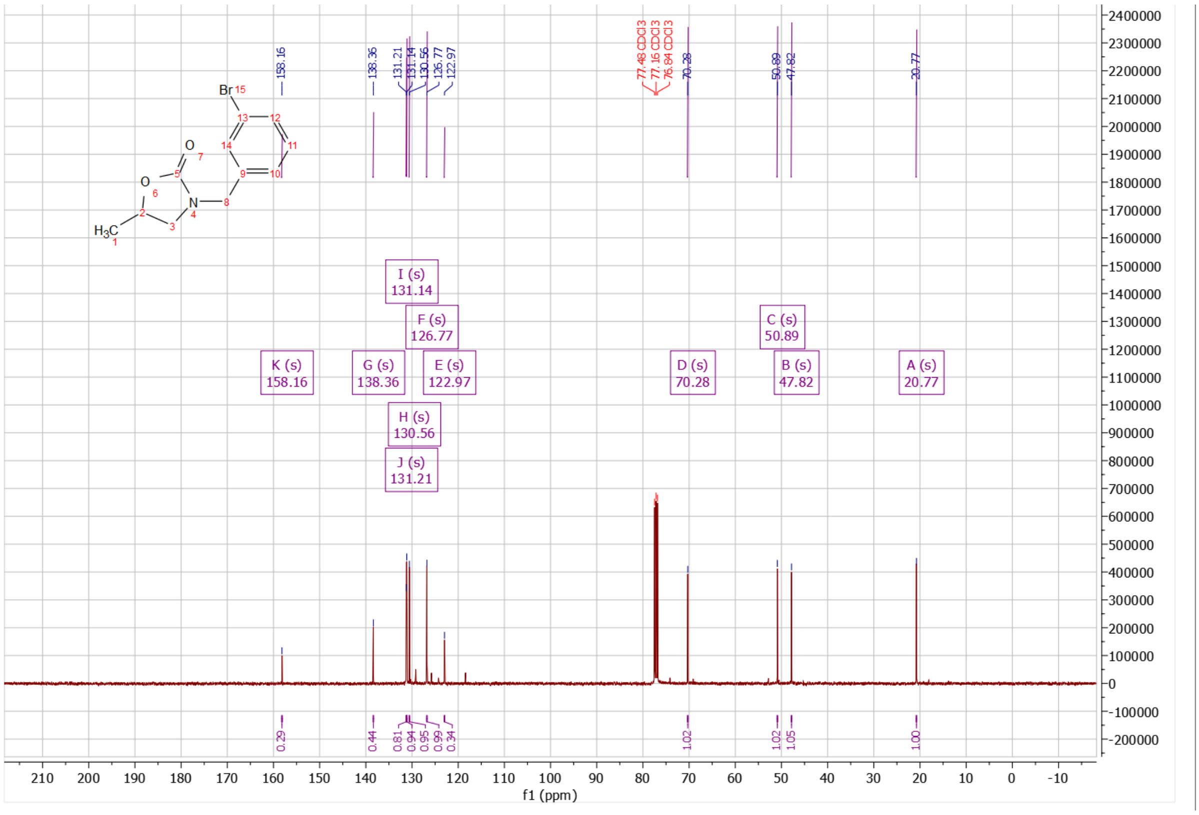

*Figure S 23.* $^{13}C$ *NMR spectra of* ***2j****.*

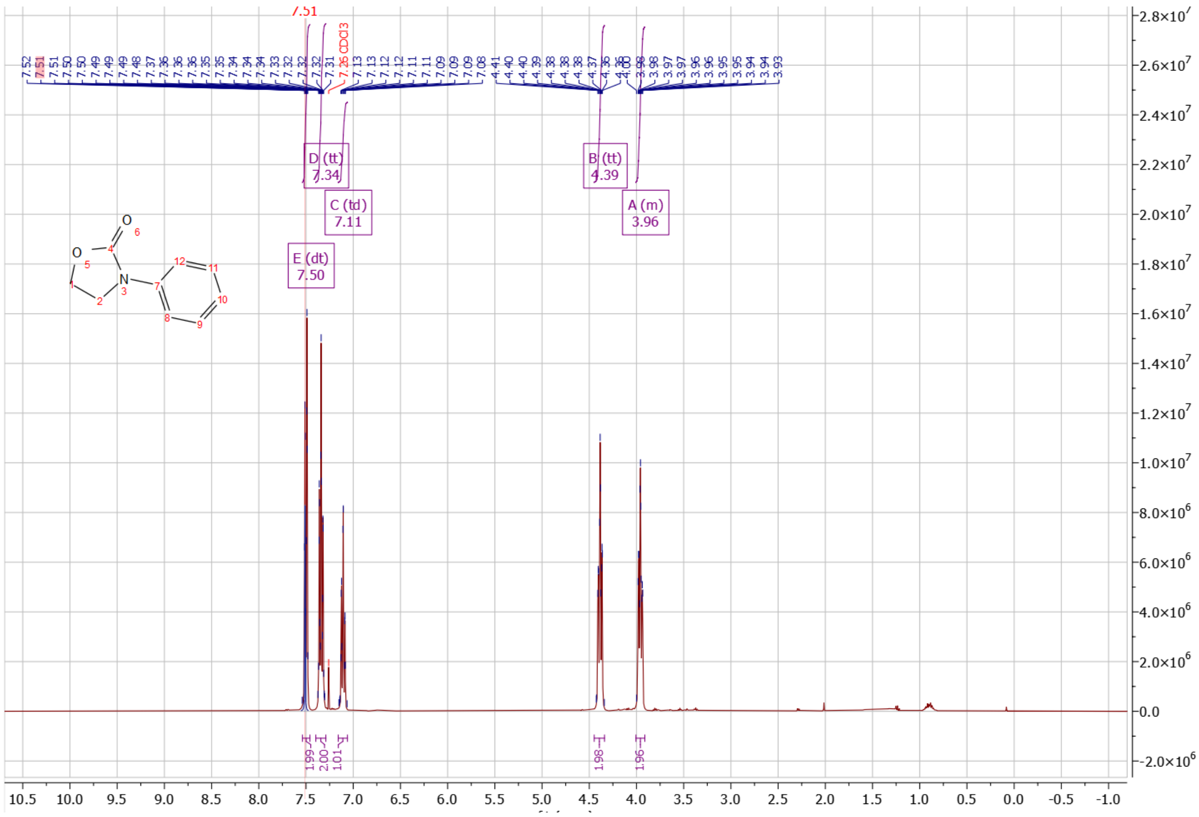

*Figure S24.* $^{1}H$ *NMR spectra of* ***2k****.*

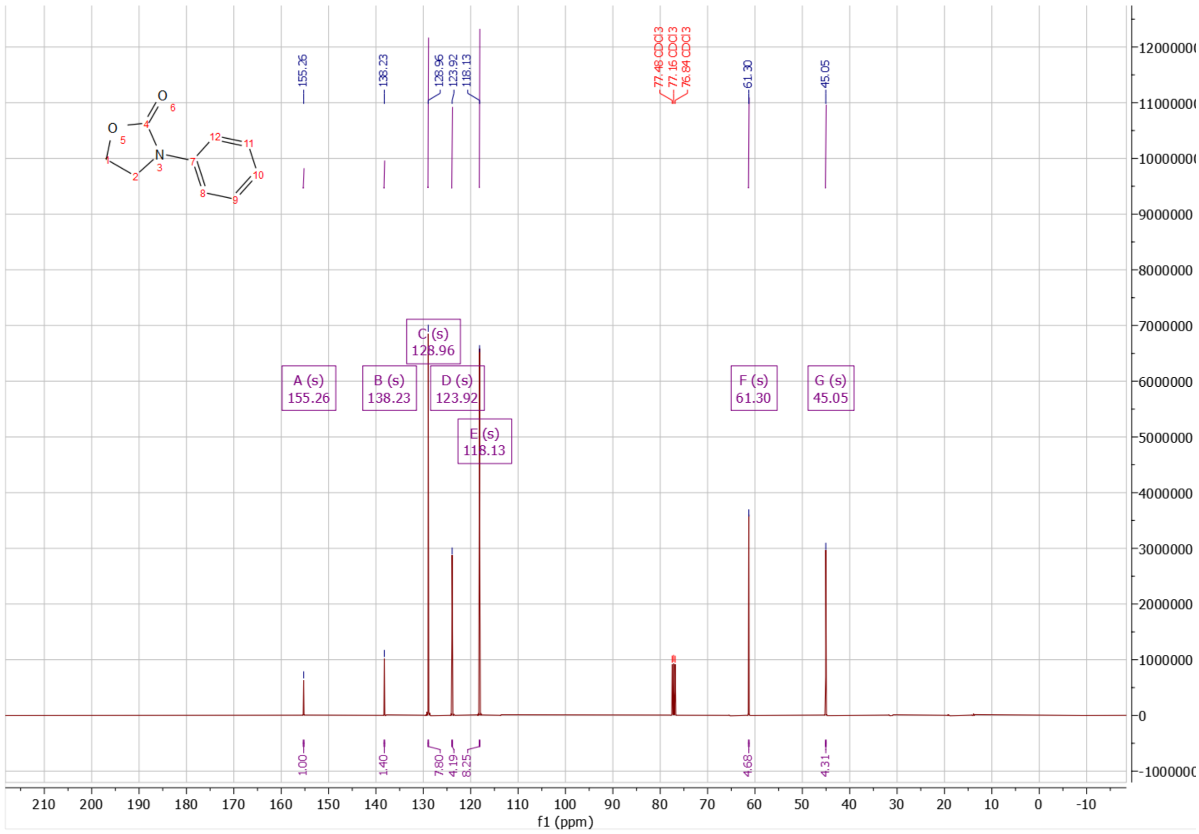

*Figure S25.* $^{13}C$ *NMR spectra of* ***2k****.*

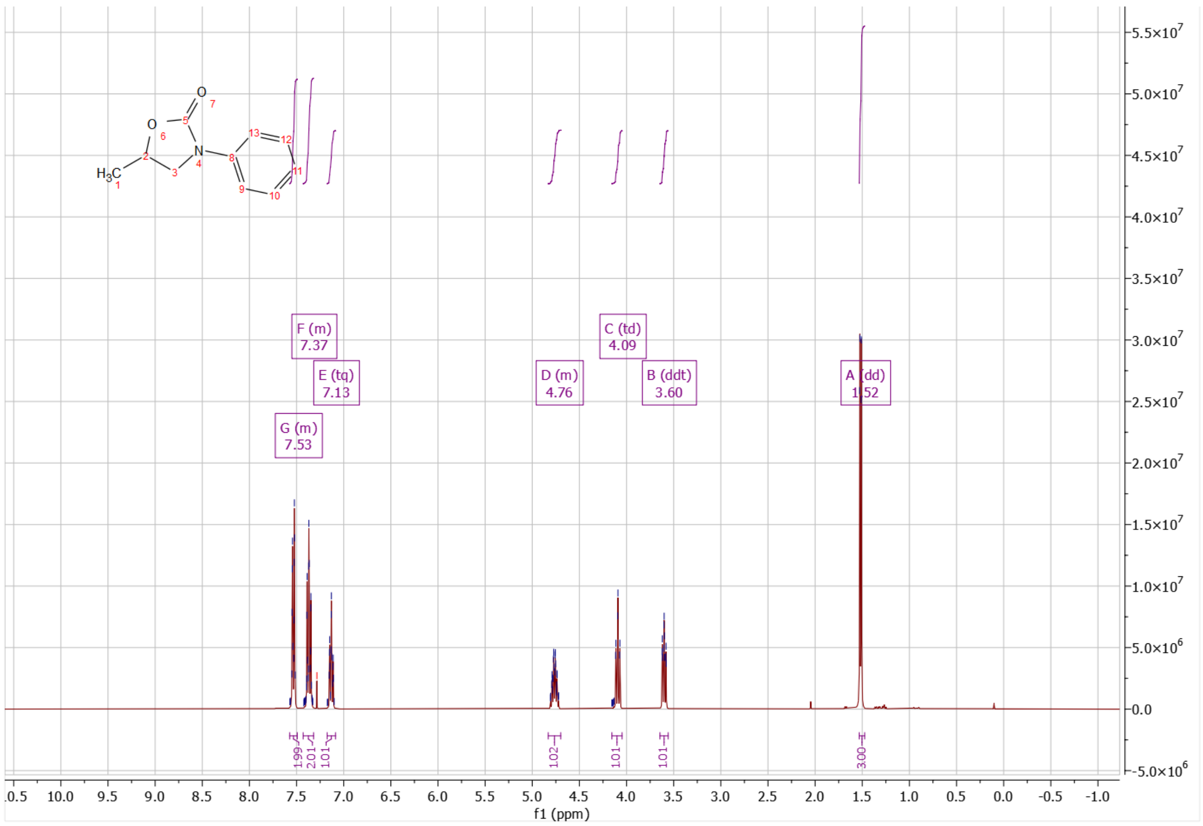


*Figure S26. $^{1}$H NMR spectra of **2l**.*

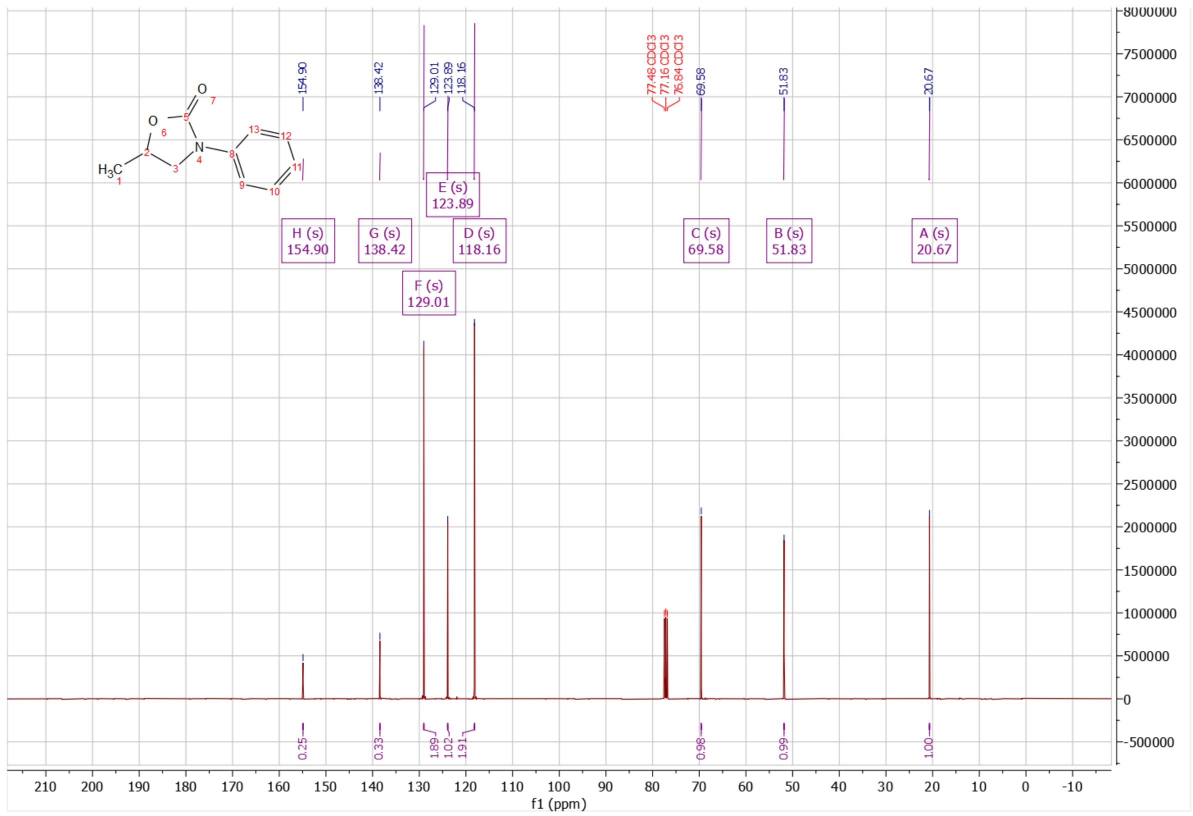


*Figure S27. $^{13}$C NMR spectra of **2l**.*

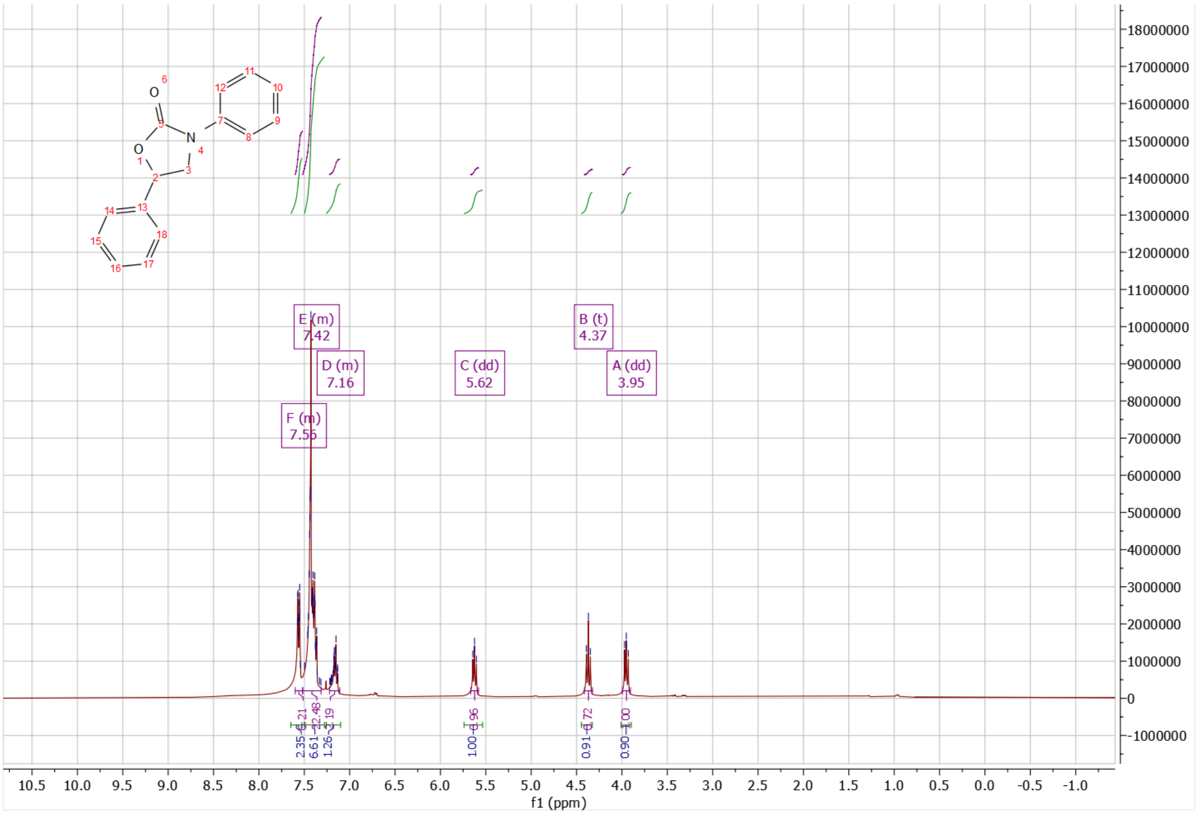


*Figure S28.* [1]*H NMR spectra of* ***2m***.

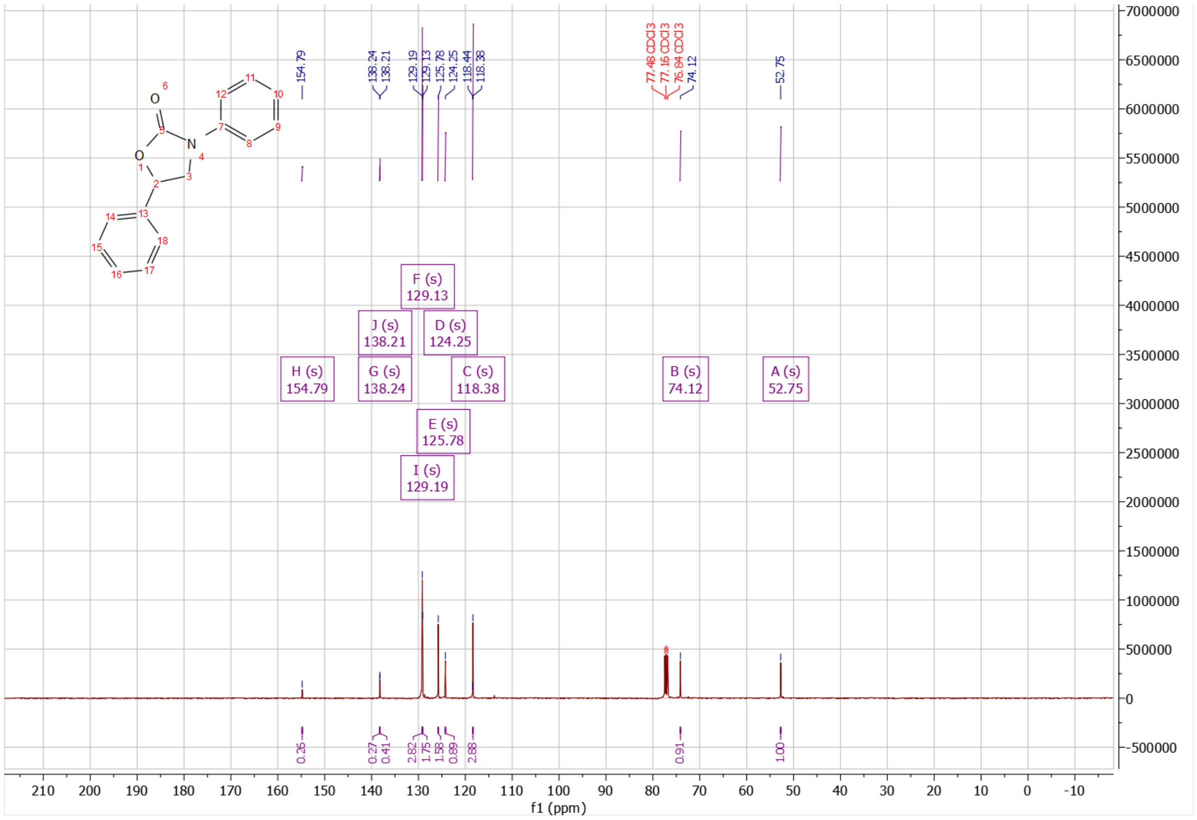


*Figure S29.* [13]*C NMR spectra of* ***2m***.

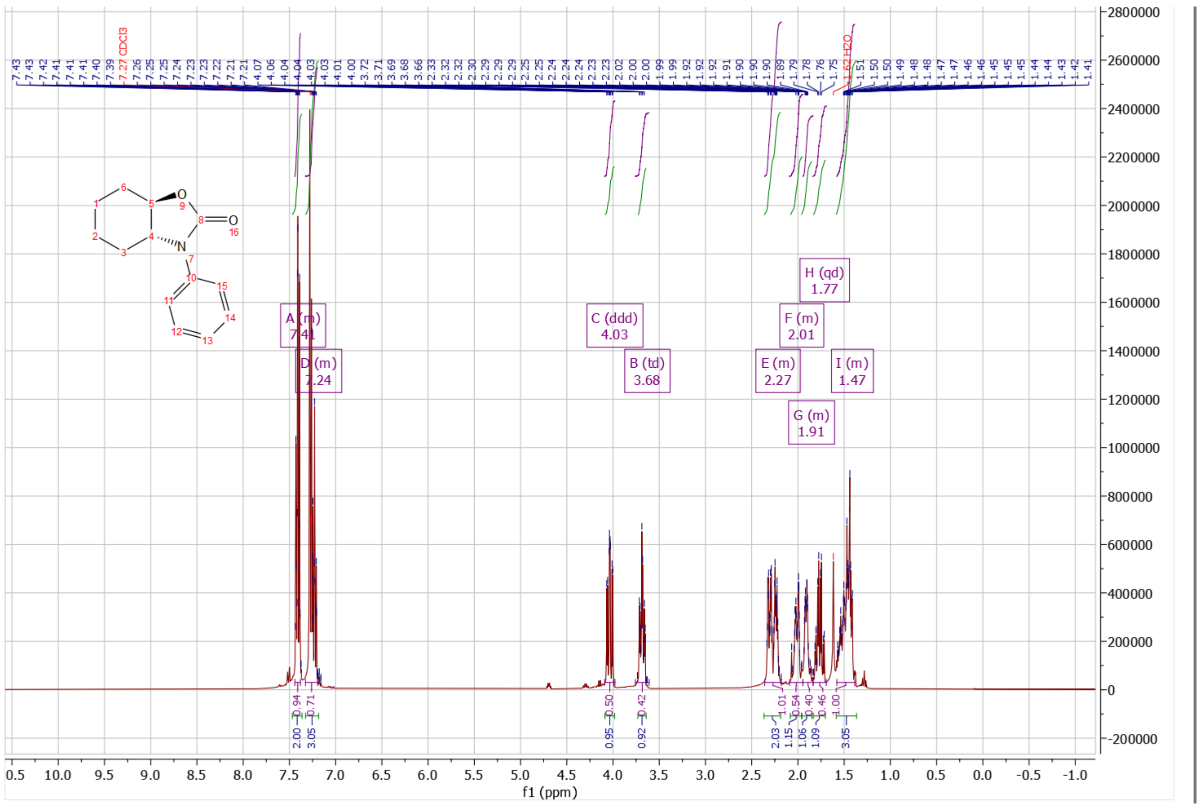

*Figure S 30. $^{1}$H NMR spectra of **2n**.*

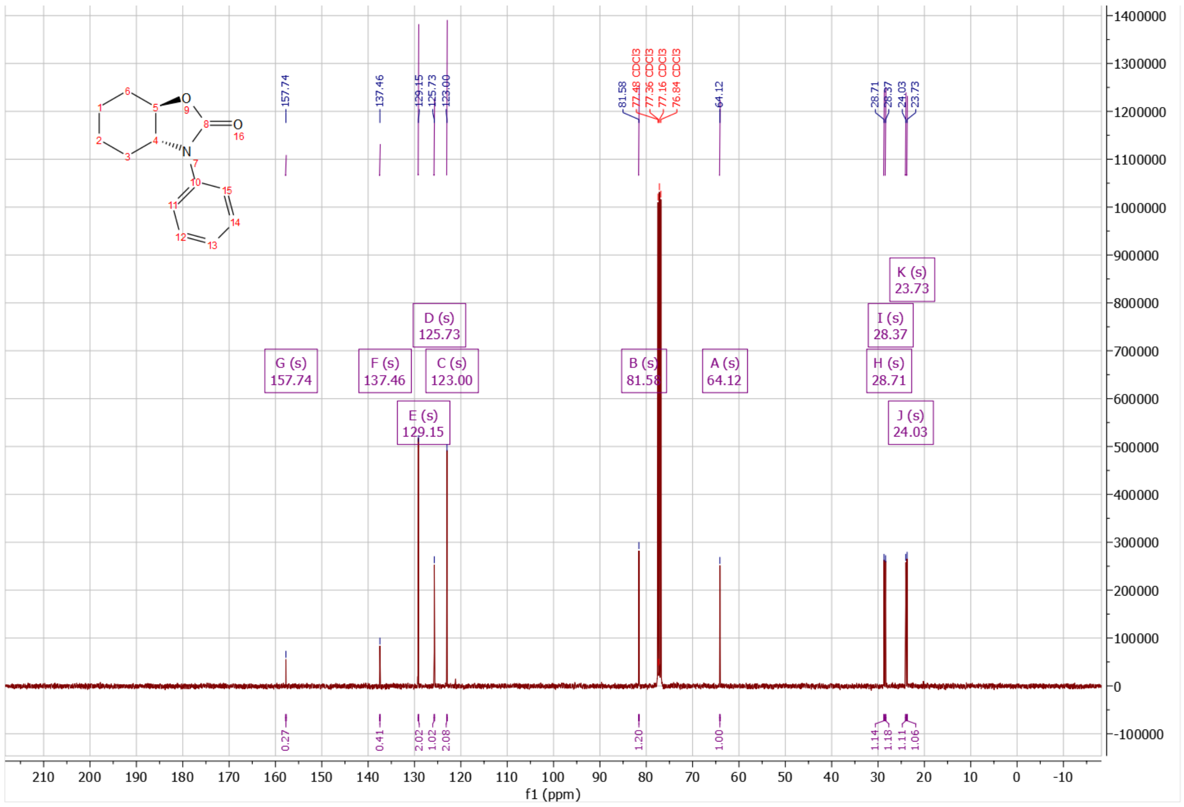

*Figure S31. $^{13}$C NMR spectra of **2n**.*

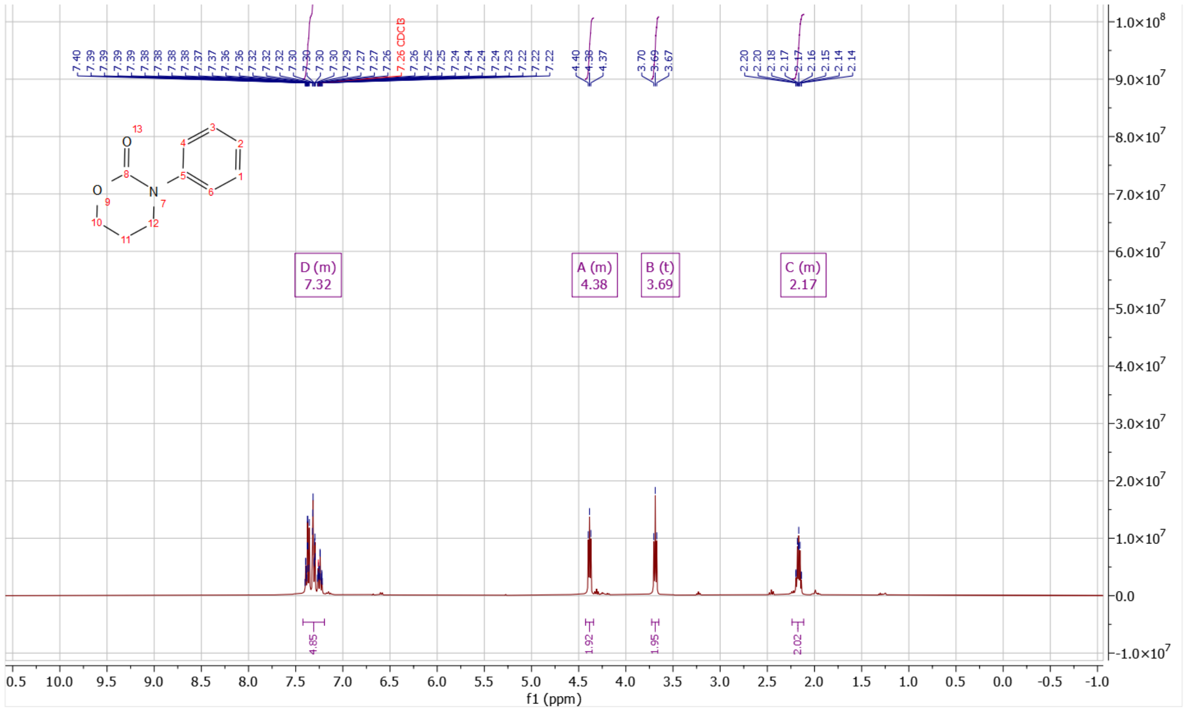

*Figure S32. $^{1}$H NMR spcetra of **2o**.*

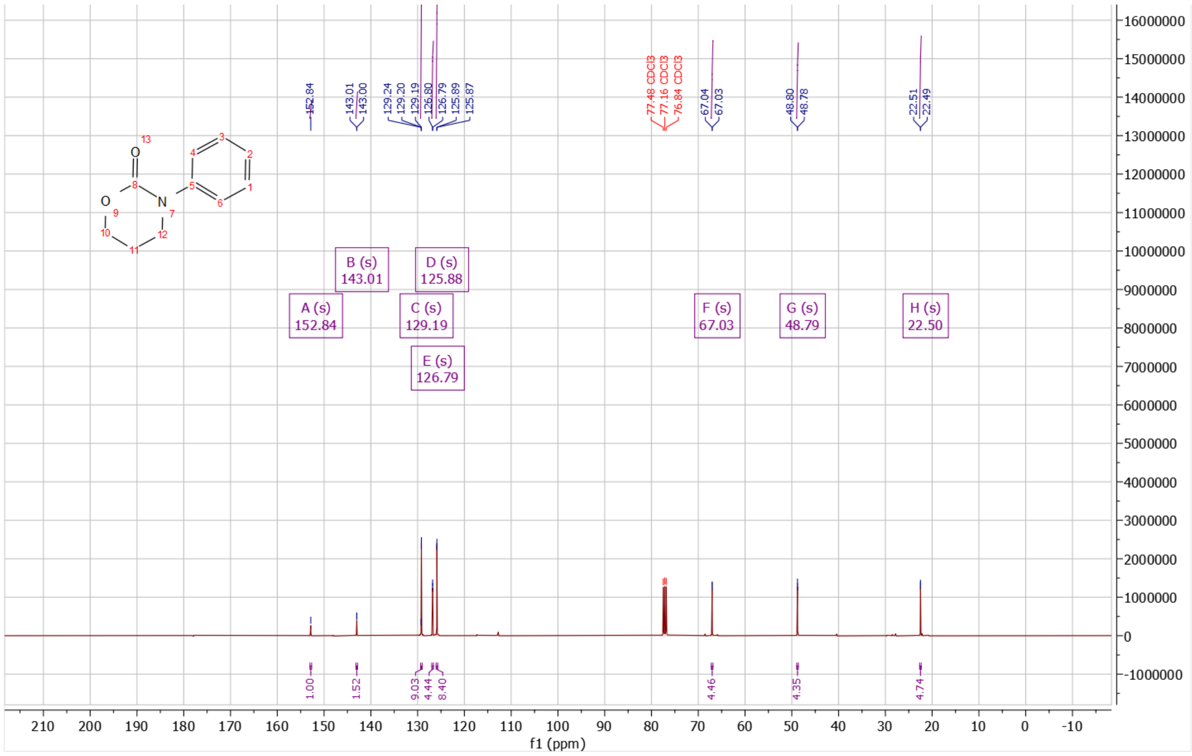

*Figure S33. $^{13}$C NMR spectra of **2o**.*

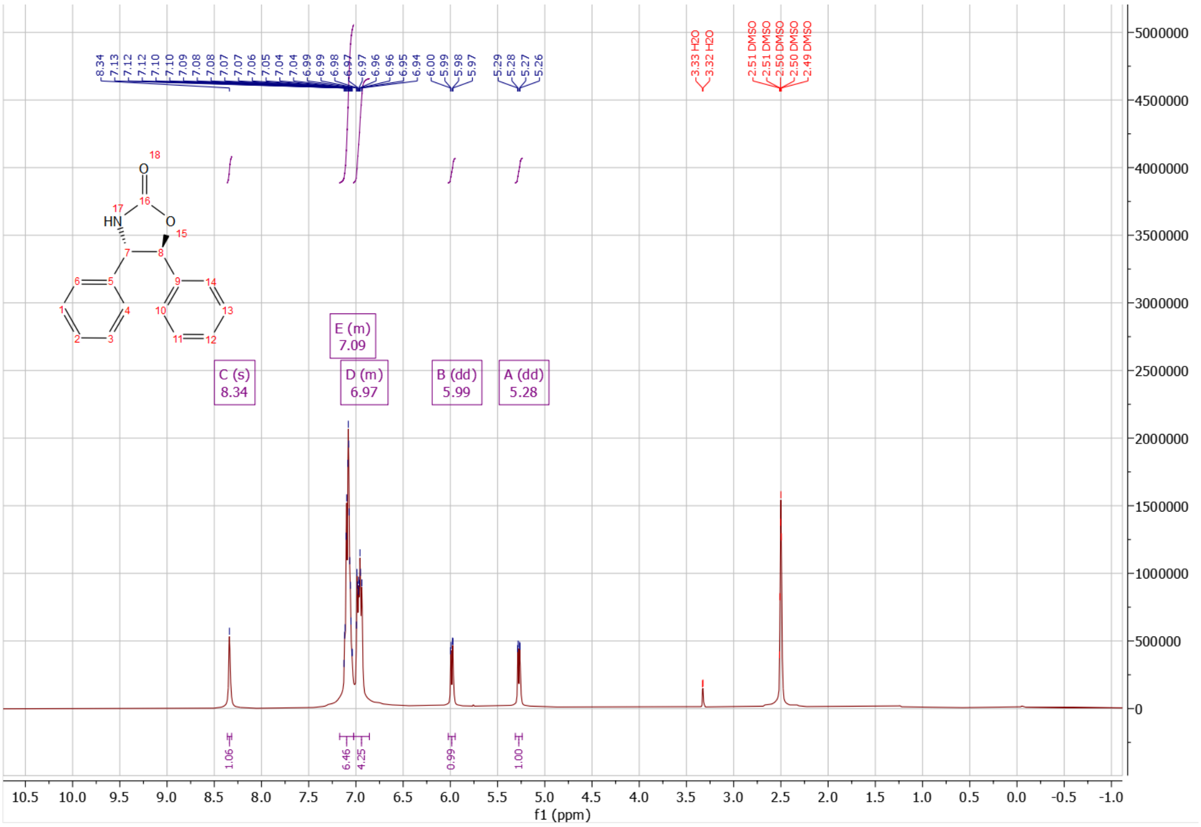

*Figure S34. ¹H NMR spectra of **2p**.*

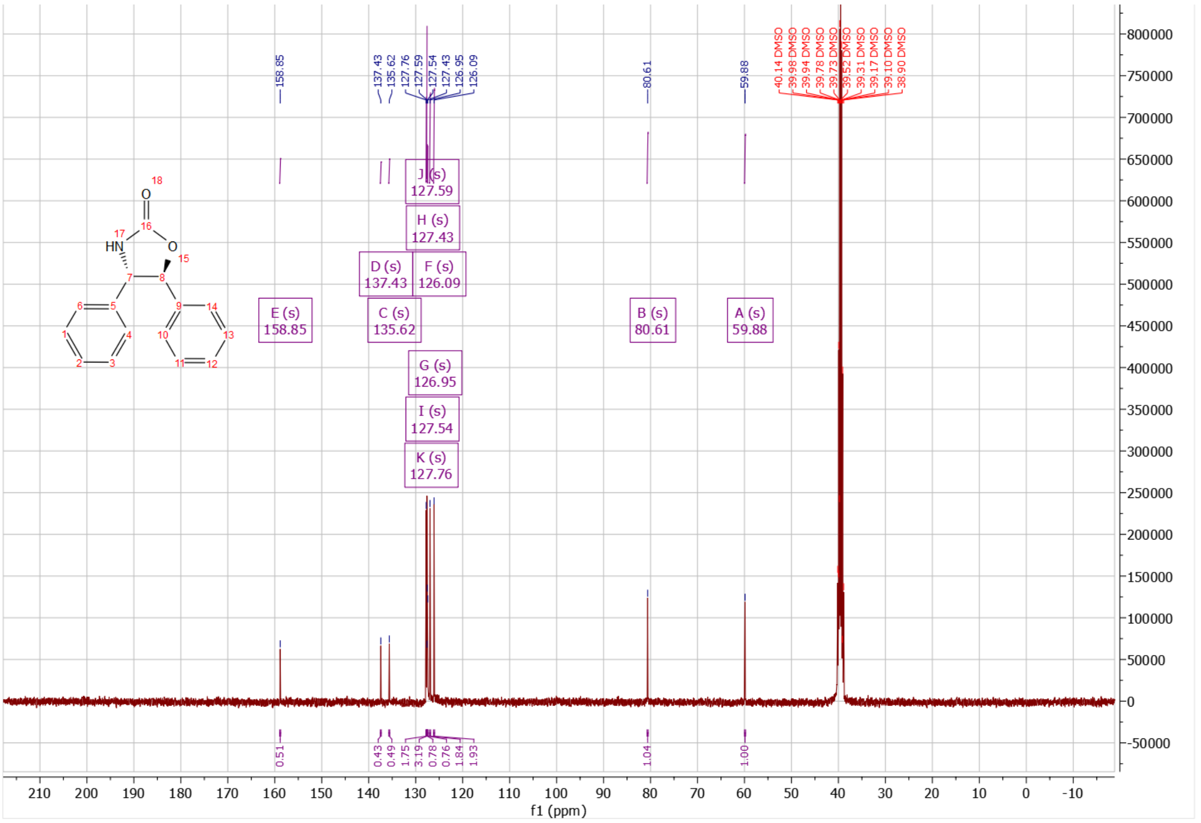

*Figure S35. ¹³c NMR spctra of **2p**.*

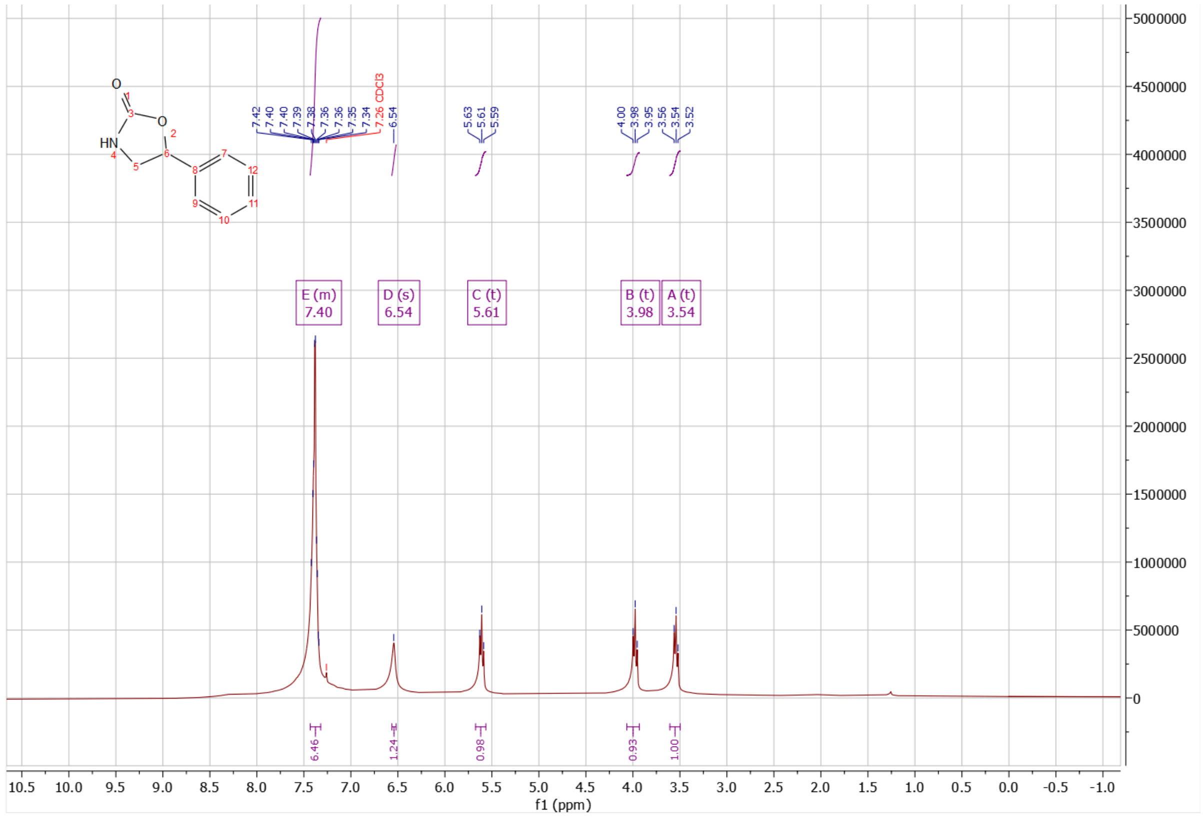

*Figure S 36. $^{1}$H NMR spectra of **2q**.*

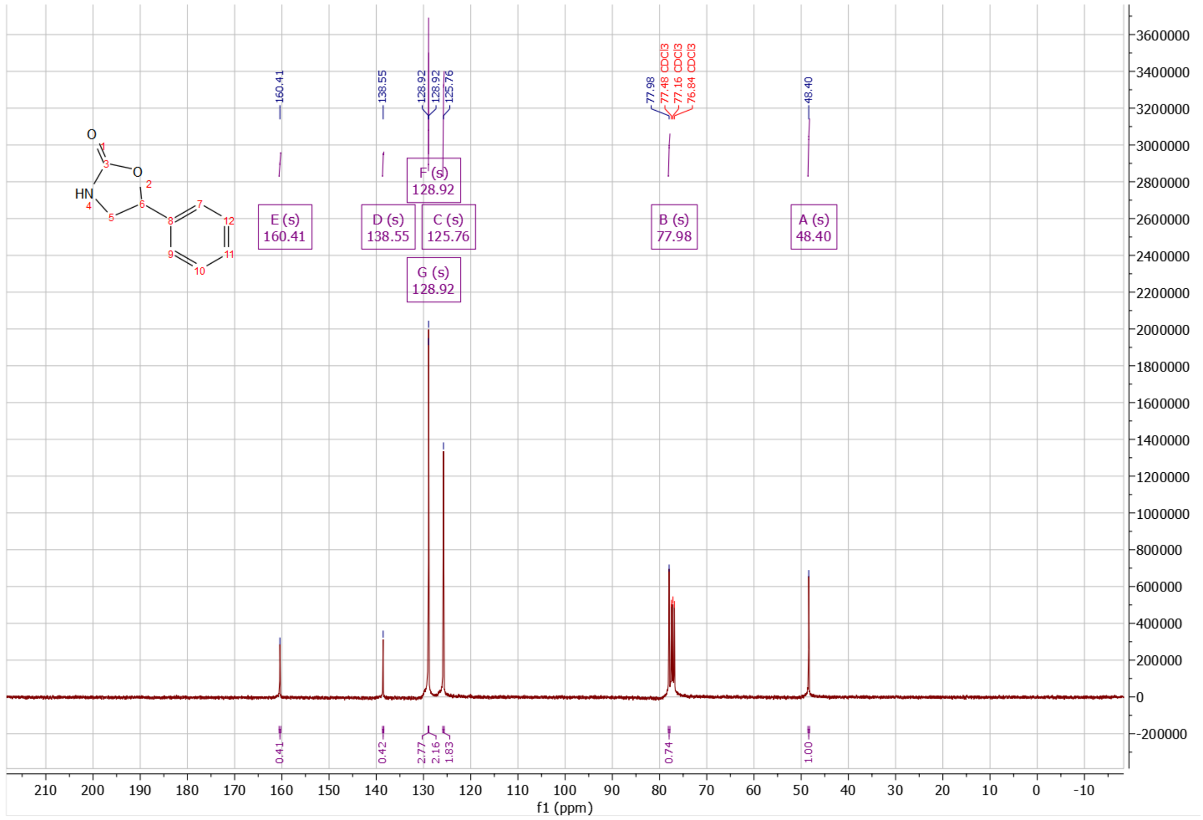

*Figure S37. $^{13}$C NMR spectra of **2q**.*

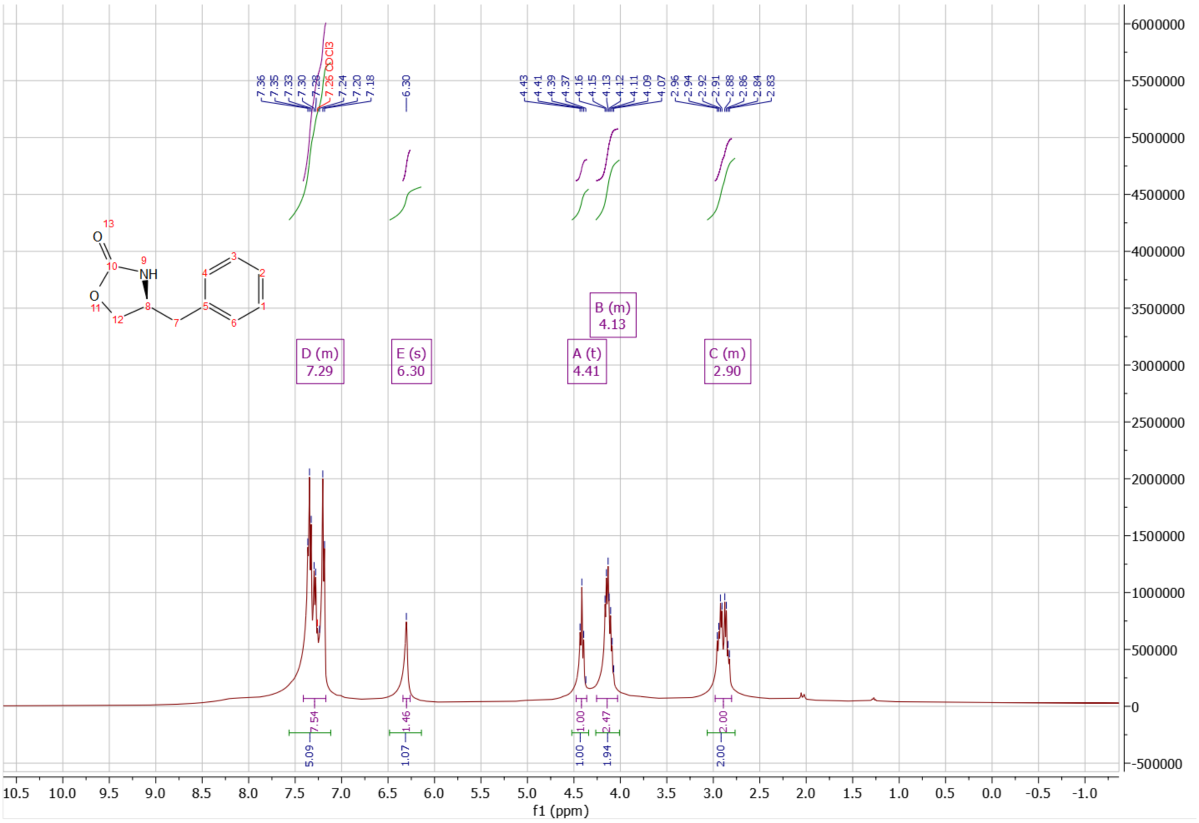


*Figure S38.* [1]*H NMR spectra of* ***2r****.*

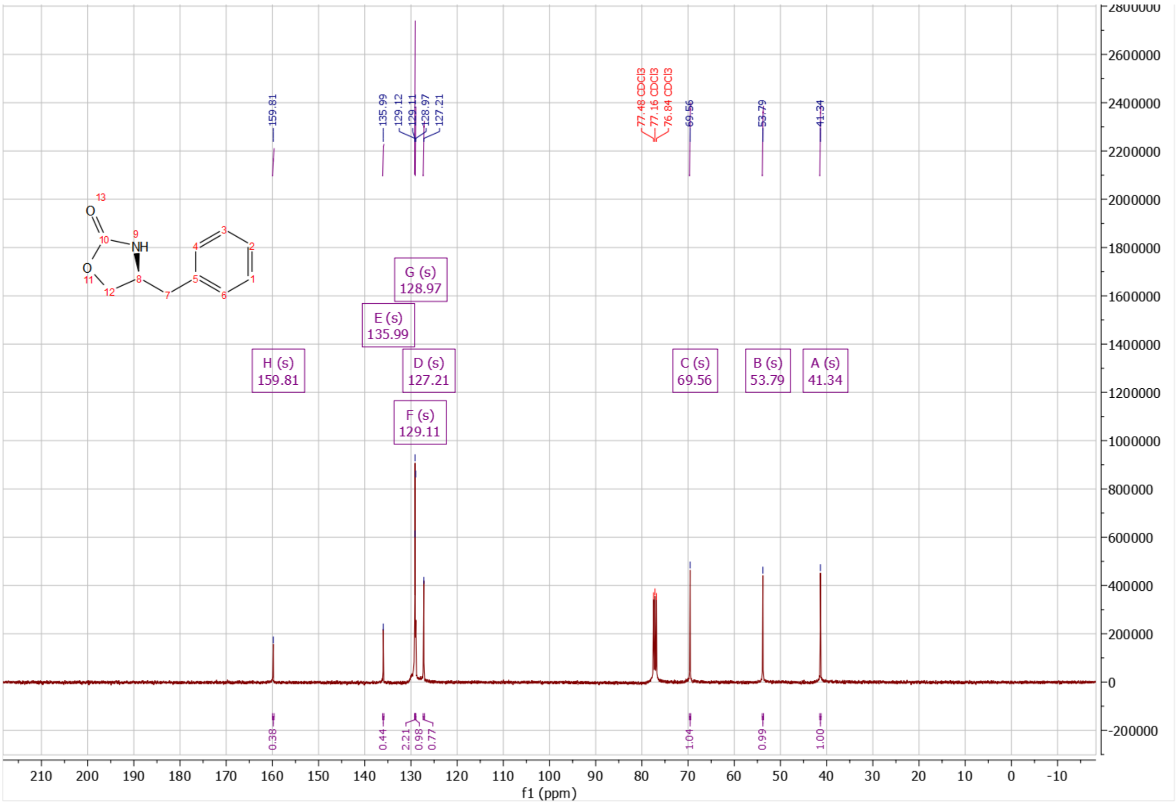


*Figure S39.* [13]*C NMR spectra of* ***2r****.*

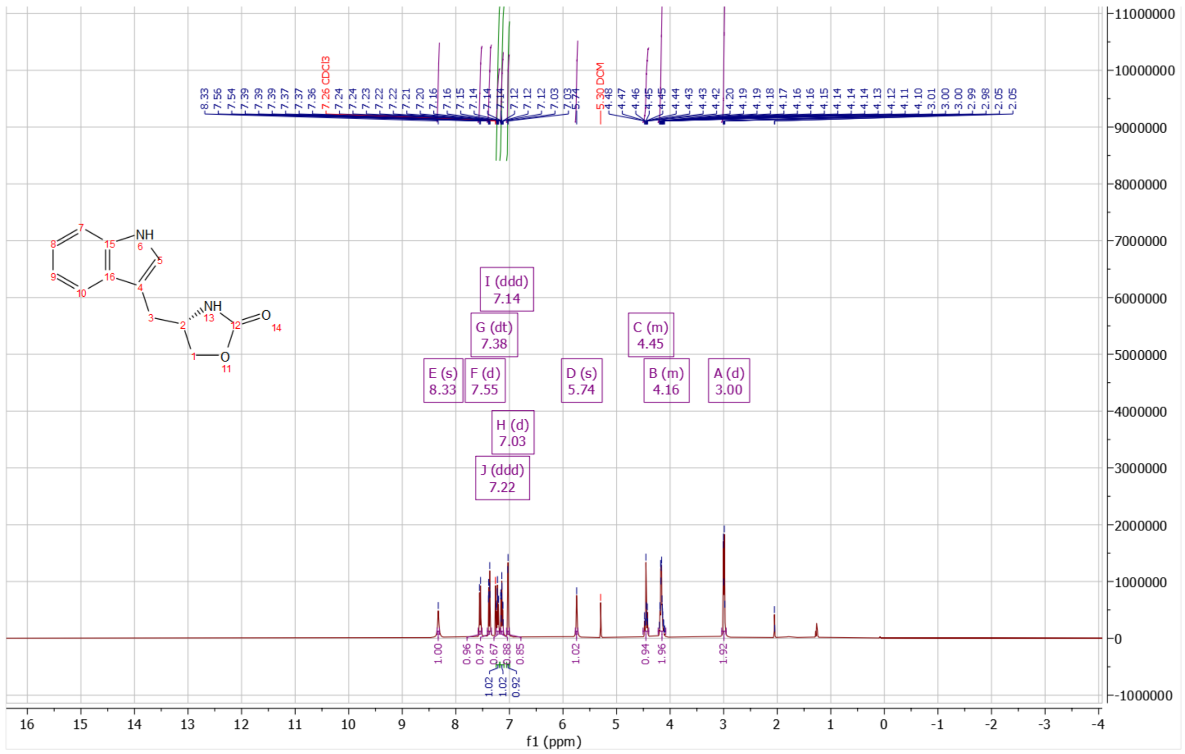

*Figure S40. $^{1}$H NMR spectra of **2s**.*

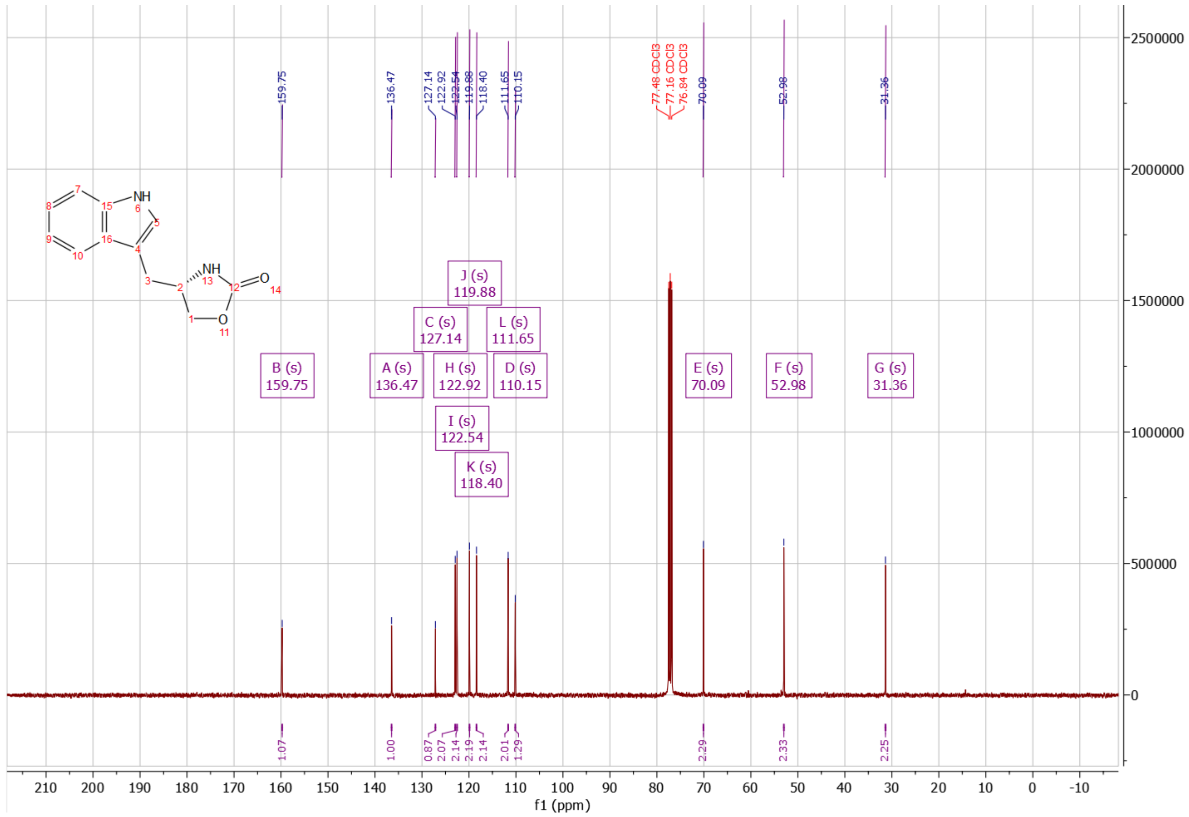

*Figure S41. $^{13}$C NMR spectra of **2s**.*

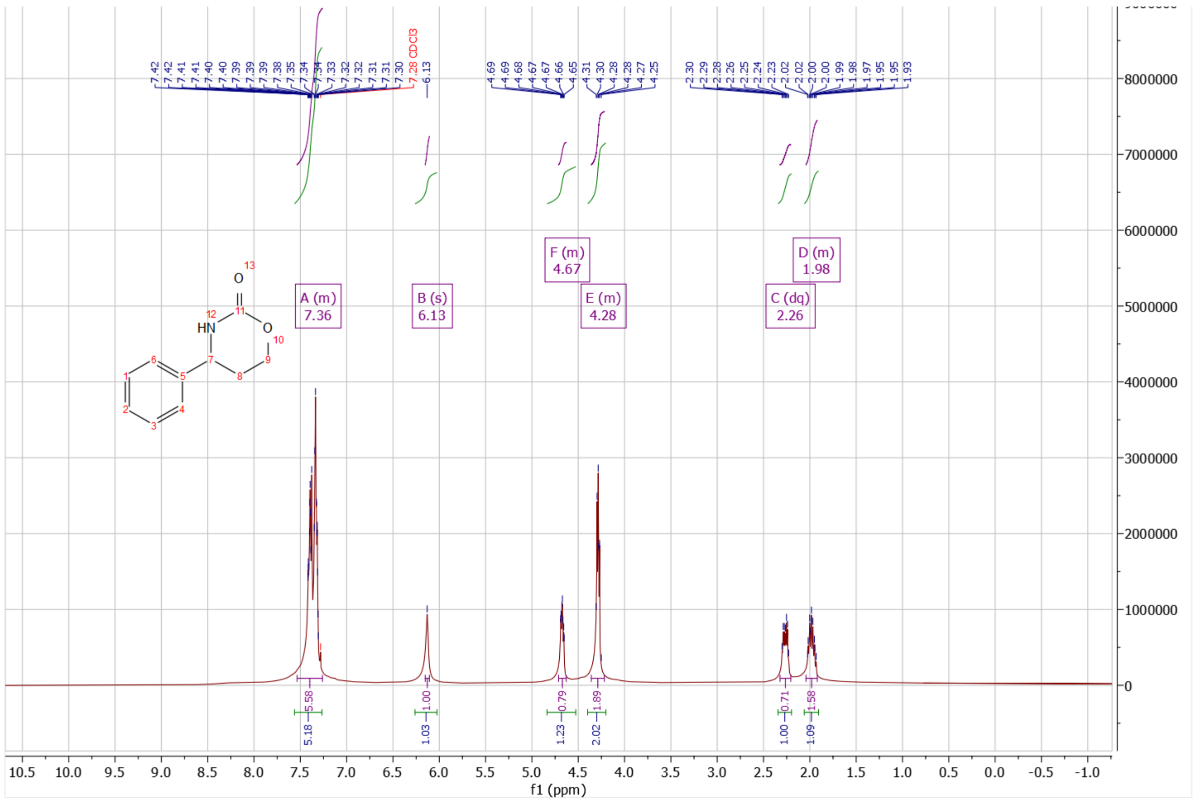

*Figure S42. $^{1}$H NMR spectra of **2t**.*

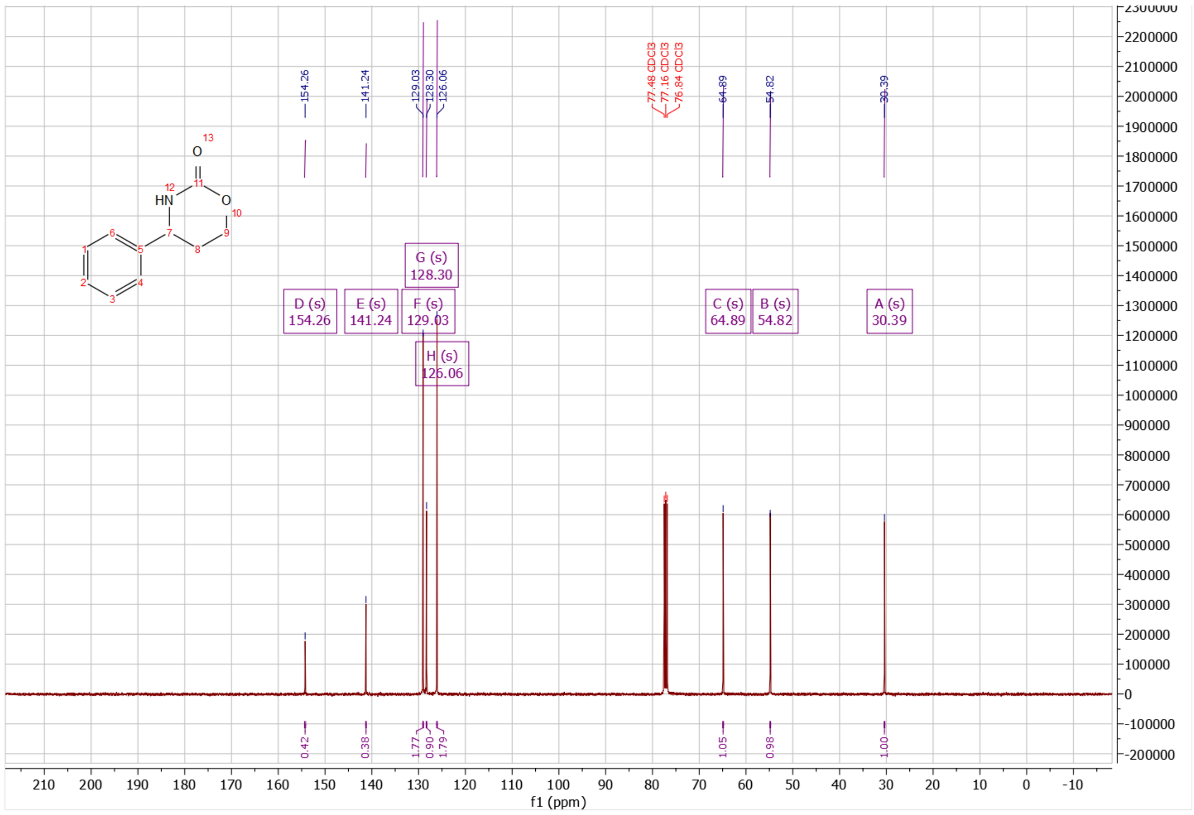

*Figure S43. $^{13}$C NMR spectra of **2t**.*

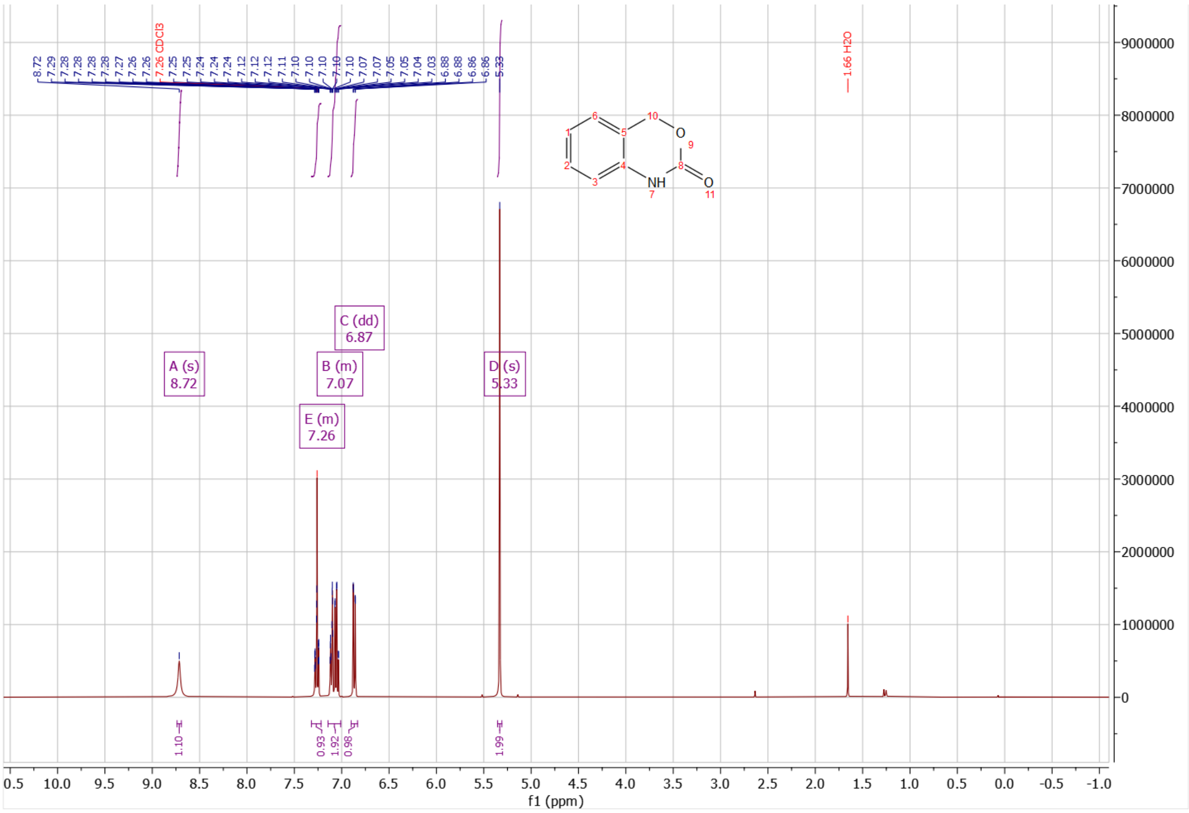

*Figure S44. $^{1}$H NMR spectra of **2u**.*

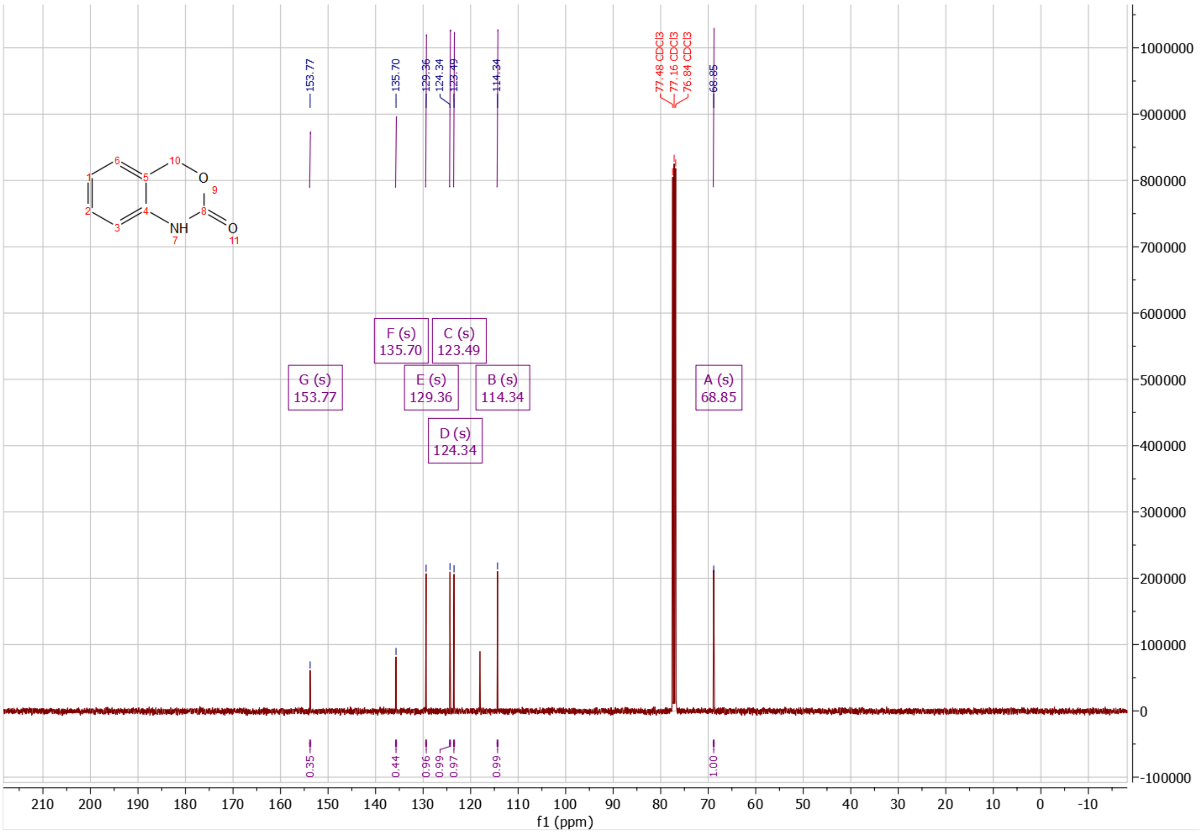

*Figure S45. $^{13}$C NMR spectra of **2u**.*

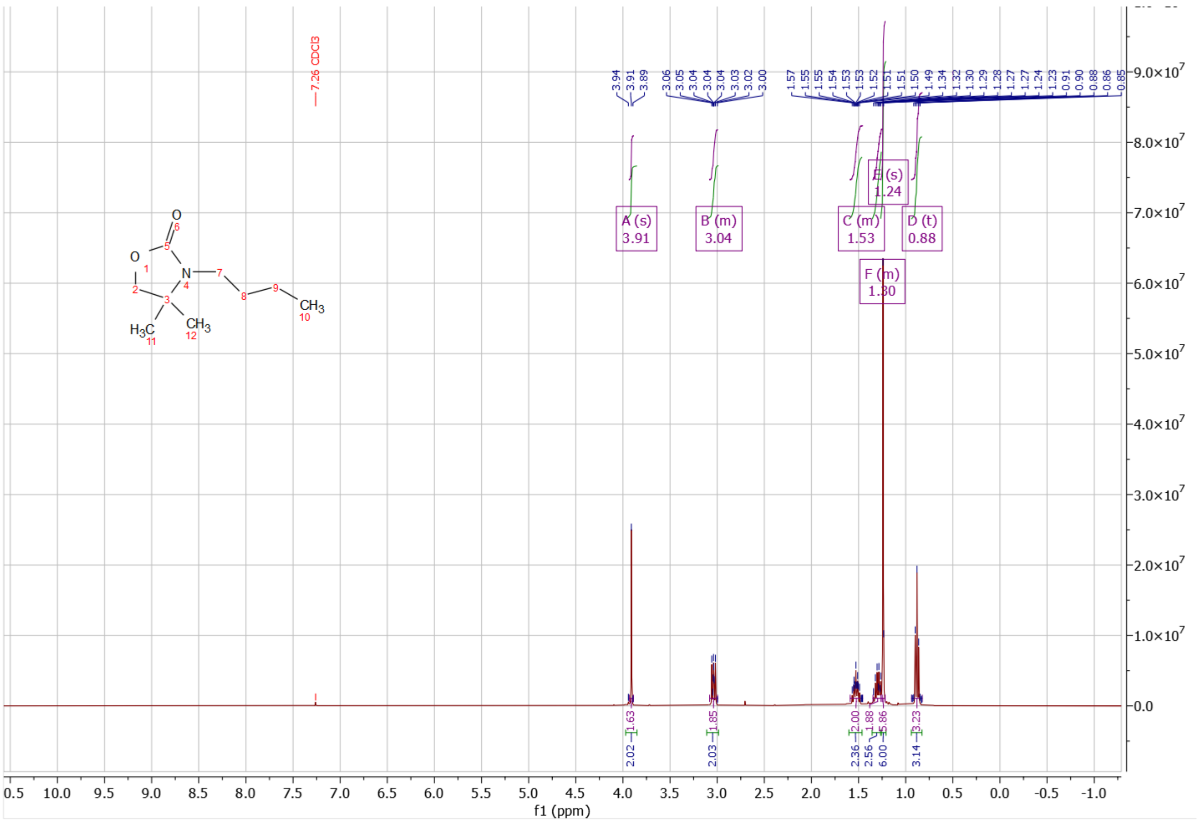

*Figure S46. $^{1}$H NMR spectra of **2v**.*

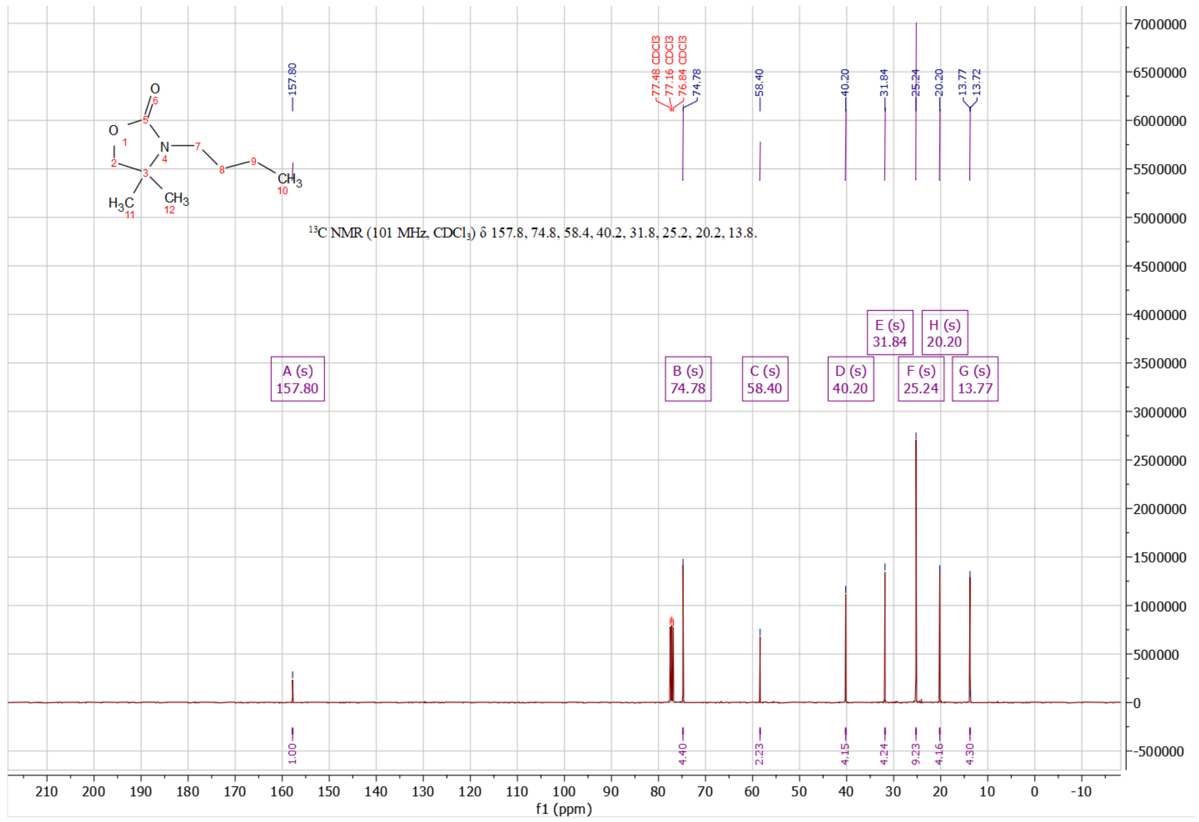


*Figure S47. $^{13}$C NMR spectra of **2v**.*

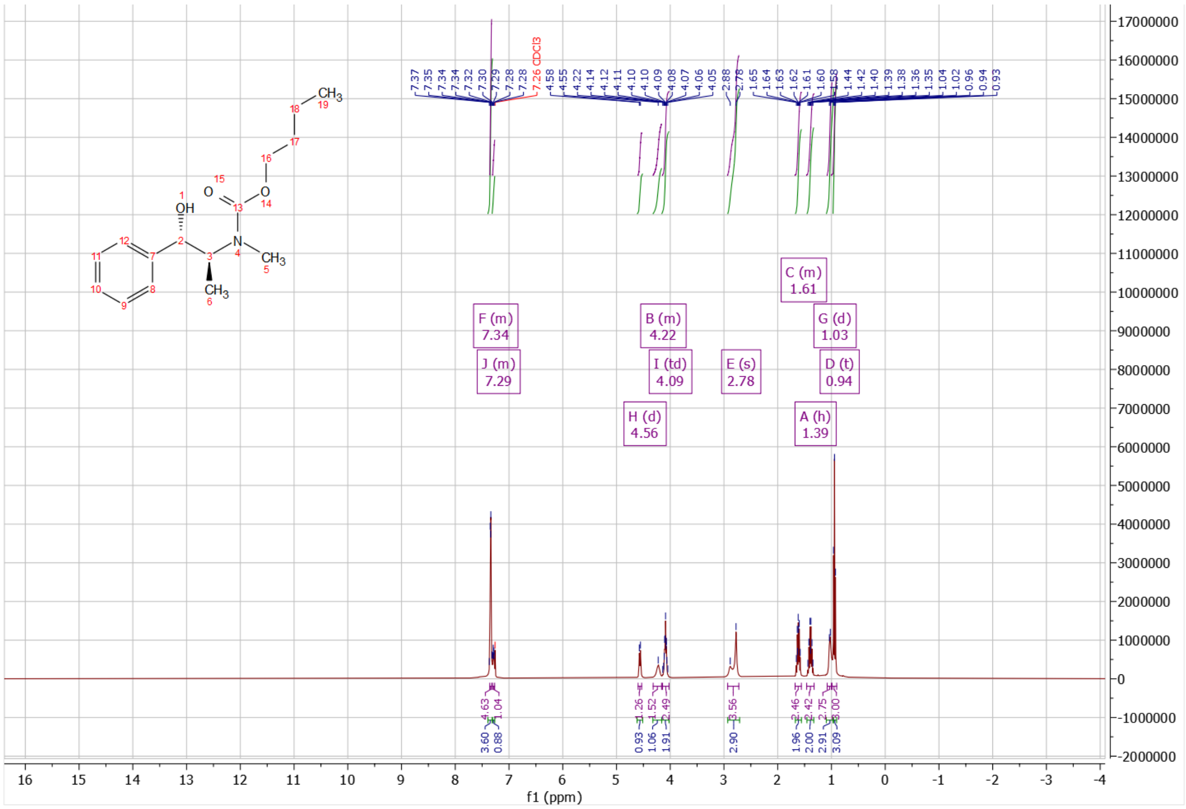

*Figure S48.* [1]*H NMR spctra of* ***4a****.*

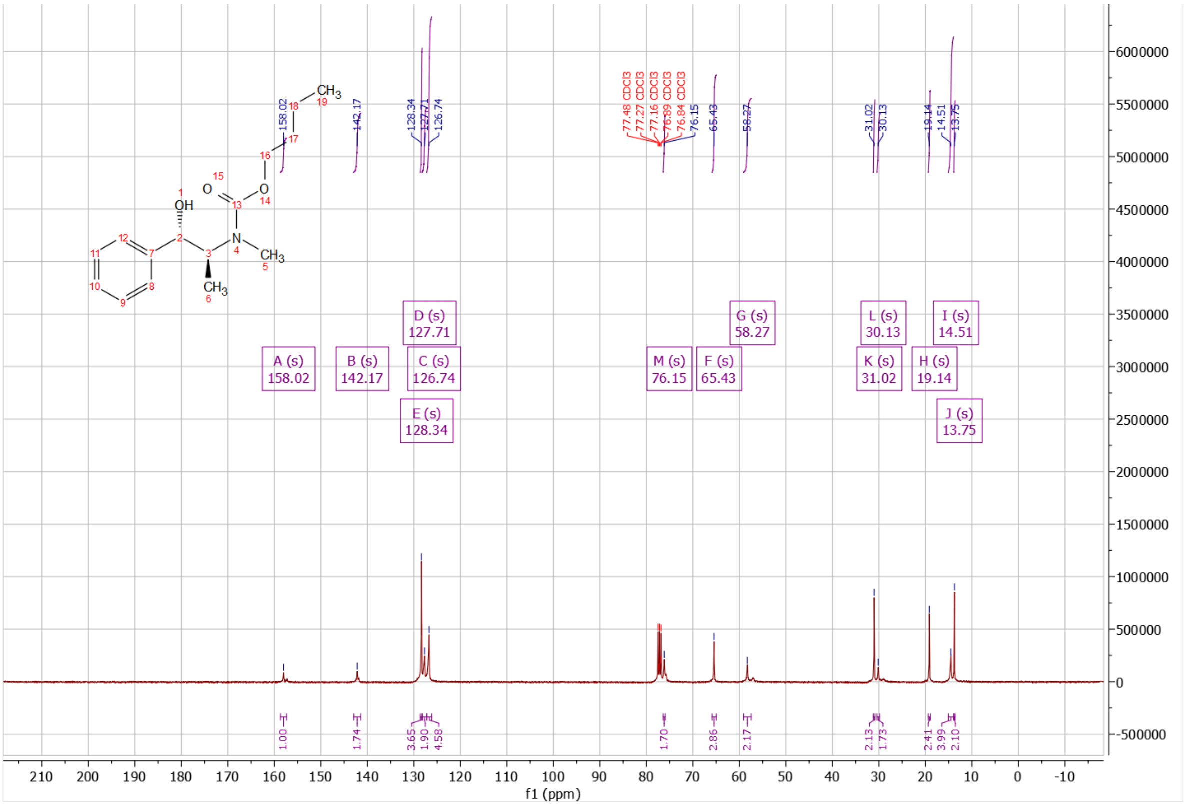

*Figure S49.* [13]*C NMR spectra of* ***4a****.*

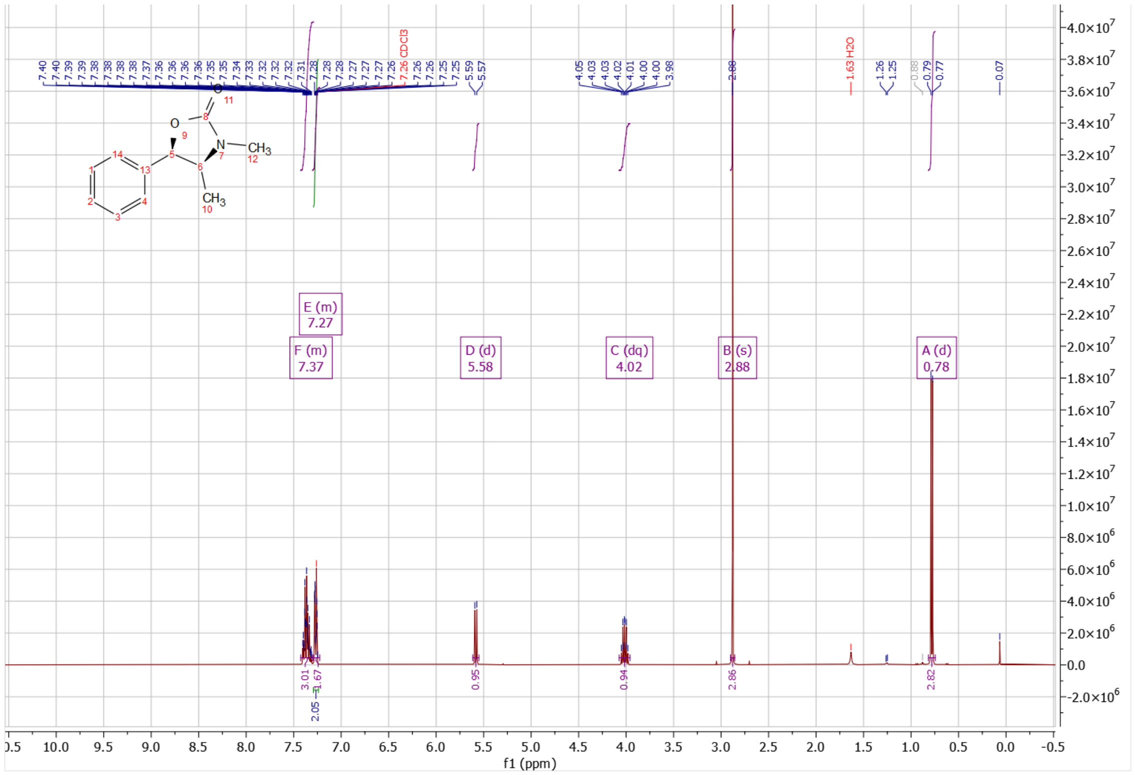

*Figure S50.* [1]*H NMR spectra of* ***2a'***.

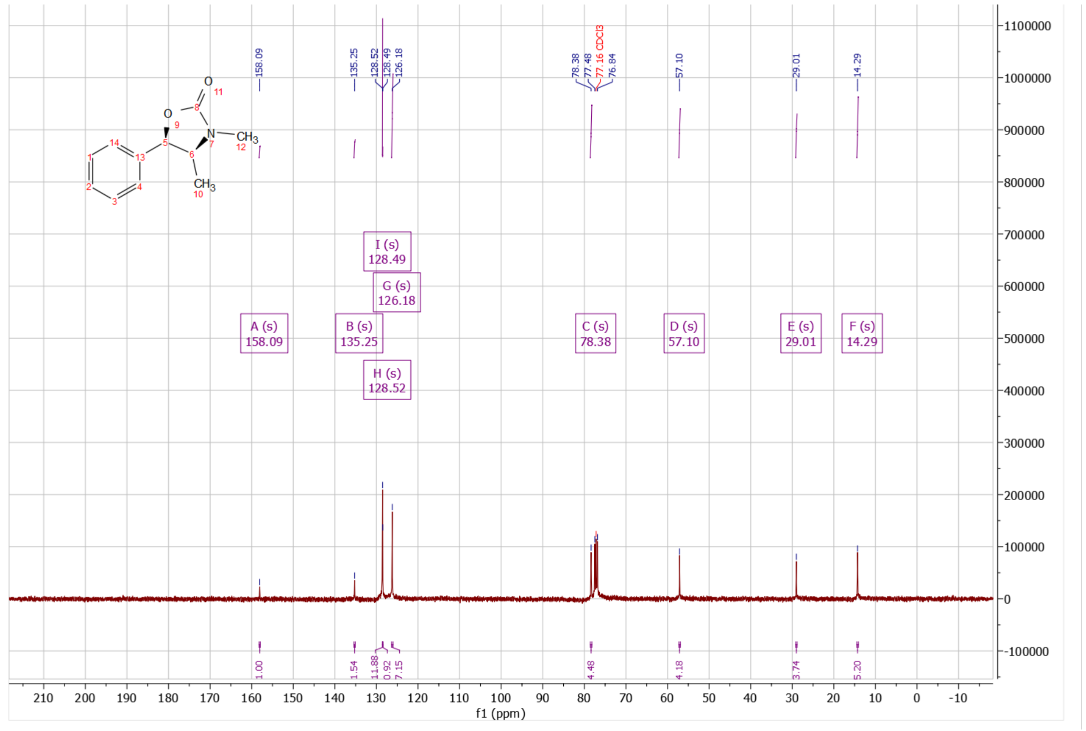

*Figure S51.* [13]*C NMR spectra of* ***2a'***.

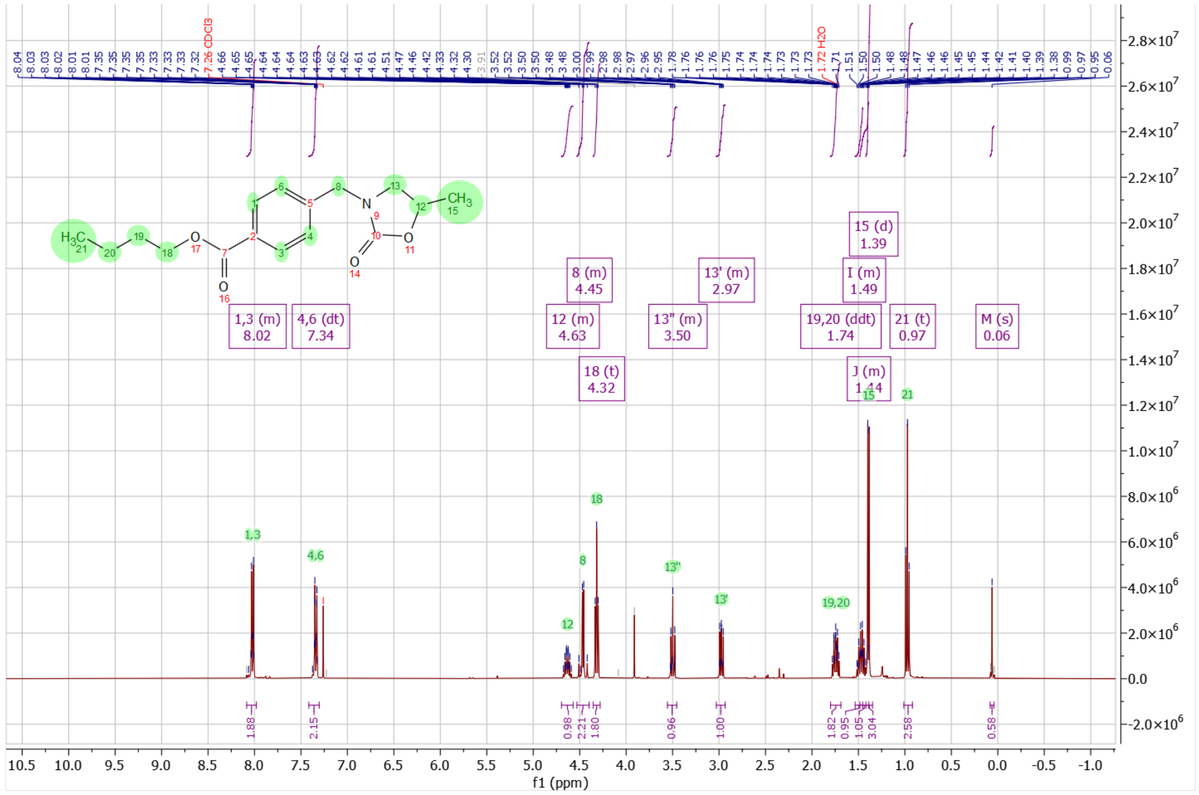

*Figure S52. $^{1}$H NMR spectra of **6**.*

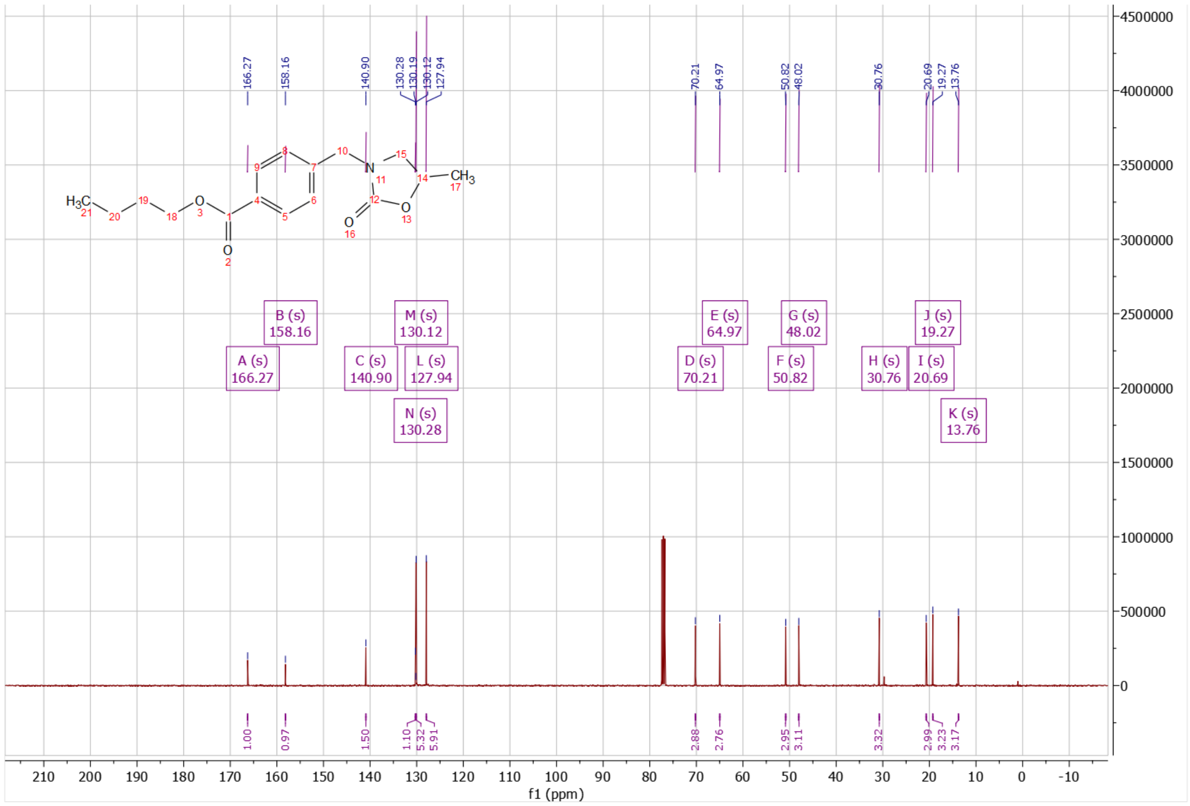

*Figure S53. $^{13}$C NMR spectra of **6**.*

## 9.2 AMINO ALCOHOLS

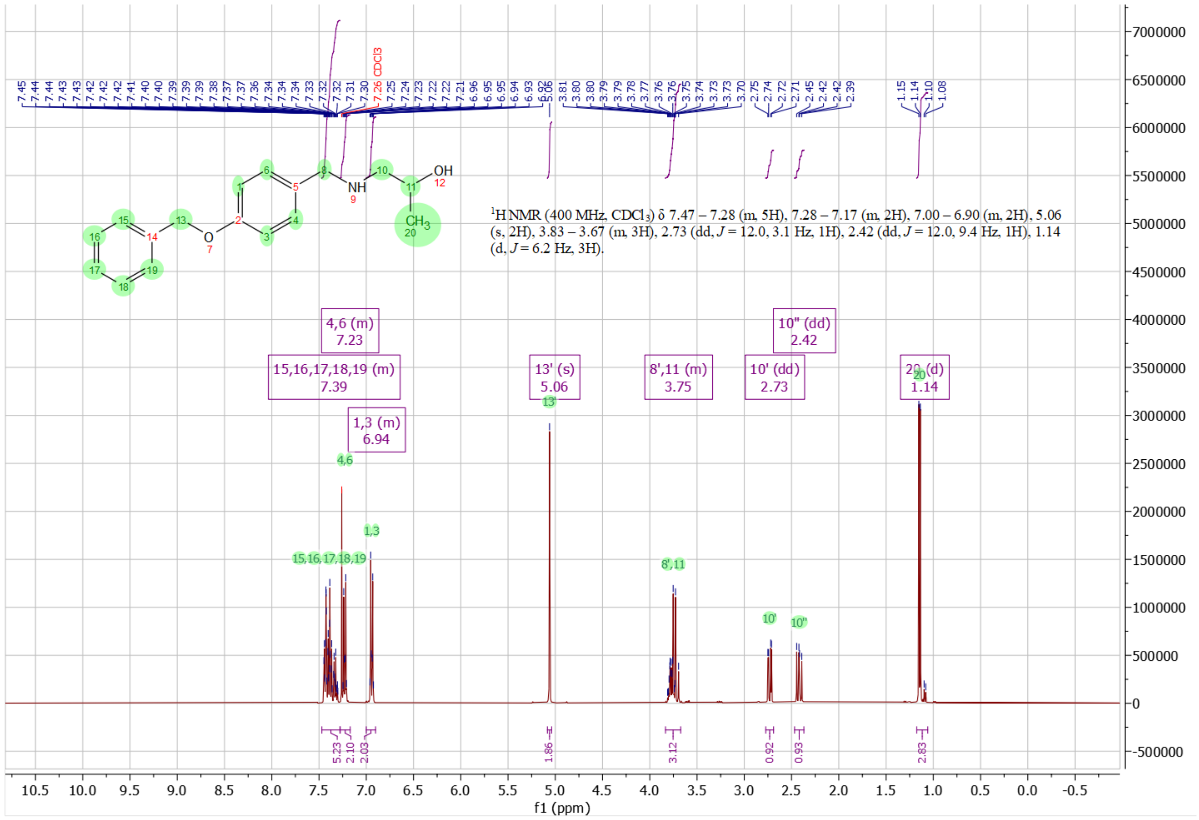


Figure S54. $^{1}$H NMR spectra of **1f**.

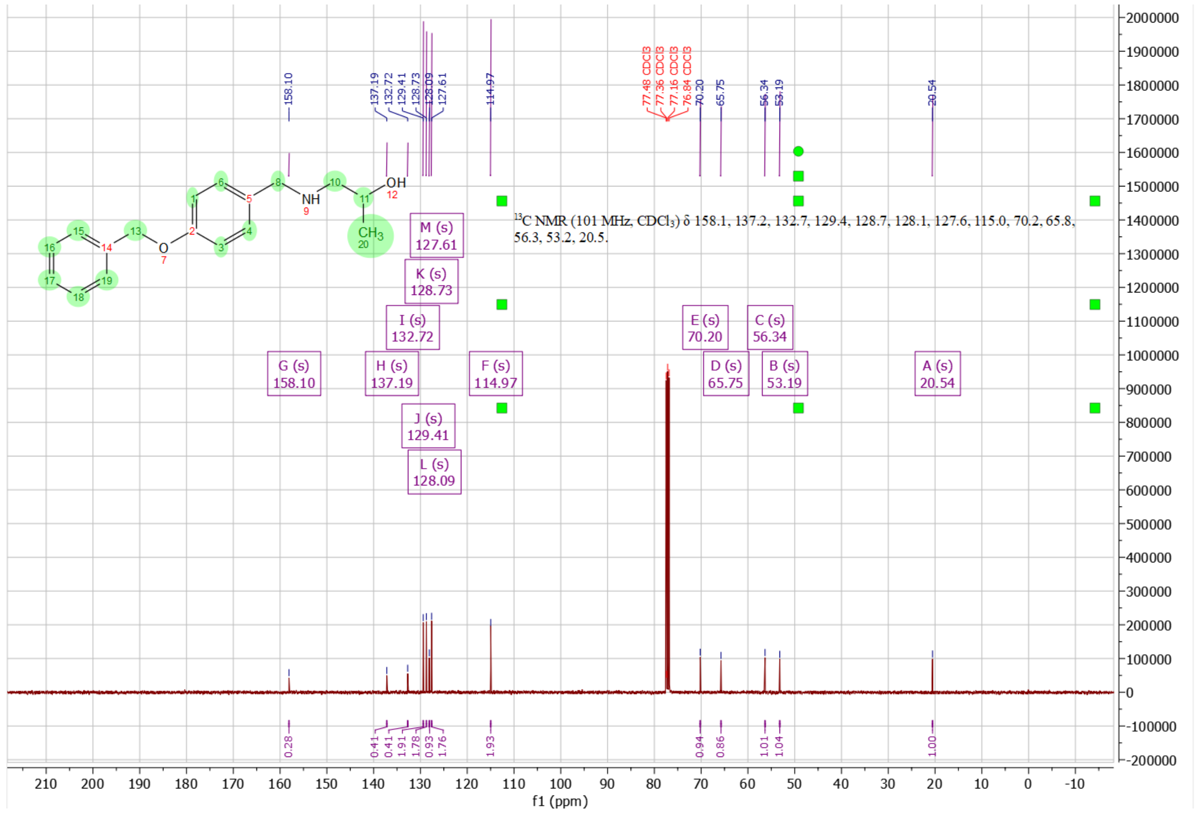


Figure S55. $^{13}$C NMR spectra of **1f**.

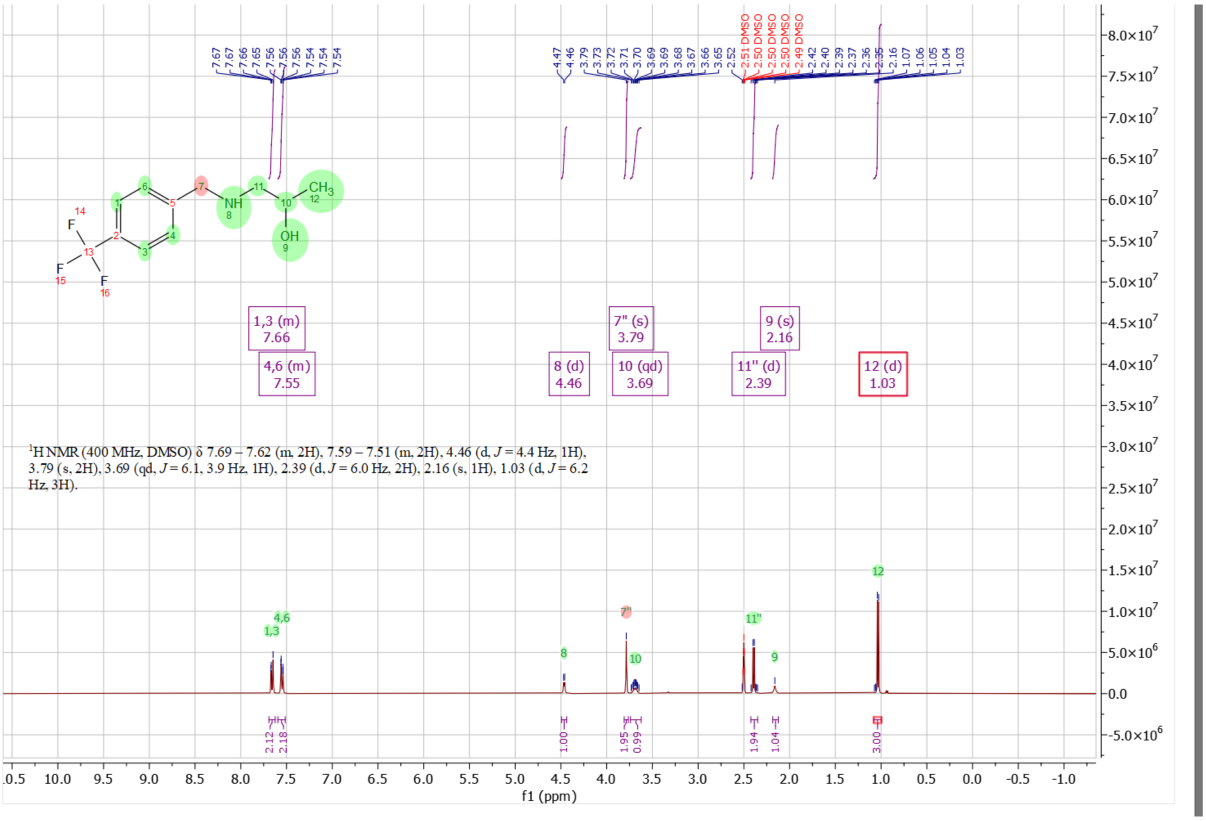


*Figure S56. [1]h NMR spectra of* ***1g****.*

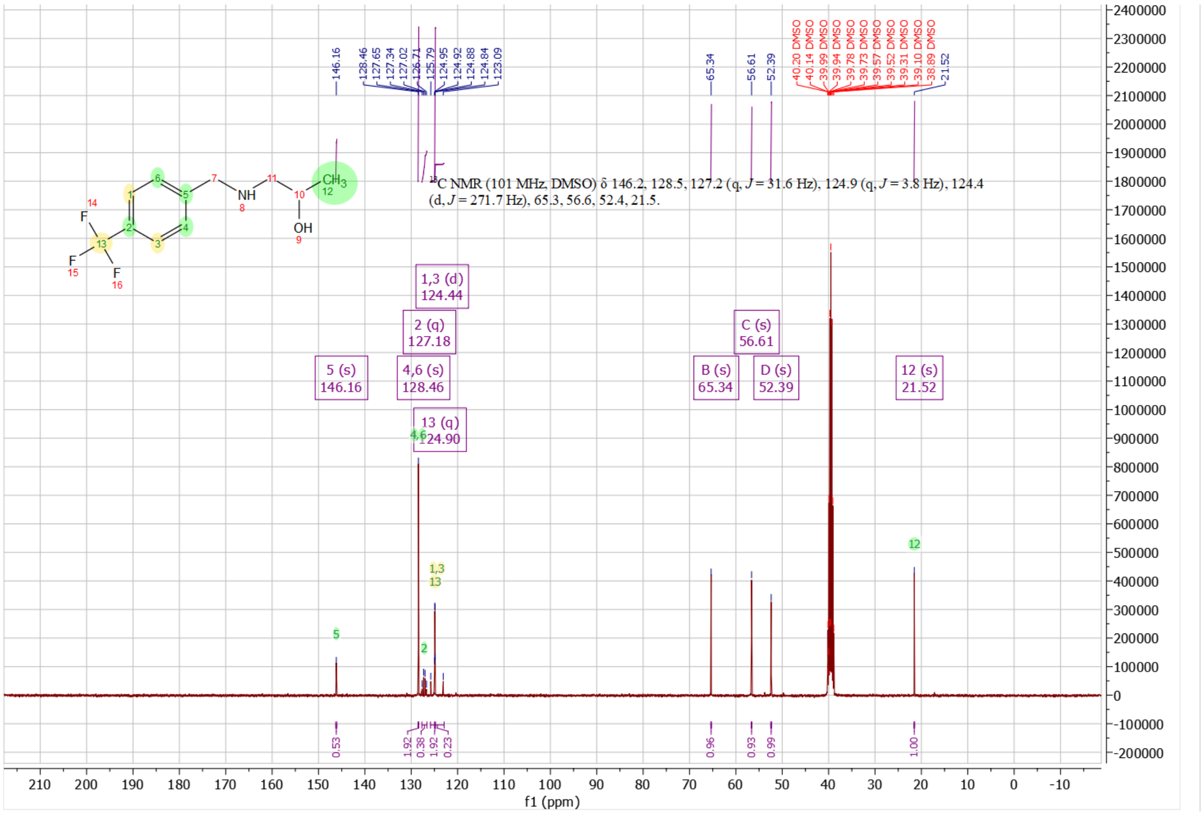


*Figure S57. $^{13}$C NMR spcetra of* ***1g****.*

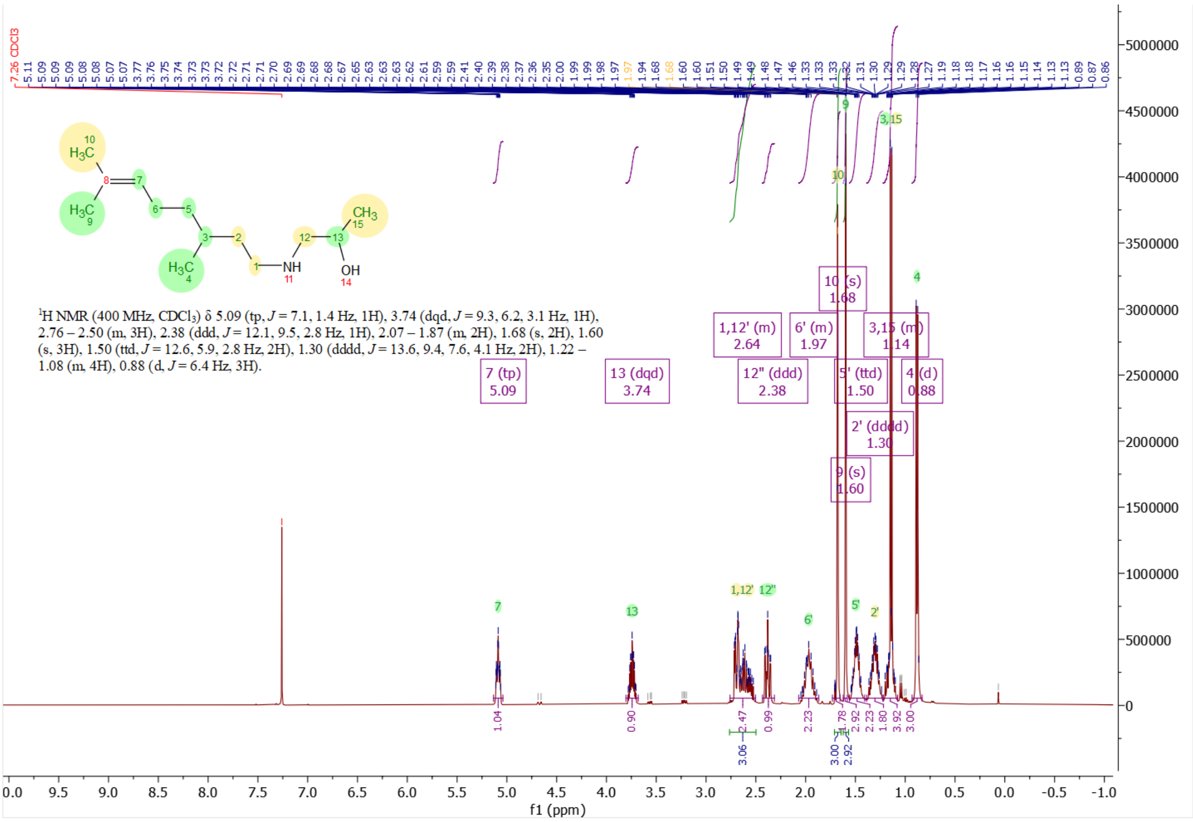


*Figure S58. $^{1}$H NMR spectra of **1h**.*

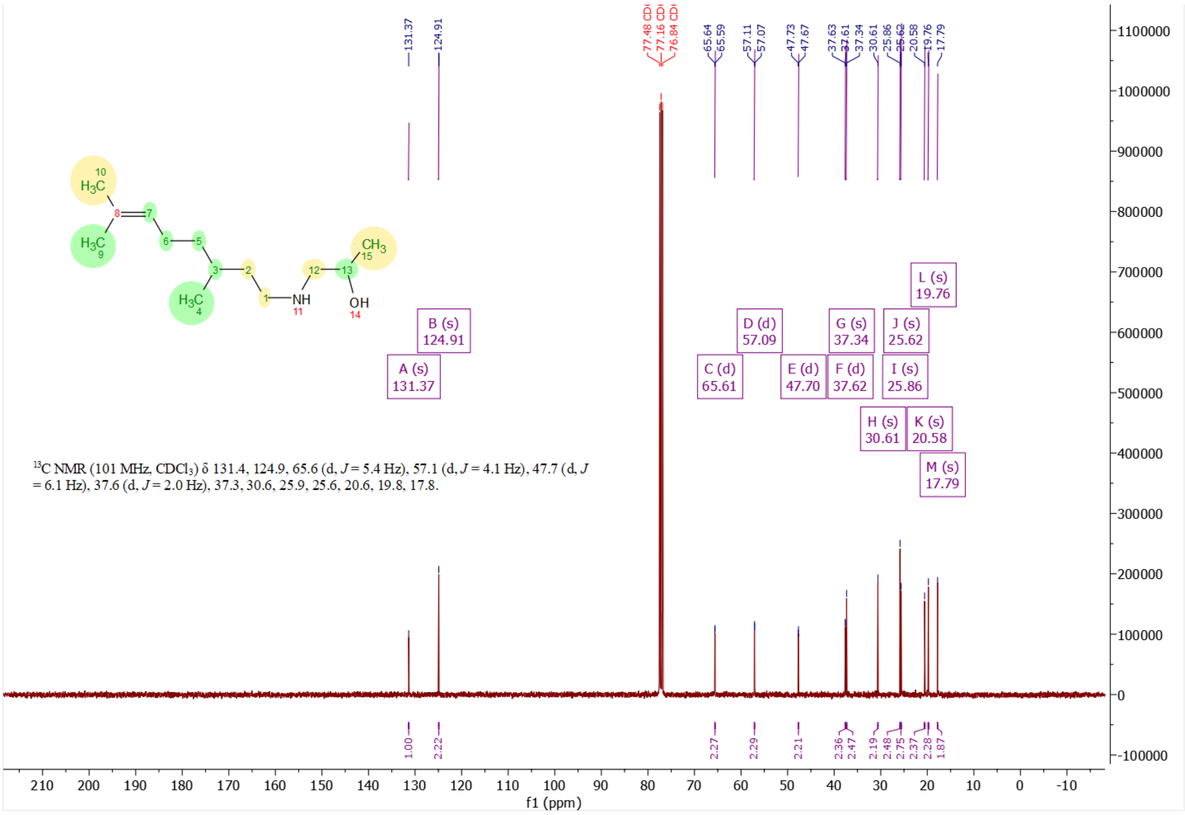


*Figure S59. $^{13}$ C NMR spectra of **1h**.*

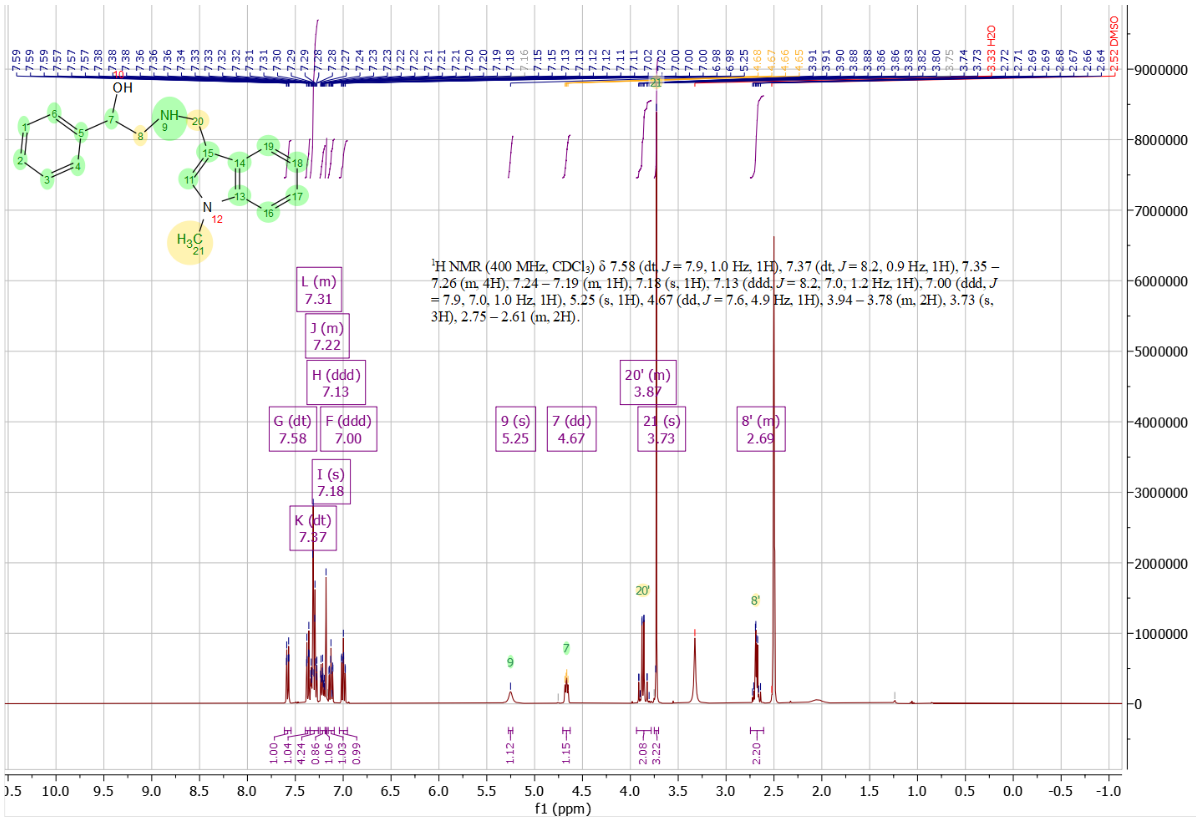


*Figure S60. [1] NMR spectra of **1i**.*

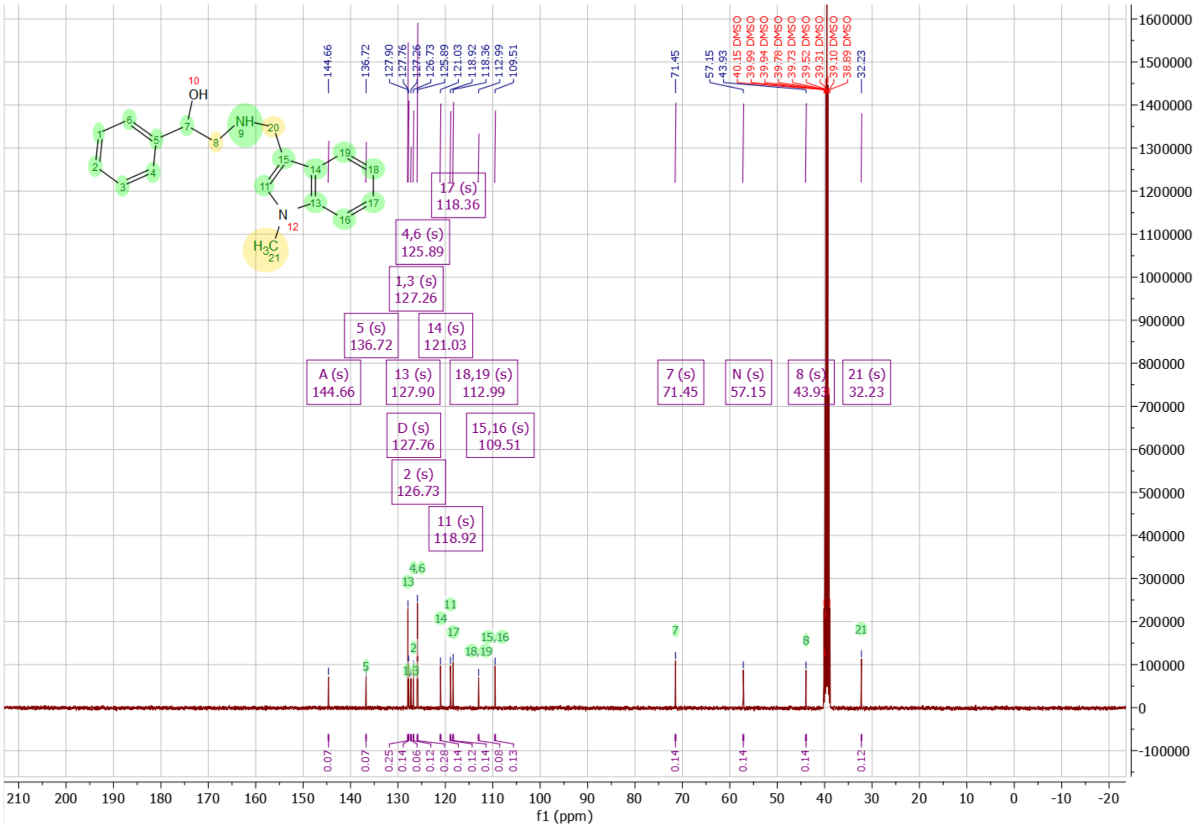


*Figure S61. $^{13}$C NMR spectra of **1i**.*

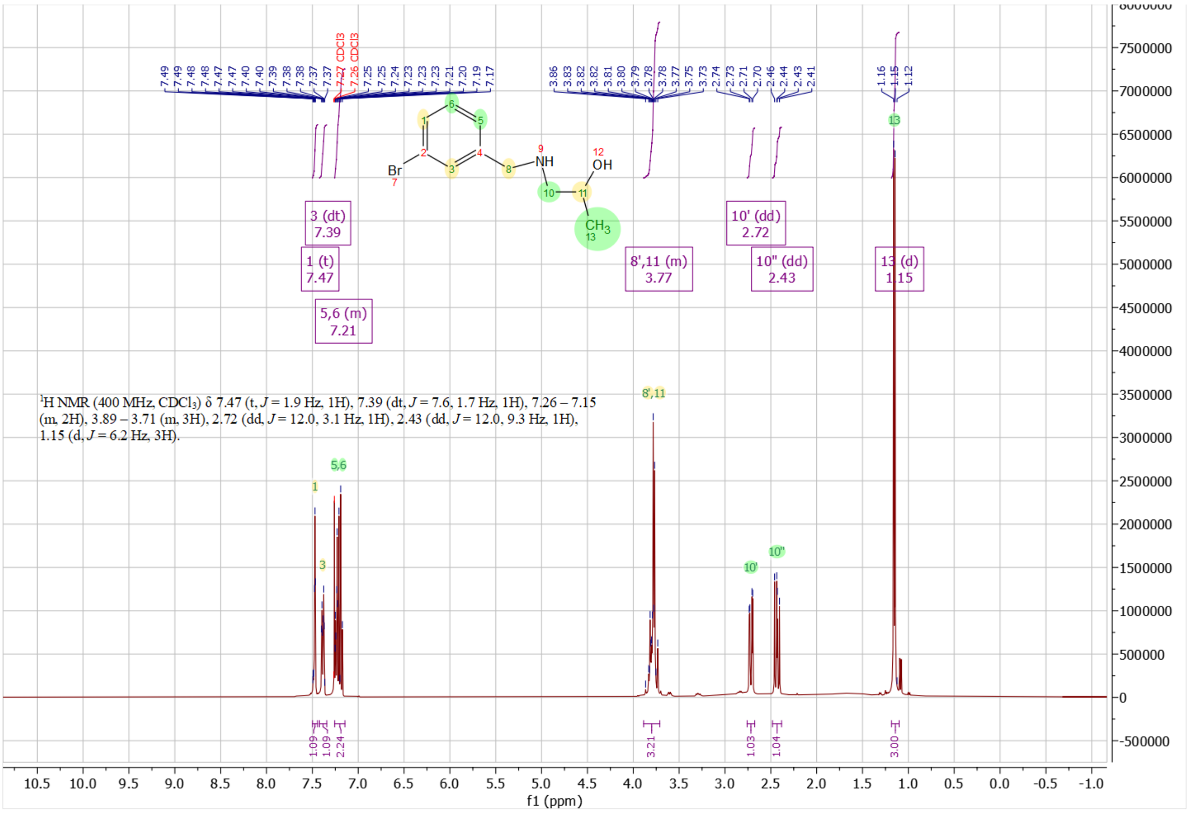


*Figure S62. ¹H NMR spectra of* ***1j****.*

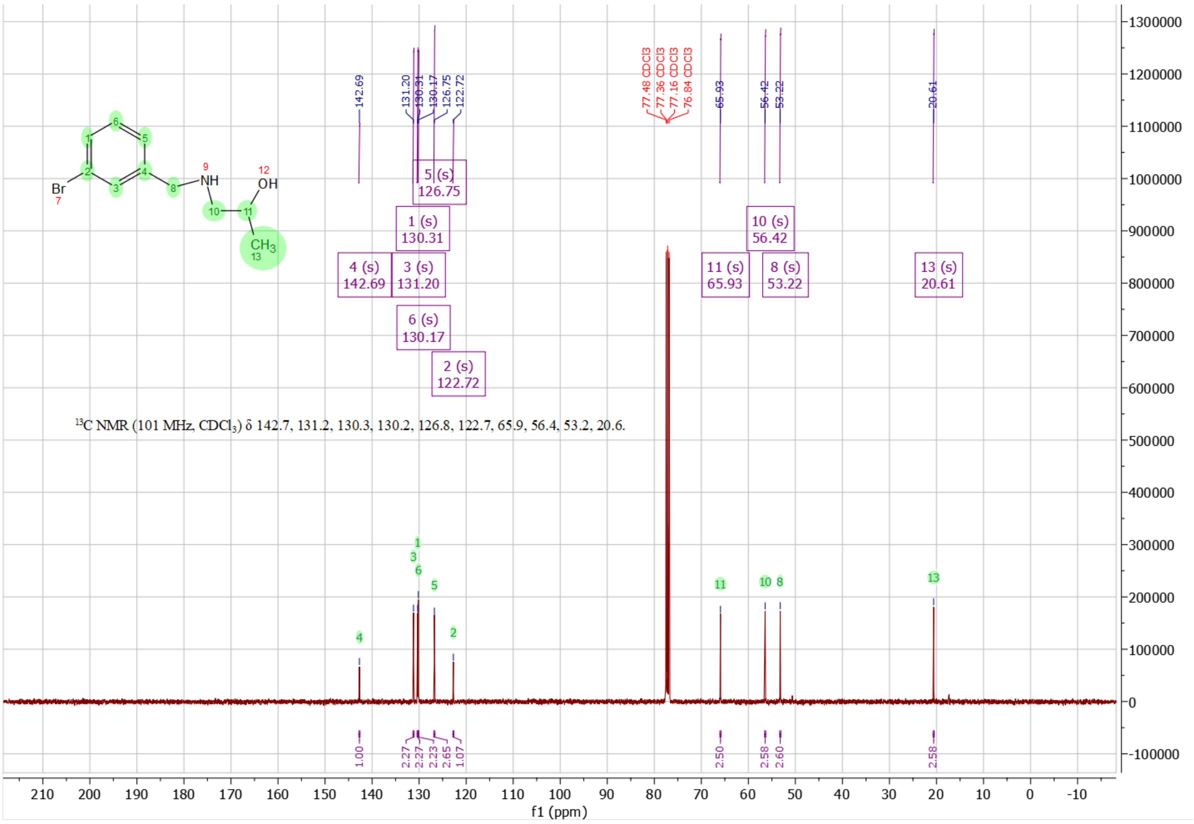


*Figure S63. ¹³C NMR spectra of* ***1j****.*

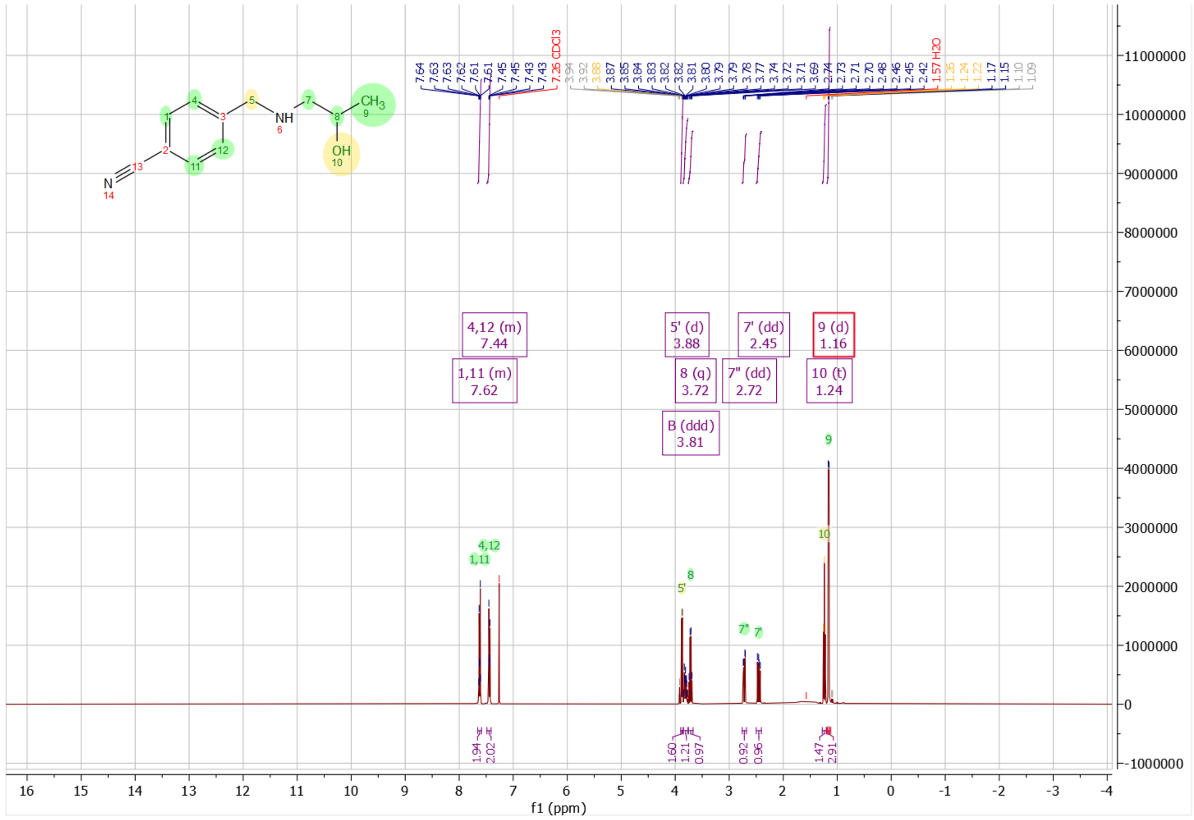

*Figure S64. $^{1}$H NMR spectra of **1w**.*

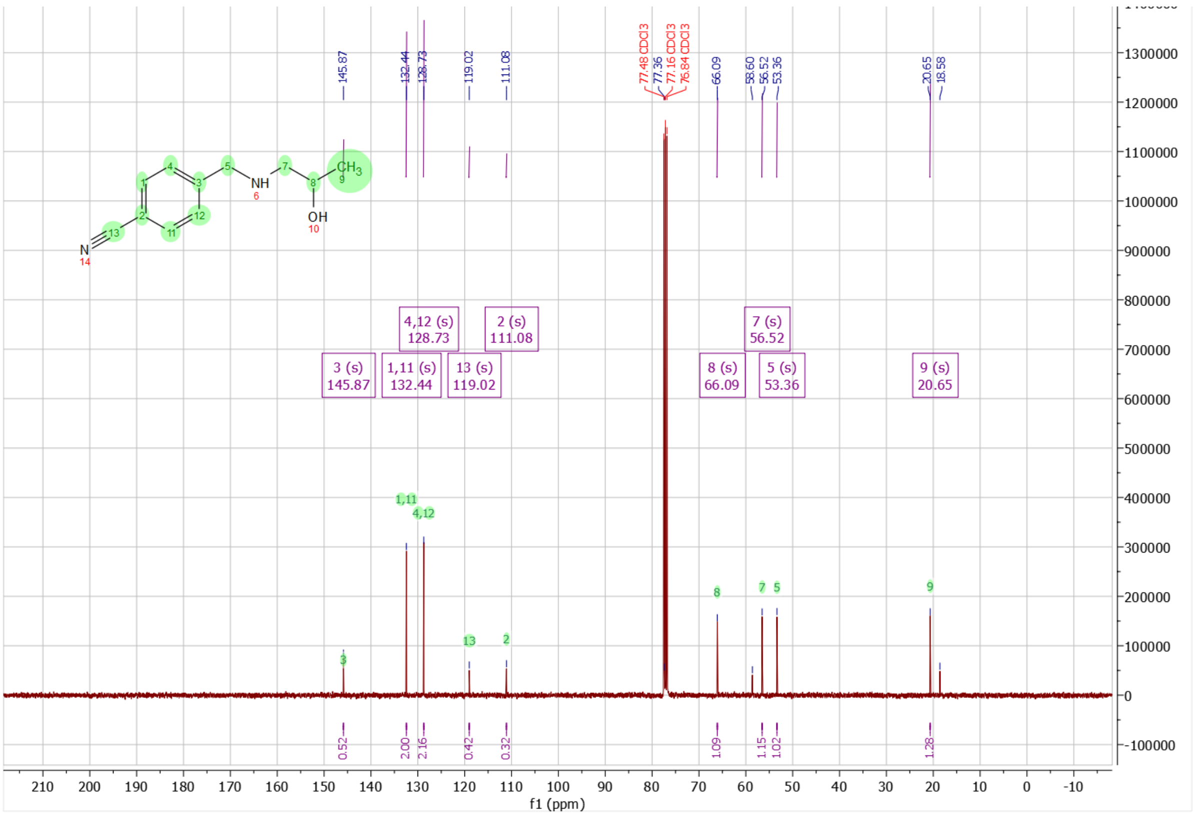

*Figure S65. $^{13}$C NMR spectra of **1w**.*

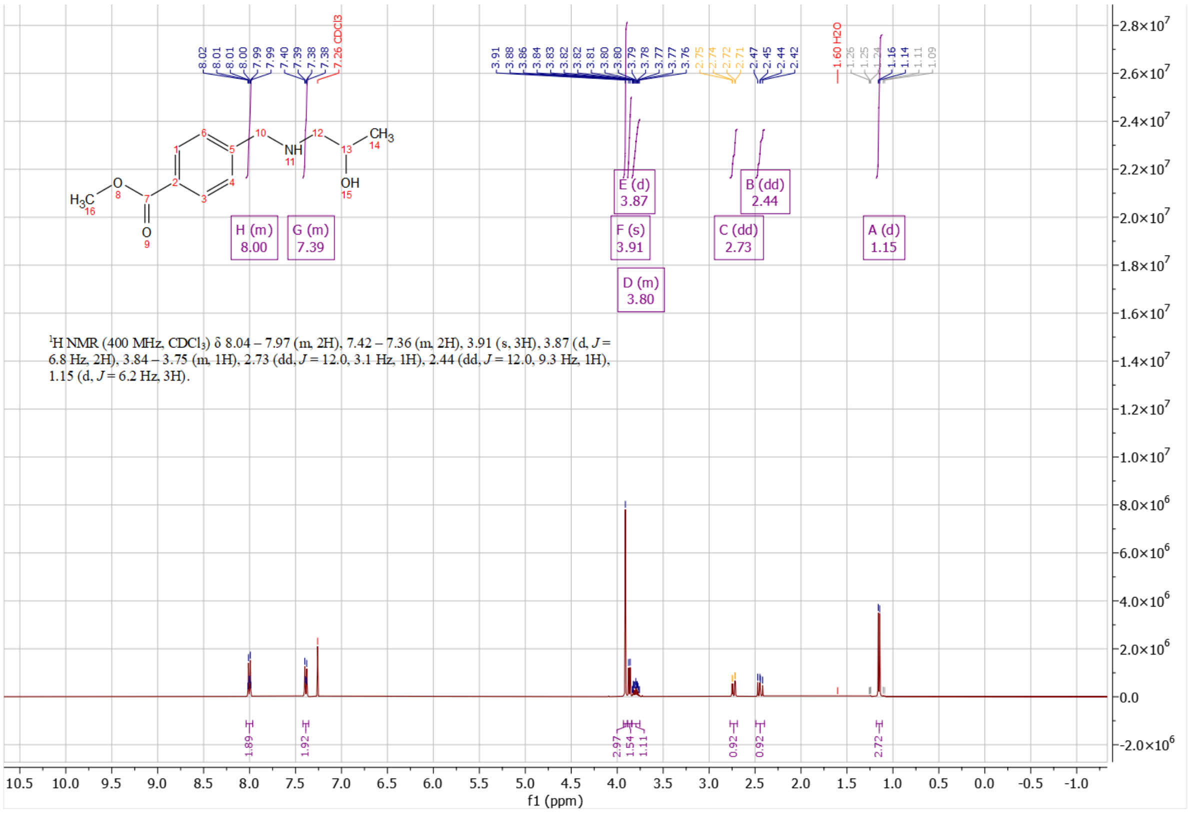


*Figure S66. $^{1}$H NMR spectra of **1x**.*

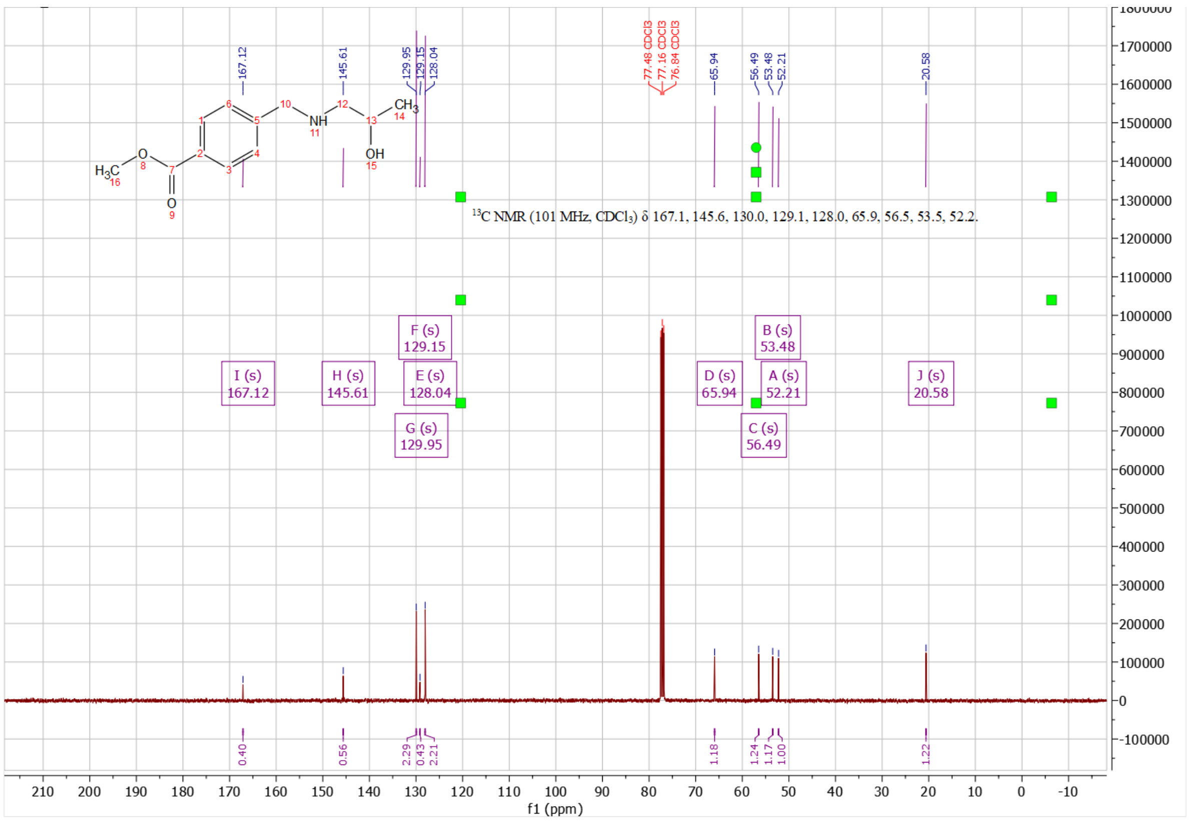


*Figure S67. $^{13}$C NMR specra of **1x**.*

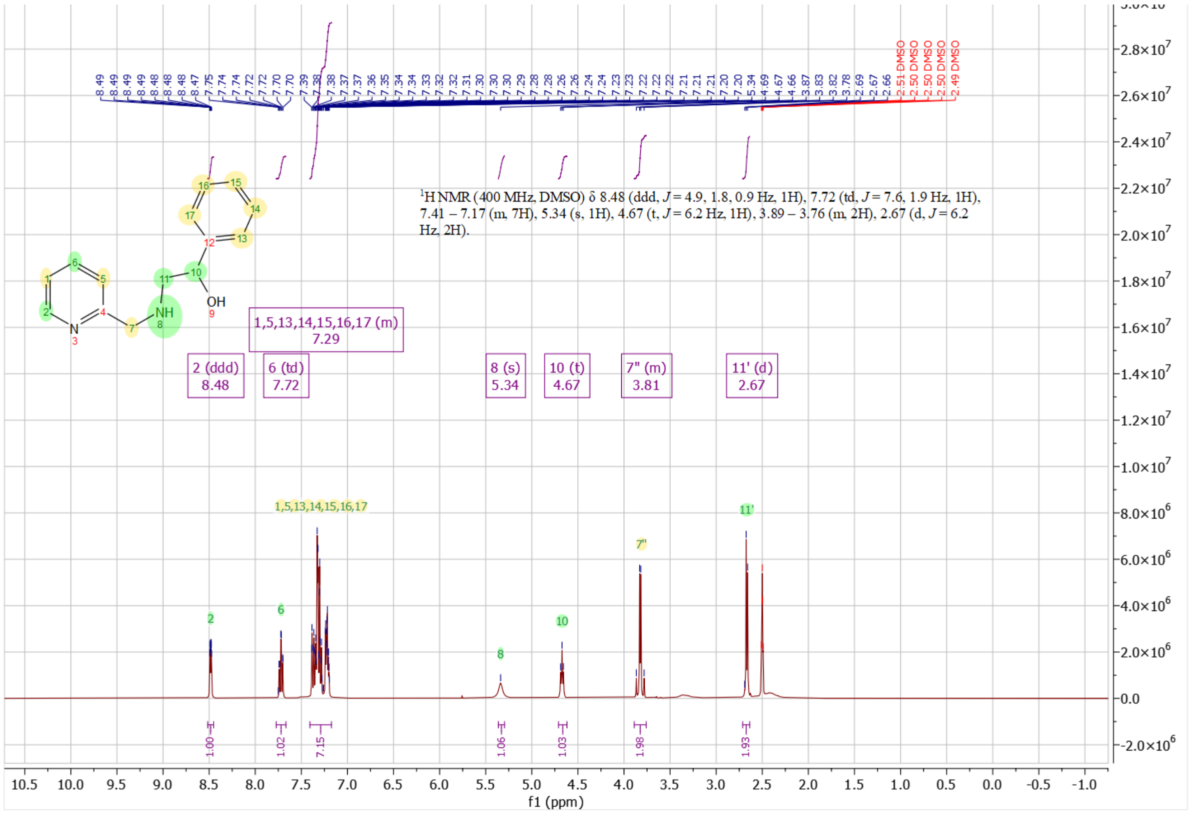


*Figure S68. ¹H NMR spectra of **1y**.*

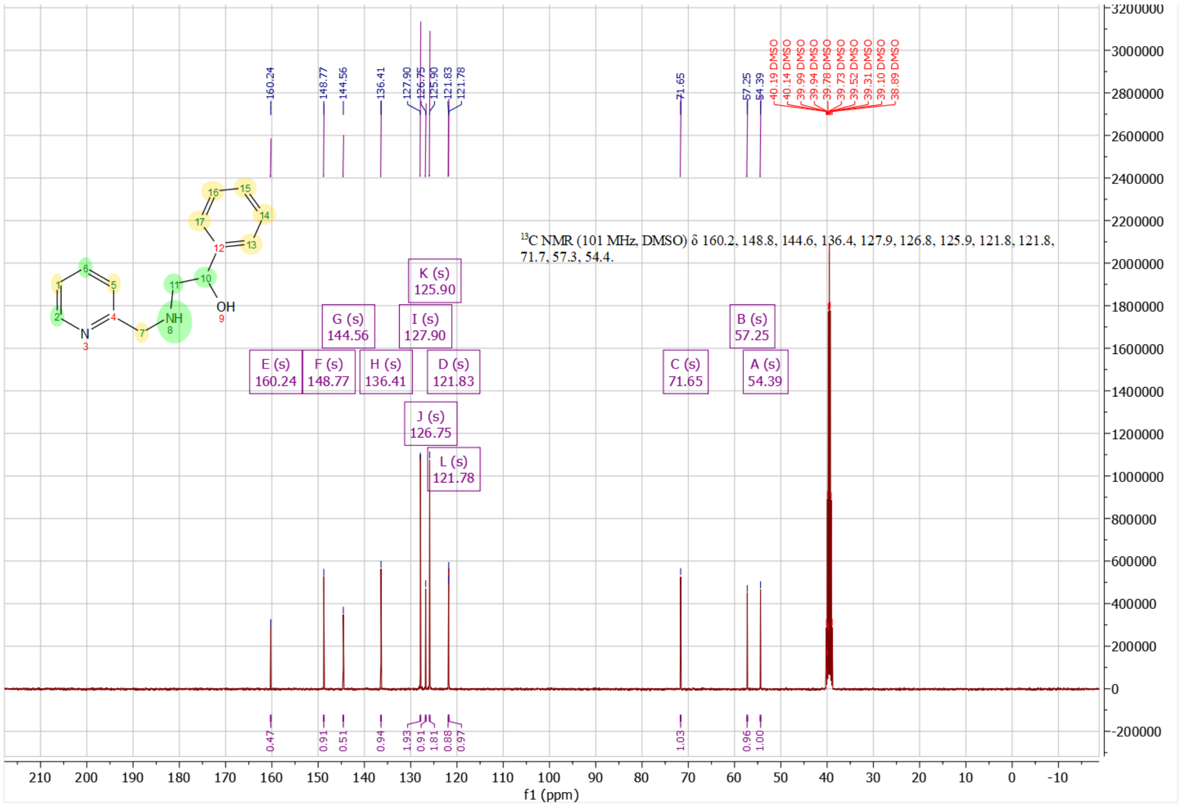


*Figure S69. ¹³C NMR spcetra of **1y**.*

# 10 REFERENCES

(1) Gottlieb, H. E.; Kotlyar, V.; Nudelman, A. NMR Chemical Shifts of Common Laboratory Solvents as Trace Impurities. *The Journal of Organic Chemistry* **1997**, *62* (21), 7512-7515. DOI: 10.1021/jo971176v.

(2) Kortunov, P. V.; Baugh, L. S.; Siskin, M.; Calabro, D. C. In Situ Nuclear Magnetic Resonance Mechanistic Studies of Carbon Dioxide Reactions with Liquid Amines in Mixed Base Systems: The Interplay of Lewis and Brønsted Basicities. *Energy Fuels* **2015**, *29* (9), 5967-5989. DOI: 10.1021/acs.energyfuels.5b00988.

(3) Huy, P. H.; Filbrich, I. A General Catalytic Method for Highly Cost- and Atom-Efficient Nucleophilic Substitutions. *Chemistry – A European Journal* **2018**, *24* (29), 7410-7416. DOI: https://doi.org/10.1002/chem.201800588 (acccessed 2024/09/03).

(4) Shang, J.; Li, Z.; Su, C.; Guo, Y.; Deng, Y. Efficient synthesis of 2-oxazolidinones from epoxides and carbamates catalyzed by amine-functionalized ionic liquids. *RSC Advances* **2015**, *5* (88), 71765-71769, 10.1039/C5RA09838F. DOI: 10.1039/C5RA09838F.

(5) Mei, C.; Zhao, Y.; Zou, K.; Cao, C.; Pang, G.; Shi, Y. Synthesis of N-aryl-2-oxazolidinones from cyclic carbonates and aromatic amines catalyzed by bio-catalyst. *Research on Chemical Intermediates* **2018**, *44* (3), 2179-2194. DOI: 10.1007/s11164-017-3222-y.

(6) Wu, F.-t.; Wu, L.; Cui, C.-n. The catalytic system 'Rhodamine B/additive' for the chemical fixation of CO2. *Tetrahedron* **2021**, *83*, 131965. DOI: https://doi.org/10.1016/j.tet.2021.131965.

(7) Niemi, T.; Perea-Buceta, J. E.; Fernández, I.; Hiltunen, O.-M.; Salo, V.; Rautiainen, S.; Räisänen, M. T.; Repo, T. A One-Pot Synthesis of N-Aryl-2-Oxazolidinones and Cyclic Urethanes by the Lewis Base Catalyzed Fixation of Carbon Dioxide into Anilines and Bromoalkanes. *Chemistry – A European Journal* **2016**, *22* (30), 10355-10359. DOI: https://doi.org/10.1002/chem.201602338 (acccessed 2025/03/17).

(8) Zhong, Z.; Wu, H.; Chen, X.; Luo, Y.; Yang, L.; Feng, X.; Liu, X. Visible-Light-Promoted Enantioselective Acylation and Alkylation of Aldimines Enabled by 9-Fluorenone Electron-Shuttle Catalysis. *Journal of the American Chemical Society* **2024**, *146* (29), 20401-20413. DOI: 10.1021/jacs.4c06044.

(9) Wan, N.; Zhou, X.; Ma, R.; Tian, J.; Wang, H.; Cui, B.; Han, W.; Chen, Y. Synthesis of Chiral 5-Aryl-2-oxazolidinones via Halohydrin Dehalogenase-Catalyzed Enantio- and Regioselective Ring-Opening of Styrene Oxides. *Advanced Synthesis & Catalysis* **2020**, *362* (5), 1201-1207. DOI: https://doi.org/10.1002/adsc.201901412 (acccessed 2025/03/19).

(10) Hatano, M.; Yamashita, K.; Ishihara, K. C- and N-Selective Grignard Addition Reactions of α-Aldimino Esters in the Presence or Absence of Zinc(II) Chloride: Synthetic Applications to Optically Active Azacycles. *Organic Letters* **2015**, *17* (10), 2412-2415. DOI: 10.1021/acs.orglett.5b00927.

(11) Jeong, J.; Jung, H.; Kim, D.; Chang, S. Multidimensional Screening Accelerates the Discovery of Rhodium Catalyst Systems for Selective Intra- and Intermolecular C–H Amidations. *ACS Catalysis* **2022**, *12* (13), 8127-8138. DOI: 10.1021/acscatal.2c02612.

(12) Dahiya, P.; Sarkar, A.; Sundararaju, B. Well-Defined [Cp*Co(N,O)I]-Catalysts for Site-Selective Intramolecular C−H Amidation. *Advanced Synthesis & Catalysis* **2022**, *364* (15), 2642-2647. DOI: https://doi.org/10.1002/adsc.202200385 (acccessed 2025/03/17).

(13) Bogdan, A. R.; Charaschanya, M.; Dombrowski, A. W.; Wang, Y.; Djuric, S. W. High-Temperature Boc Deprotection in Flow and Its Application in Multistep Reaction Sequences. *Organic Letters* **2016**, *18* (8), 1732-1735. DOI: 10.1021/acs.orglett.6b00378.

(14) Agami, C.; Couty, F.; Hamon, L.; Venier, O. Chiral oxazolidinones from N-Boc derivatives of β-amino alcohols. Effect of a N-methyl substituent on reactivity and

stereoselectivity. *Tetrahedron Letters* **1993**, *34* (28), 4509-4512. DOI: https://doi.org/10.1016/0040-4039(93)88071-P.
(15) Paz, J.; Pérez-Balado, C.; Iglesias, B.; Muñoz, L. Carbon Dioxide as a Carbonylating Agent in the Synthesis of 2-Oxazolidinones, 2-Oxazinones, and Cyclic Ureas: Scope and Limitations. *The Journal of Organic Chemistry* **2010**, *75* (9), 3037-3046. DOI: 10.1021/jo100268n.
(16) Alalla, A.; Merabet-Khelassi, M.; Riant, O.; Aribi-Zouioueche, L. Easy kinetic resolution of some β-amino alcohols by Candida antarctica lipase B catalyzed hydrolysis in organic media. *Tetrahedron: Asymmetry* **2016**, *27* (24), 1253-1259. DOI: https://doi.org/10.1016/j.tetasy.2016.10.003.
